# Supplementary material for: Non-isocyanate polyurethanes synthesized from terpenes using thiourea organocatalysis and thiol-ene-chemistry
Source: Commun Chem. 2023 Nov 4;6:239. doi: 10.1038/s42004-023-01041-x (PMC10625552; doi:10.1038/s42004-023-01041-x)
Supplement: Supplementary file 2 — Supplementary Information [file 42004_2023_1041_MOESM2_ESM.pdf]

Supplementary Information

Non-Isocyanate Polyurethanes Synthesized from Terpenes Using Thiourea Organocatalysis and Thiol-Ene-Chemistry

Frieda Clara M. Scheelje,[1] Michael A. R. Meier[1,2]*

[1] Laboratory of Applied Chemistry, Institute of Organic Chemistry (IOC), Karlsruhe Institute of Technology (KIT), Straße am Forum 7, 76131 Karlsruhe, Germany.

[2] Laboratory of Applied Chemistry, Institute of Biological and Chemical Systems - Functional Molecular Systems (IBCS-FMS), Karlsruhe Institute of Technology (KIT), Hermann-von-Helmholtz-Platz 1, 76344 Eggenstein-Leopoldshafen, Germany.

* E-mail: m.a.r.meier@kit.edu

Table of Contents

1. Supplementary Methods 2
1.1 Materials 2
1.2 Instrumentation 2
2. Supplementary Note 1: Small-scale reaction monitoring 6
2.1 Carbonate formation 6
2.2 Carbonate opening reactions 6
2.3 Thiol-ene polymerization reactions 9
3. Supplementary Note 2: Characterization of NIPUs 11
3.1 Spectroscopic analysis of polymers 13
3.2 Thermal analysis of polymers 13
4. Supplementary Note 3: Monomer and catalyst syntheses 19
4.1 Synthesis of monomer 13 19
4.2 Synthesis of monomer 15 28
4.3 Synthesis of monomer 14 34
4.4 Synthesis of monomers 18 and 19 43
4.5 Synthesis of dithiol 22 57
4.6 Synthesis of thiourea catalysts 59
5. Supplementary References 72

1. Supplementary Methods

1.1 Materials

(*R*)-(+)-limonene (Sigma Aldrich, 97%), (*S*)-(+)-carvone (Sigma Aldrich, 98%), 1,10-decanedithiol (TCI, >98%), 1,4-butandedithiol (Sigma Aldrich, >97%), 1,5,7-triazabicyclo[4.4.0]dec-5-ene (TCI, >98%), 1,8-diazobicyclo[5.4.0]-7-undecene (TCI, >98.0%), 1-bromohexane (fluorochem), 2,2'-dimethoxy-2-phenylacetophenone (Sigma Aldrich, 99%), 2-methyl tetrahydrofuran (Acros Organics, anhydrous, stabilized), 4-aminothiophenol (fluorochem), 10-undecenoic acid (Sigma Aldrich, 98%), acetic acid (Fluka, >99.8%), acetone (Honeywell, ≥99.8%), acetonitrile (Fisher Scientific, HPLC gradient grade), allylamine (abcr, 98%), ammonium chloride (technical grade), chloroform (Fisher Scientific, >99.8%), chloroform-*d* (Eurisotop, 99.8% D), cyclohexane (VWR, HPLC grade), cyclohexyl amine (Sigma Aldrich, ≥99.9%), dichloromethane (Fisher Scientific, HPLC grade, ≥99.8%), diethyl ether (technical grade), diisopropyl amine (Sigma Aldrich, >99.5%), dimethyl carbonate (Sigma Aldrich, Y99%), dimethyl sulfoxide (Fisher Scientific, ≥99.9%), dimethyl sulfoxide-d_6 (Eurisotop, 99.80% D), ethanol (Fisher Scientific, ≥99.8%), ethyl acetate (VWR, HPLC grade), formic acid (Carl Roth, ≥98%), hydrogen peroxide solution (30%), hydroxylamine hydrochloride (Acros Organics, >99%), methanol (VWR, HPLC grade), *N*-bromo succinimide (abcr, 99%), *n*-hexane (technical grade), oxone® (Sigma Aldrich), phosphorus oxychloride (Acros Organics, 99%), potassium hydroxide (Bernd Kraft, for analysis), sodium bicarbonate (Sigma Aldrich, >95%), sodium chloride (Fisher Scientific, 99.5%), sodium hydroxide (Carl Roth, ≥99%), sodium sulfate anhydrous (Acros Organics, 99%), sodium sulfite (anhydrous), sulfur (elemental, technical grade), tetrabutylammonium bromide (Acros Organics, 98%), tetrabutylammonium chloride (Sigma Aldrich, ≥97.0%, tetrabutylammonium iodide (Sigma Aldrich, ≥99.0%), tetrahydrofuran (Sigma Aldrich, ≥99.9%), thioacetic acid (Alfa Aesar, 97%), toluene (Acros Organics, 99.85%), triethyl amine (Sigma Aldrich, ≥99.5%).

1.2 Instrumentation

Thin Layer Chromatography (TLC)

TLC was performed on aluminium plates coated with silica gel of the type 60 F_{254} from Sigma Aldrich. The compounds on the plates were visualised via fluorescence quenching with 254 nm UV-Light or by staining with Seebach solution (2 g cerium(IV) sulfate, 5 g phosphomolybdic acid hydrate, 16 mL concentrated sulfuric acid, 200 mL water) or vanillin solution (15 g vanillin, 2.5 mL concentrated sulfuric acid, 250 mL ethanol).

Flash Column Chromatography

Flash Column Chromatography was performed with silica gel 60 purchased from Sigma Aldrich. For automatic flash column chromatography, a Biotage® Isolera™ One Flash Column equipped with a UV-Vis detector (200 × 800 nm) with Biotage® Sfär Silica HC Duo Columns was used. All solvents were used in HPLC grade.

Nuclear Magnetic Resonance (NMR) Spectroscopy

^{1}H and ^{13}C NMR spectra were recorded with a Bruker AVANCE DPX 400 spectrometer at 400 MHz for ^{1}H NMR and 101 MHz for ^{13}C NMR at ambient temperature. Chemical shifts δ are reported in ppm relative to the solvent signal of $CDCl_3$ (7.26 ppm for ^{1}H and 77.16 ppm for ^{13}C spectra) or DMSO-d_6 (2.50 ppm for ^{1}H and 39.52 ppm for ^{13}C spectra). Spin multiplicity and corresponding signal patterns were reported as follows: s = singlet, d = doublet, t = triplet, q = quartet, m = multiplet and br = broadened. Coupling constants (*J*) are reported in Hertz (Hz). For full assignment of the signals to the measured structure, ^{1}H-^{1}H Correlated Spectroscopy (^{1}H-^{1}H-COSY), ^{1}H-^{13}C Heteronuclear Single Quantum Coherence (^{1}H-^{13}C-HSQC) and ^{1}H-^{13}C Heteronuclear Multiple Bond Correlation (^{1}H-^{13}C-HMBC) were used.

Fourier-Transformation Infrared (FT-IR) Spectroscopy

FT-IR spectra were recorded using a Bruker Alpha-P instrument applying ATR technology in a range of $\tilde{v}$ = 400-4000 cm^{-1} with 24 scans per measurement.

High-Resolution Mass Spectrometry (HRMS)

Mass spectra were recorded on a Q Exactive (Orbitrap) mass spectrometer (Thermo Fisher Scientific, San Jose, CA, USA) equipped with an atmospheric pressure ionization source operating in the nebulizer assisted electrospray mode. Calibration of the instrument was done in the m/z-range 150-2000 using a standard containing caffeine, Met-Arg-Phe-Ala acetate (MRFA) and a mixture of fluorinated phosphazenes (Ultramark 1621) (all from Sigma-Aldrich). A constant spray voltage of 3.5 kV, a dimensionless sheath gas of 6 and a sweep gas flow rate of 2 were applied. The capillary voltage and the S-lens RF level were set to 68.0 V and 320 °C, respectively.

Gas Chromatography - Flame Ioniziation Detector (GC-FID)

GC-FID screenings were conducted using a Bruker 430 GC instrument with a capillary column FactorFour™ VF-5ms (30 m × 0.25 mm × 0.25 mm) and a flame ionization detector (FID). The

measurements proceeded via standard measurement method: initial temperature 95 °C, heat to 200 °C with 15 °C·min^{-1}, hold 200 °C for 4 min, heat to 300 °C with 15 °C·min^{-1}and then hold 300 °C for 2 min. The sample preparation consisted of dissolving 1.5-3.0 mg of the compound in 1.5 mL of ethyl acetate. The mixture was filtered by syringe filter prior to use to avoid plugging of the injection setup or the column.

For determination of isomeric ratios, GC-FID measurements were performed using an Agilent 8860 gas chromatograph with a HP-5 column (30 m × 0.32 mm × 0.25 μm) and a flame ionization detector (FID). The measurements were carried out using the following heating program of the oven: initial temperature 95 °C, hold for 1 min, ramp up to 200 °C with a rate of 15 °C·min^{-1}, hold 200 °C for 4 min, ramp up to 300 °C with a rate of 15 °C·min^{-1} and then hold at 300 °C for 2 min. Measurements were performed in split-split mode using nitrogen as the carrier gas (flow rate 30 mL·min^{-1}) and were recorded for 20 min in total. For sample preparation, 1.5 mg of the substances were dissolved in 1.5 mL ethyl acetate and filtrated by syringe filter to avoid plugging of the injection setup or the column. The injection volume was set to 1 μL and the injection temperature to 220 °C.

Size Exclusion Chromatography (SEC)

For reaction monitoring of monomer synthesis, SEC measurements were performed in THF on a PSS SECcurity^2 GPC system based on Agilent infinity 1260 II hardware. The system was equipped with an autosampler SECcurity^2, a SECcurity^2 isocratic pump, a column oven (Bio)SECcurity^2 column compartment TCC6500 and a refractive index detector SECcurity^2 RI. Analysis was performed using two PSS SDV analytical columns (3 μm, 300 × 8.0 mm^2, 1000 Å) with a PSS SDV analytical precolumn (3 μm, 50 × 8.0 mm^2). The flow rate for measurements was set to 1 mL·min^{-1}. The system was calibrated with narrow linear poly(methyl methacrylate) standards (Polymer Standards Service, PSS, Germany) ranging from 102 to 62200 Da.

Polymer samples were characterized on a Shimadzu SEC system equipped with a Shimadzu isocratic pump model LC-20AD, a Shimadzu refractive index detector (24 °C) model RID-20A, a Shimadzu autosampler model SIL-20A and a Varian column oven model 510 (50 °C). For separation, a three-column setup was used with one SDV 3 μm, 8 × 50 mm precolumn and two SDV 3 μm, 1000 Å, 3 × 300 mm columns supplied by PSS, Germany. THF stabilized with 250 ppm butylated hydroxytoluene (BHT, ≥99.9%) supplied by Sigma-Aldrich was used at a flow rate of 1.0 mL·min^{-1}. For sample preparation, the amount of sample corresponding to 1.5 mg of expected polymer was dissolved in 1.5 mL of the mobile phase and filtrated by syringe filter to avoid plugging of the injection setup or the column. For calibration, six

poly(methyl methacrylate) standards (Agilent) ranging from 1102 Da to 62200 Da were used. The peak around 20.15 min is a system peak and does not belong to any impurities. The values of M_n, M_w and $Đ$ were determined by integration of the peaks in LabSolution software based on the performed calibration.

SEC measurements of higher molecular weight polymers were performed on a Shimadzu SEC system equipped with a Shimadzu isocratic pump (LC-20AD), a Shimadzu refractive index detector (30 °C) (RID-20A), a Shimadzu autosampler (SIL-20A) and a Shimadzu column oven (30 °C). The column system comprised a SDV 5 µm, 8 × 50 mm precolumn, a SDV 5 µm, 1,000 Å, 8 × 300 mm column and a SDV 5 µm, 100000 Å, 8 × 300 mm column supplied by PSS, Germany. A mixture of THF stabilized with 250 ppm butylated hydroxytoluene (BHT, ≥99.9%) and 2 vol.% triethylamine (≥99.5%) supplied by Sigma Aldrich was used at a flow rate of 1.00 mL·min^{-1}. For sample preparation, the amount of sample corresponding to 1.5 mg of expected polymer was dissolved in 1.5 mL of the mobile phase and filtrated by syringe filter to avoid plugging of the injection setup or the column. Calibration was carried out by injection of ten poly(methyl methacrylate) standards ranging from 1102 Da to 981000 Da. The values of M_n, M_w and $Đ$ were determined by integration of the peaks in LabSolution software based on the performed calibration.

Differential Scanning Calorimetry (DSC)

DSC measurements were performed on a Mettler Toledo DSC1 instrument equipped with a sample robot. Samples of 5-10 mg were loaded in a 100 µL aluminum crucible with pierced lid, and the measurements were performed under nitrogen atmosphere with a flow rate of 50 mL·min^{-1}. The DSC thermograms were recorded at a heating/cooling rate of 10 K·min^{-1} using the following heating/cooling program: first heating from 20 to 170 °C, then cooling from 105 to −70 °C, and a final heating step from −70 to 170 °C. The data from the last heating step are shown in the DSC curves.

2. Supplementary Note 1: Small-scale reaction monitoring

2.1 Carbonate formation

General procedure

To screen the optimum reaction conditions for the formation of limonene dicarbonate **7**, a stainless-steel reactor with Teflon inset was charged with 1.00 g limonene oxide (5.94 mmol, 1.00 equiv.), 6 mol% tetrabutylammonium halogenide and 30 bar CO_2 pressure. The reactor was heated to 120-180 °C and stirred for 20 h to 3 d. Afterwards, a GC-FID sample was taken to determine the constitution of the mixture.

Screening of reaction conditions

Supplementary Table 1. Variation of catalyst, reaction temperature and reaction time in the formation of carbonate 7. GC-FID fractions of **4**, **7a** and **7** are obtained by dividing the GC integral of the respective signal by the sum of the integrals of **4**, **7a** and **7**.

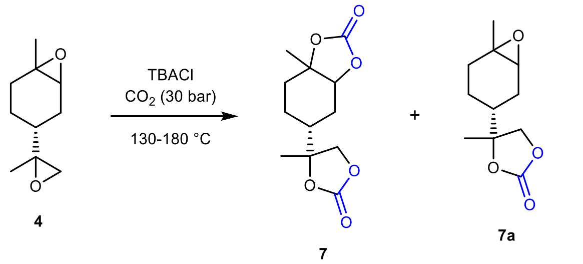

**Entry**	Catalyst	T / °C	t	**4:7a:7**
**1**	TBACl	100 °C	20 h	1:50:49
**2**	TBAB	100 °C	20 h	12:57:31
**3**	TBAI	100 °C	20 h	10:78:12
**4**	TBACl	130 °C	3 d	0:3:97

2.2 Carbonate opening reactions

General procedure

To monitor the reaction progress of carbonate opening over time, reactions were carried out in 1 mL vials. 2.5-5.0 mol% thiourea were added to 100 mg (1.0 equiv.) carbonate and

1.0-4.0 equiv. allylamine. The vial was sealed and the mixture was stirred at 60-80 °C. For reaction control, samples for GC-FID or SEC were taken directly out of the reaction mixture regularly.

Opening of carbonates 6 and 7

Supplementary Table 2. Conversion of limonene-derived carbonate 6 to urethane monomer 13. The respective GC-FID fractions of **13** and **6** is obtained by dividing the GC integral of the associated by the sum of the integrals of the signals assigned to **6** and **13**.

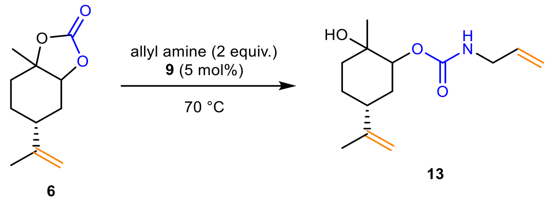

Entry	t / h	mol% 9	13:6
**1**	1	5	40:60
**2**	1	0	1:99
**3**	2	5	60:40
**4**	2	0	6:94
**5**	3	5	64:36
**6**	3	0	9:91
**7**	4	5	66:34
**8**	4	0	11:89
**9**	5	5	69:31
**10**	6	5	70:30
**11**	6	0	14:86
**12**	7	5	71:29
**13**	7	0	14:86
**14**	8	5	72:28
**15**	8	0	15:85

Supplementary Table 3. Conversion of limonene-derived carbonate 7 to urethane monomer 15. The SEC fractions of **15a** and **15** are obtained by dividing the SEC integral of the respective signal by the sum of the integrals of the signals assigned to **7**, **15a** and **15**.

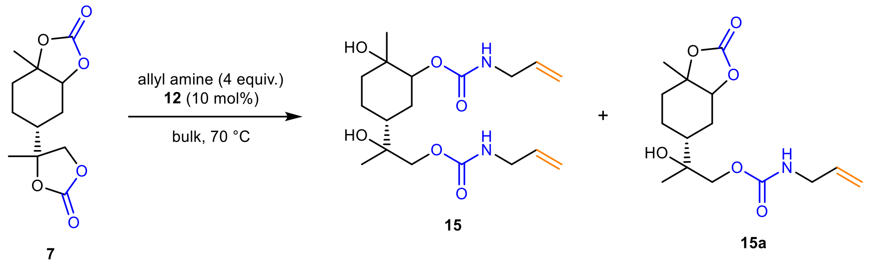

Entry	t / h	equiv. allylamine	15a:15
**1a**	2	4	36:64
**2a**	4	4	28:72
**3a**	6	4	25:75
**4a**	8	4	23:77
**5a**	10	4	23:77
**6a**	24	4	23:77
**7a**	48	4	25:75
**1b**	2	4	38:62
**2b**	4	4	28:72
**3b**	6	4	26:74
**4b**	8	5	23:73
**5b**	10	5	22:78
**6b**	24	5	20:80
**7b**	48	5	20:80

2.3 Thiol-ene polymerization reactions

General procedure

All thiol-ene polyaddition reactions were carried out in 1 mL glass vials. 100 mg of the urethane monomer (1.00 equiv.) and 1.00 equiv. of the dithiol were dissolved in the corresponding solvent (0.5 M) and 2.5-5.0 mol% DMPA were added. If 2-Me-THF was used as solvent, it was filtered over silica through a glass pipette prior to use. The reaction was stirred with a distance of 6 cm in front of a UV-lamp of 365 nm maximum wavelength. For reaction control, samples were taken out of the reaction mixture after a previously fixed and documented time. After 24 h, the solvent was evaporated. For precipitation, the polymer was dissolved in THF and poured into ice-cold methanol. After decantation, the precipitated polymer was dried under high vacuum (0.09-0.14 mbar) for 18 h.

Reaction progress over time

Supplementary Table 4. Thiol-ene polymerization of limonene-based urethane monomer 13 and 1,10-decanedithiol 19 over time. The average molecular weight M_n and the dispersity *Đ* were obtained from SEC measurements from the crude reaction mixture. Only one of two regioisomers of **13** is shown for clarity.

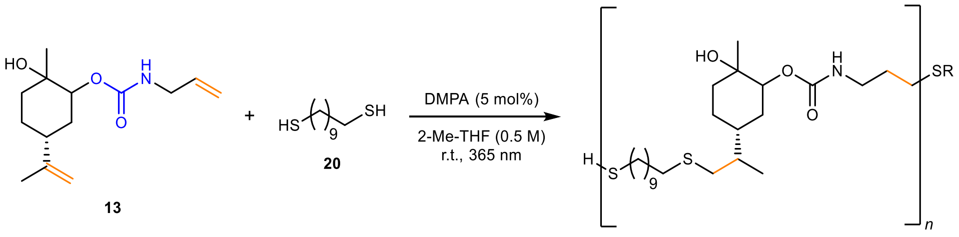

Entry	t / h	M_n / kDa	*Đ*
**1**	1	11.8	2.0
**2**	2	12.6	2.0
**3**	3	13.5	2.0
**4**	4	14.0	2.1
**5**	5	14.1	2.1

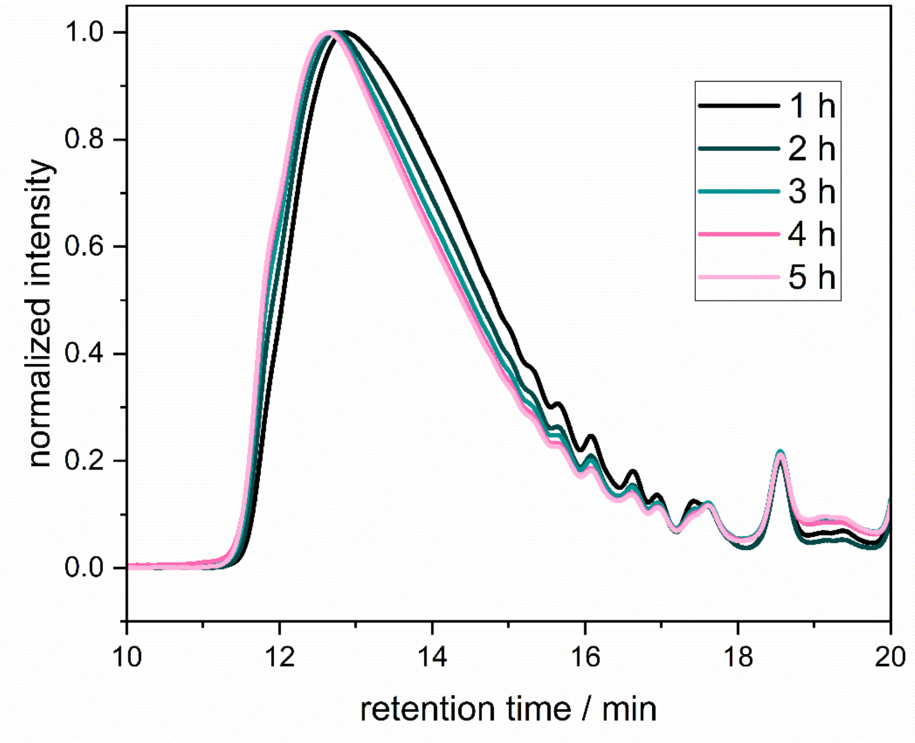

Supplementary Figure 1. Oligo-SEC screening over time regarding the polymerization of monomers 13 and 20 (see Supplementary Table 4).

Testing of reaction conditions

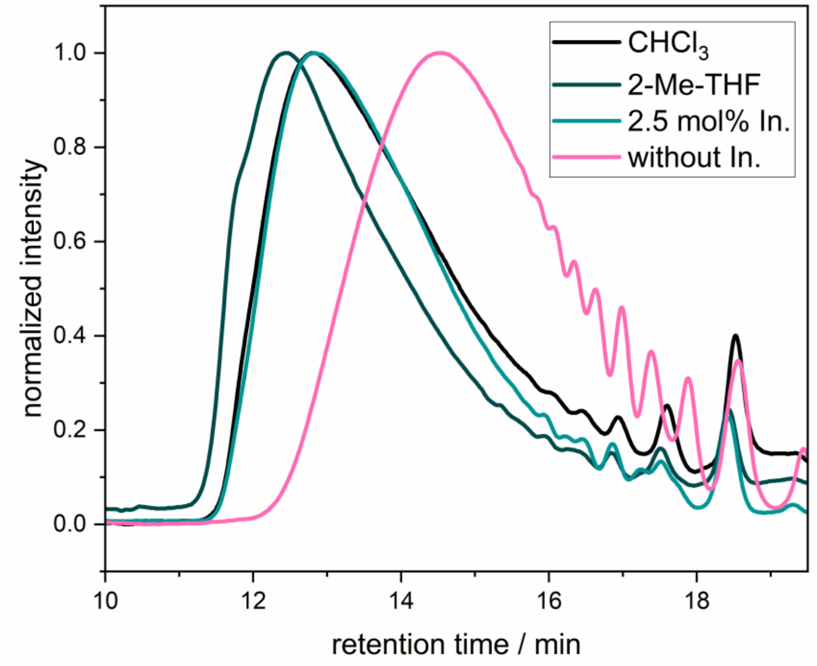

Supplementary Figure 2. Oligo-SEC measurements of crude polymerization tests (see Table 3). In. = 2,2'-dimethoxy-2-phenylacetophenone (DMPA).

3. Supplementary Note 2: Characterization of NIPUs

Supplementary Table 5. Linear NIPUs synthesized within this work. For precipitation, the evaporated crude mixture was dissolved in a minimum amount of THF and poured into 45 mL of anti-solvent, which was previously cooled to -20 °C.

Name	Diene	Dithiol	Precipitated in	Yield / %
**P1**	**13**	**20**	MeOH	64
**P2**	**13**	**21**	MeOH	26
**P3**	**13**	**22**	-	-
**P4**	**14**	**20**	-	-
**P5**	**15**	**20**	-	-
**P6**	**18**	**20**	MeOH	47
**P7**	**18**	**22**	-	-
**P8**	**19**	**20**	MeOH	40
**P9**	**19**	**22**	MeOH/H_2O 1:1	39

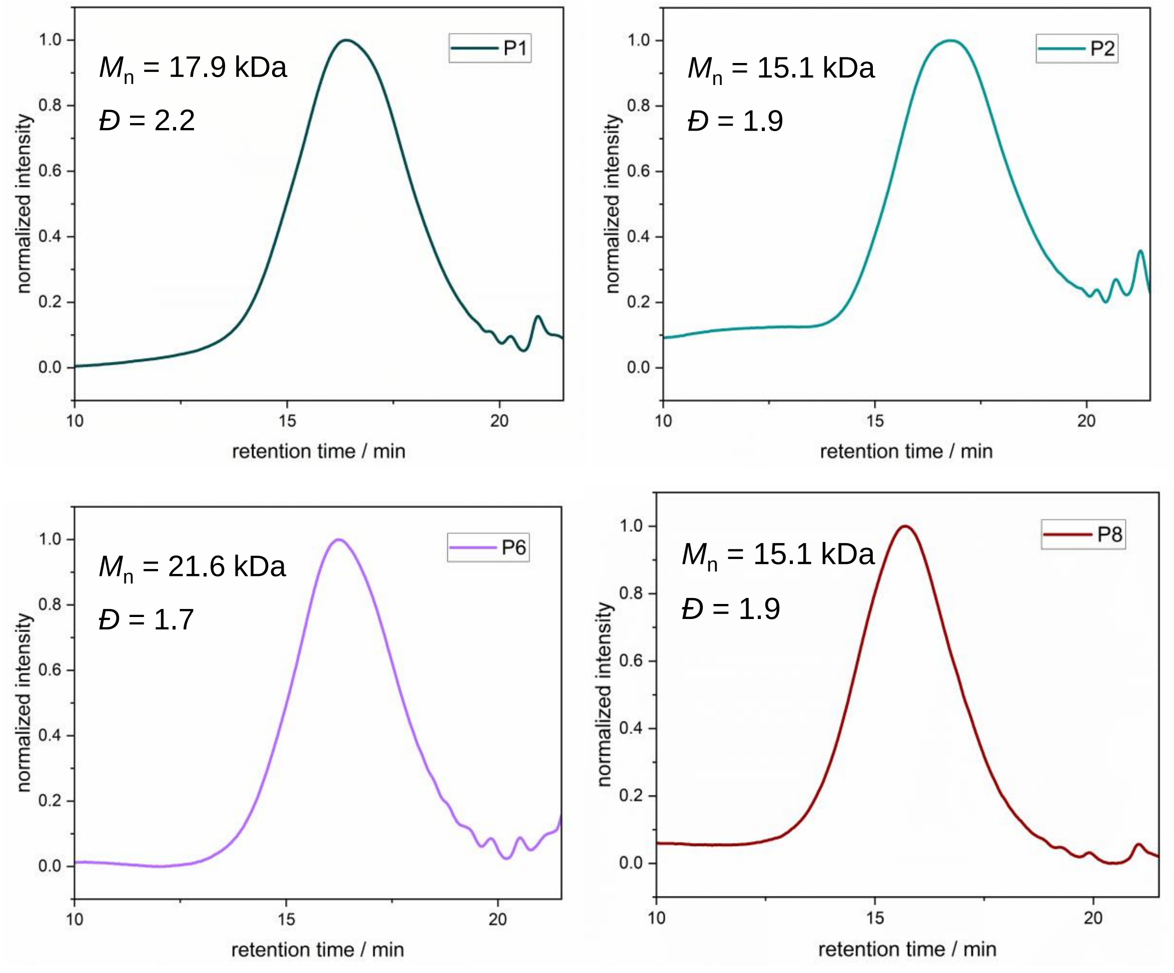

Supplementary Figure 3. Poly-SEC measurements of polymers P1, P2, P6 and P8.

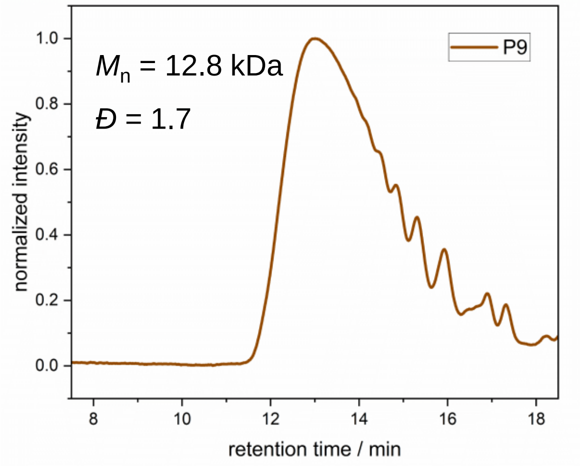

Supplementary Figure 4. Oligo-SEC measurement of polymer P9.

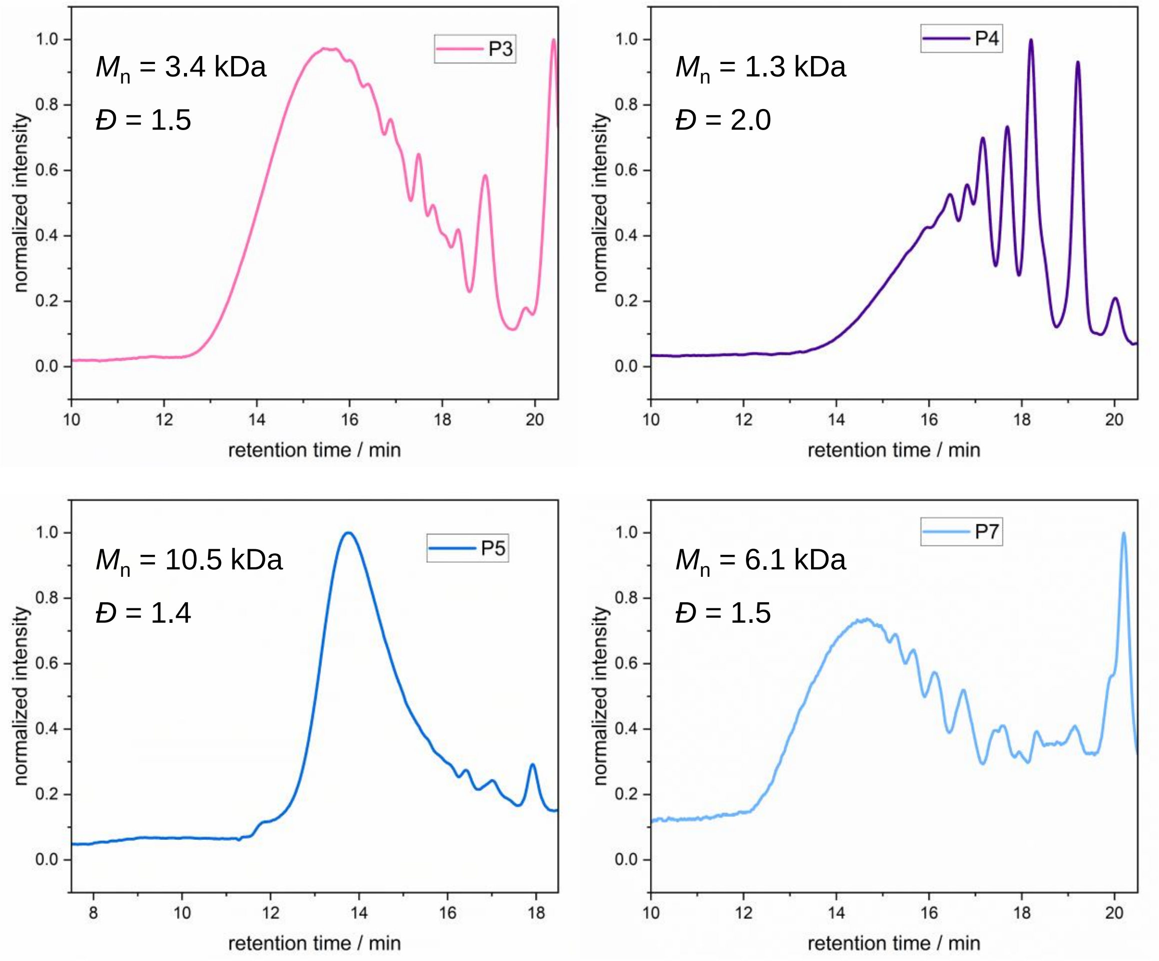

Supplementary Figure 5. Crude oligo-SEC measurements of P3, P4 and P7 that could not be precipitated in the anti-solvents tested within this work. The signal at 20.7 min is a system peak and does not correspond to any compound in the mixture.

3.1 Spectroscopic analysis of polymers

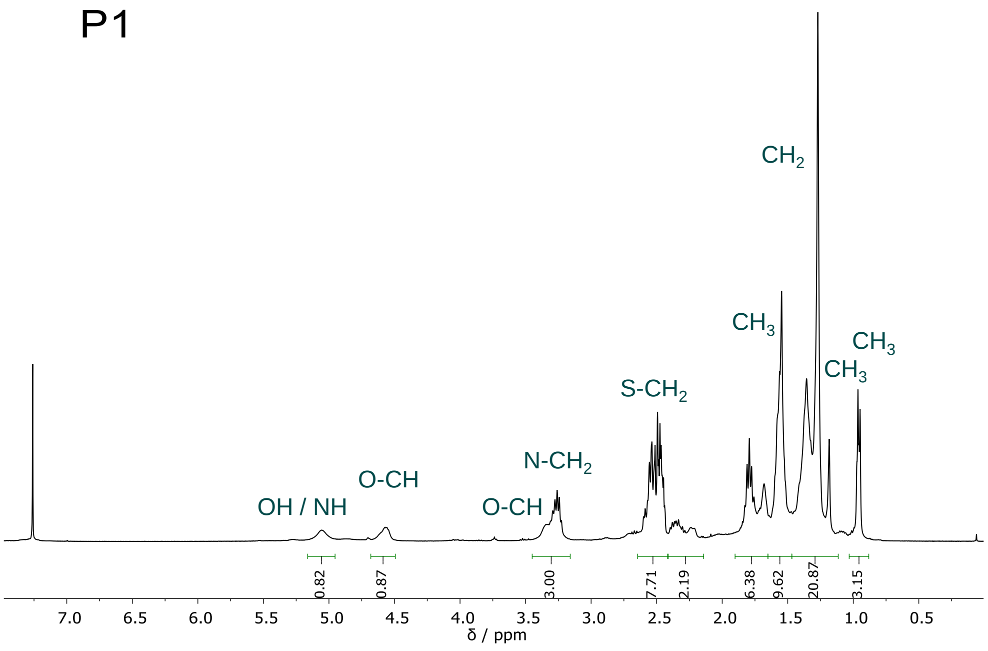

Supplementary Figure 6. ^1H NMR spectrum of P1, measured in $CDCl_3$.

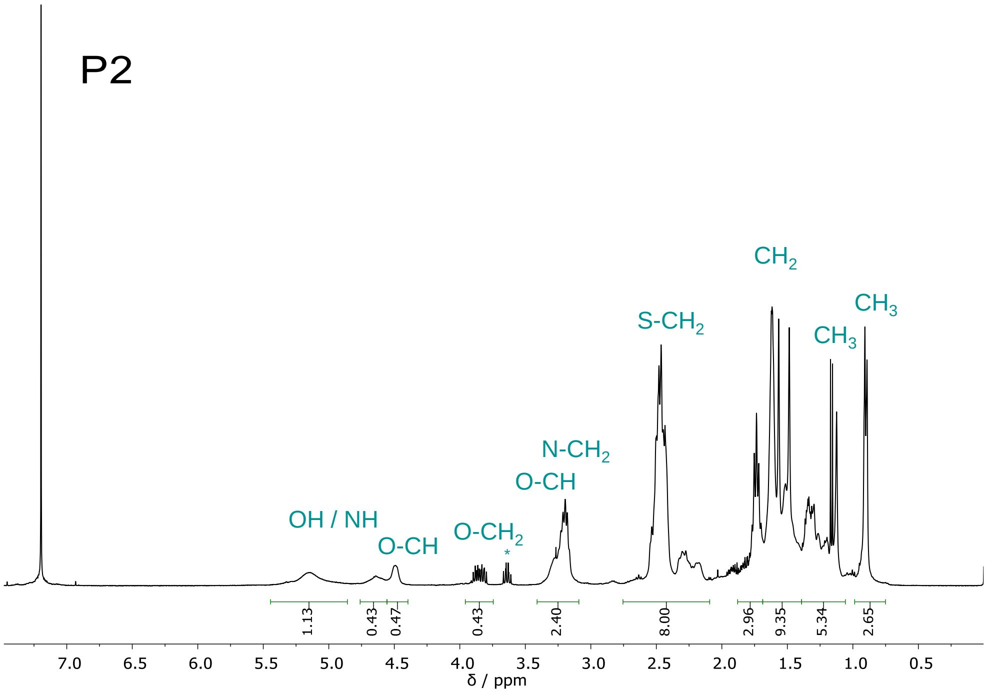

Supplementary Figure 7. ^1H NMR spectrum of P2, measured in $CDCl_3$.

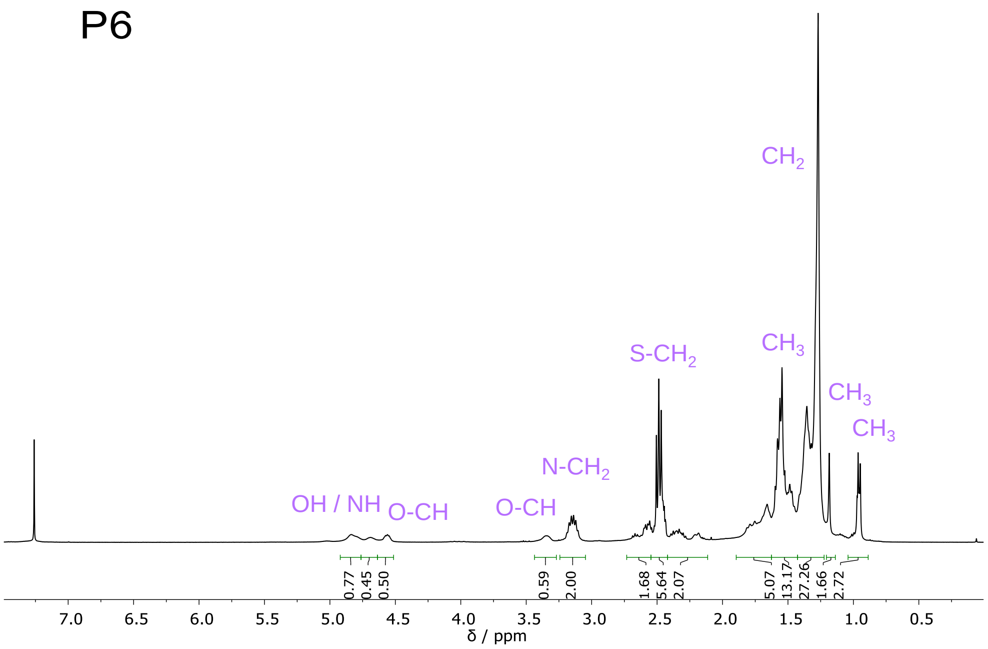

Supplementary Figure 8. ^{1}H NMR spectrum of P6, measured in $CDCl_3$.

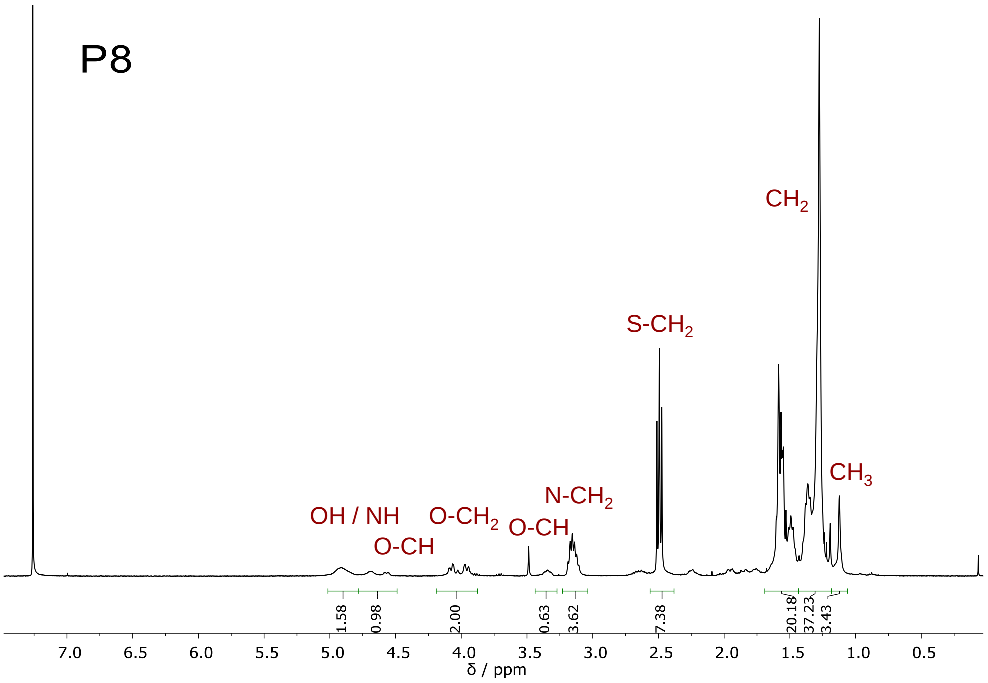

Supplementary Figure 9. ^{1}H NMR spectrum of P8, measured in $CDCl_3$.

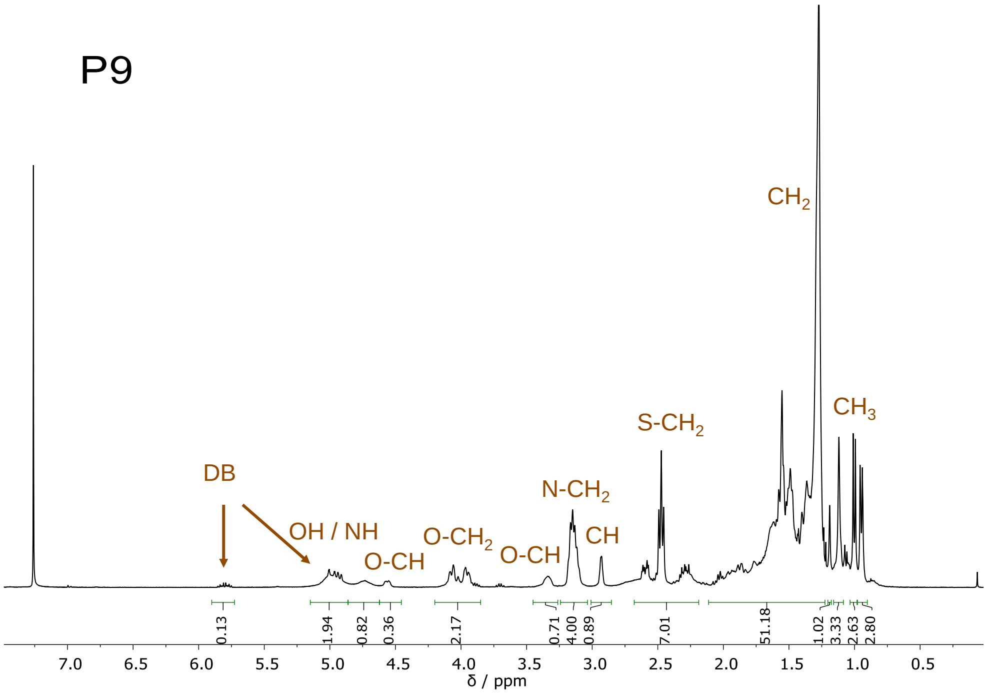

Supplementary Figure 10. ^{1}H NMR spectrum of P9, measured in CDCl$_3$.

Calculation of molecular weight:

$$M_n = n \cdot M(repeating\ unit) = \frac{\frac{I(NCH_2)}{4}}{\frac{I(DB)}{2}} \cdot M(repeating\ unit)$$

$$= \frac{4.00}{0.13 \cdot 2} \cdot (566.82 + 204.39) g \cdot mol^{-1} = 11\ 800\ g \cdot mol^{-1} \quad (1)$$

with n = number of repeating units,

I(NCH_2) = integral value of signal from 3.24 to 3.04 ppm,

I(DB) = integral value of signal from 5.90 to 5.73 ppm.

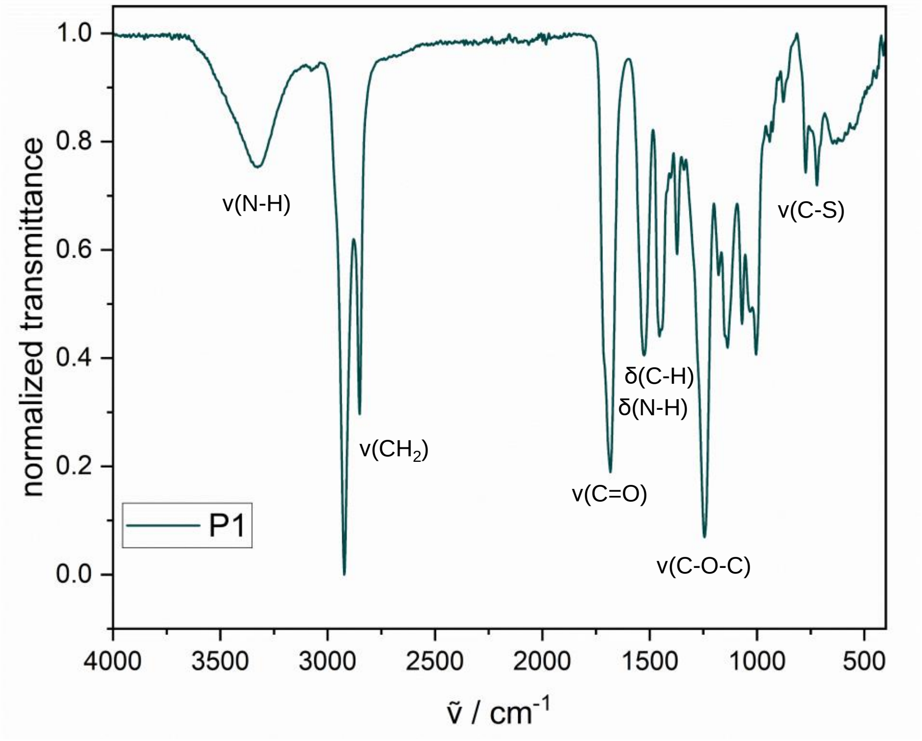

Supplementary Figure 11. IR spectrum of P1.

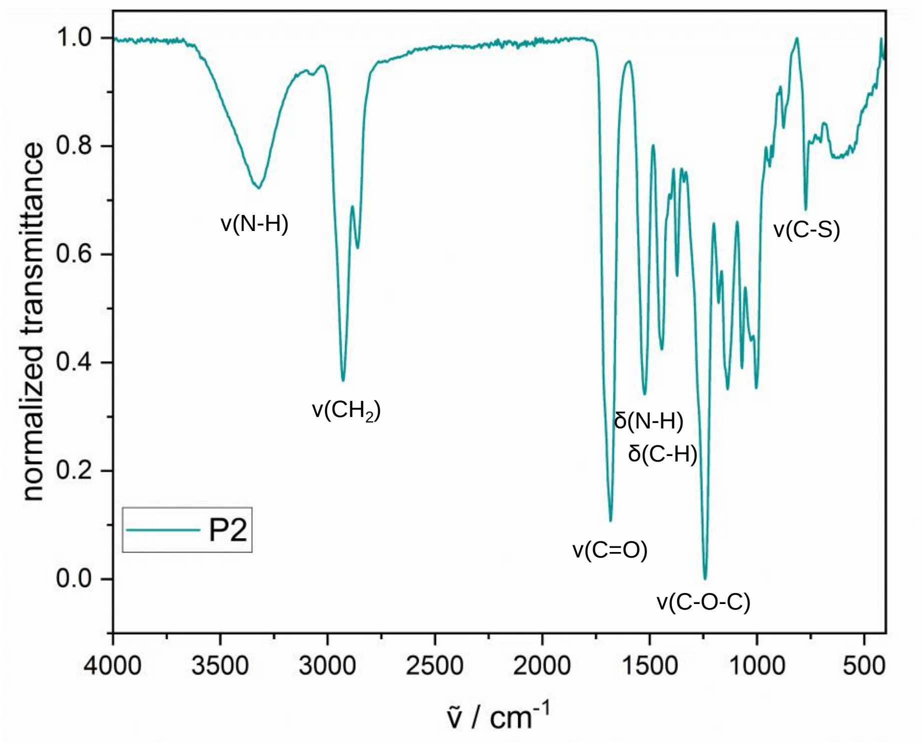

Supplementary Figure 12. IR spectrum of P2.

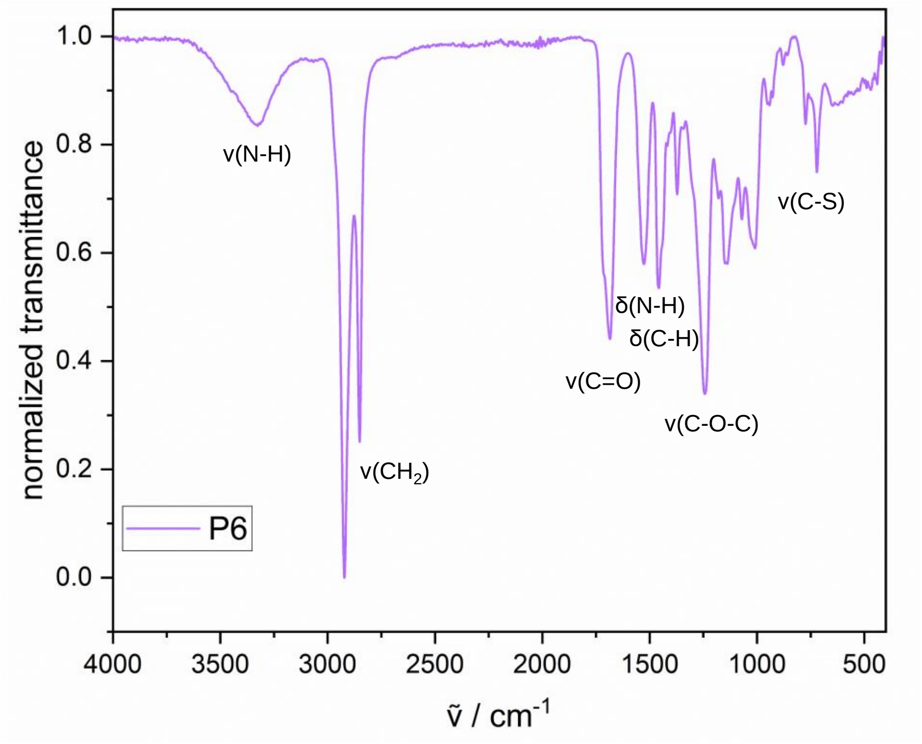

Supplementary Figure 13. IR spectrum of P6.

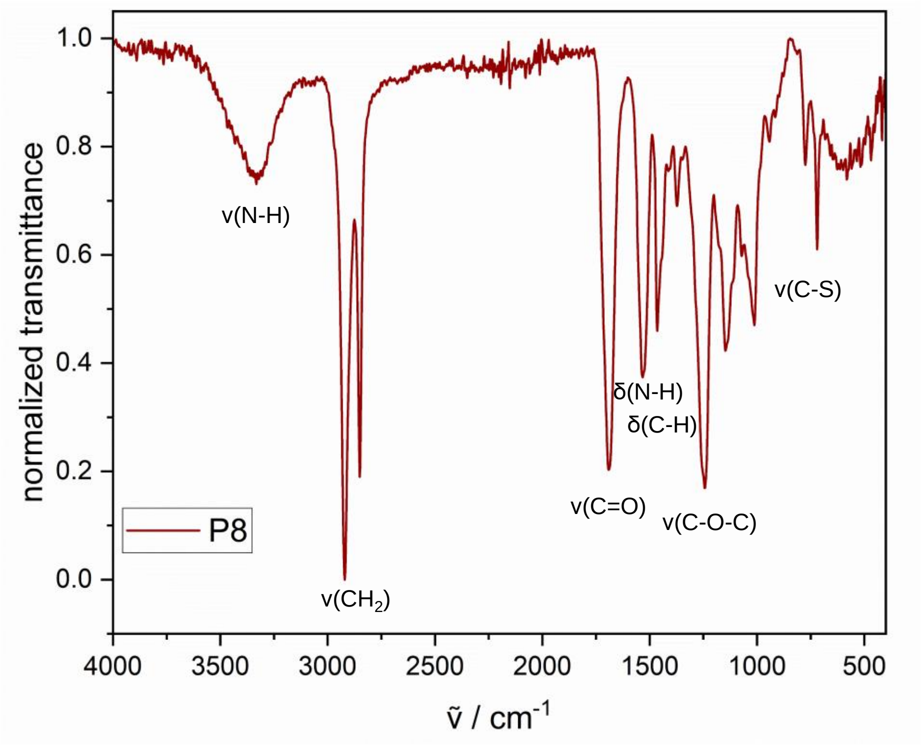

Supplementary Figure 14. IR spectrum of P8.

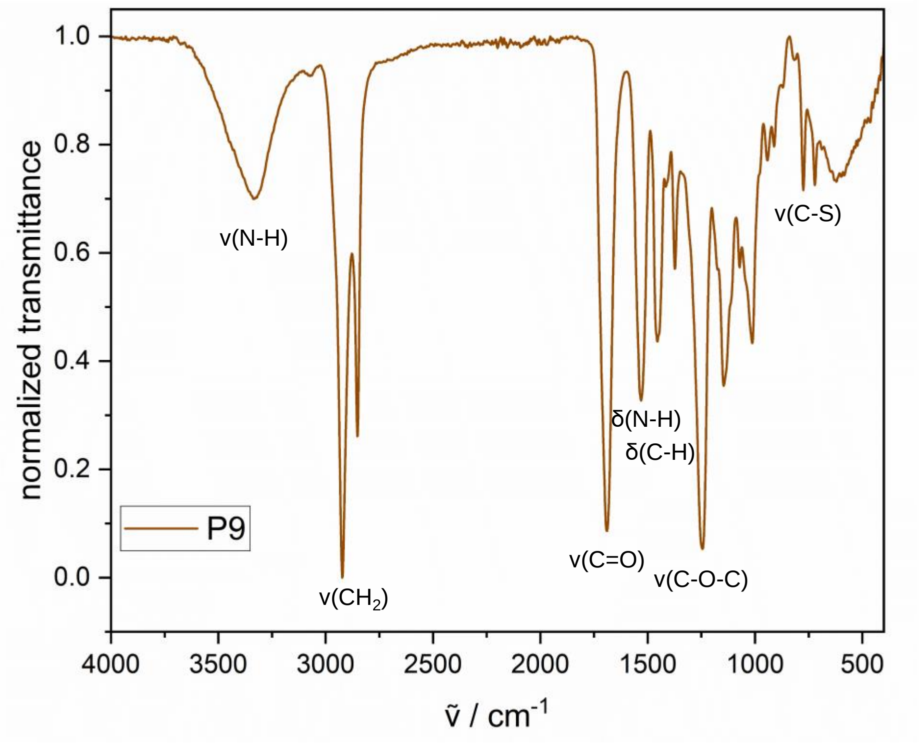

Supplementary Figure 15. IR spectrum of P9.

3.2 Thermal analysis of polymers

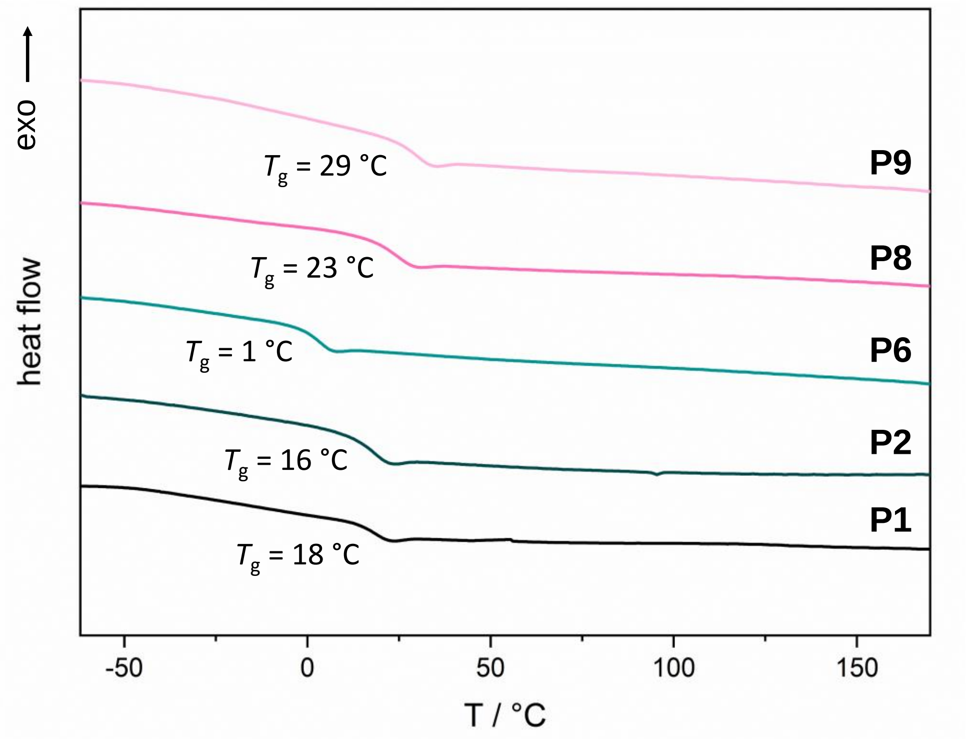

Supplementary Figure 16. DSC curves of precipitated polymers.

4. Supplementary Note 3: Monomer and catalyst syntheses

4.1 Synthesis of monomer 13

Limonene oxide 3

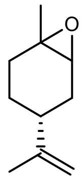

$C_{10}H_{16}O$
152.24 g/mol

The product was synthesized according to a literature-known procedure.[1]

In a three-necked round-bottom flask, 20.0 g (146 mmol, 1.00 equiv.) (*R*)-limonene were added to a solution of 20 mL water and 90 mL acetone and cooled to 0 °C. Then, 27.4 g (153 mmol, 1.05 equiv.) *N*-bromo succinimide were slowly added over a period of 30 min. After full conversion was detected via TLC, acetone was removed under reduced pressure. The mixture was diluted with 60 mL diethyl ether and the phases were separated. The organic phase was washed with 100 mL water and dried over Na_2SO_4. After removing the solvent, 40 mL aqueous sodium hydroxide (6 M) solution were added and the mixture was stirred at 60 °C for 90 min. After diluting with 60 mL diethyl ether, the phases were separated and the organic layer was washed with 30 mL saturated sodium bicarbonate solution and 30 mL water. The solvent was removed and the product was purified by column chromatography (cyclohexane/ethyl acetate 20:1). The product was obtained as a colorless liquid in a yield of 9.71 g (63.8 mmol, 49%).

R_f (cyclohexane/ethyl acetate 20:1) = 0.30, visualized by staining with Seebach solution.

^{1}H (400 MHz, $CDCl_3$) δ / ppm = 4.67-4.58 (m, 2H, **H_9**), 3.09-2.92 (m, 1H, **H_3**), 2.09-1.95 (m, 2H, **H_4**), 1.95-1.79 (m, 1H, **H_5**), 1.78-1.58 (m, 5H, **H_6**, **H_{10}**), 1.40-1.34 (m, 2H, **H_7**), 1.33-1.27 (s, 3H, **H_1**).

^{13}C (100 MHz, $CDCl_3$) δ / ppm = 149.3 (**C_8**) 149.1 (**C_8**), 109.2 (**C_9**), 60.7 (**C_3**) 59.4 (**C_3**), 57.6 (**C_2**), 57.5 (**C_2**), 40.9 (**C_5**), 36.3 (**C_5**), 30.9 (**C_4**), 30.0 (**C_6**), 28.7 (**C_6**), 26.0 (**C_7**), 24.5 (**C_7**), 23.2 (**C_1**), 21.2 (**C_{10}**), 20.3 (**C_{10}**).

IR (ATR platinum diamond): ṽ / cm^{-1} = 3073, 2969, 2931, 2861, 1741, 1645, 1449, 1433, 1378, 1360, 1312, 1252, 1209, 1182, 1119, 1097, 1038, 1024, 1013, 970, 885, 841, 806, 758, 671, 611, 556, 525, 507, 460, 444.

ESI-MS: $[M+H]^+$ calc. 153.1274, detected 153.1273.

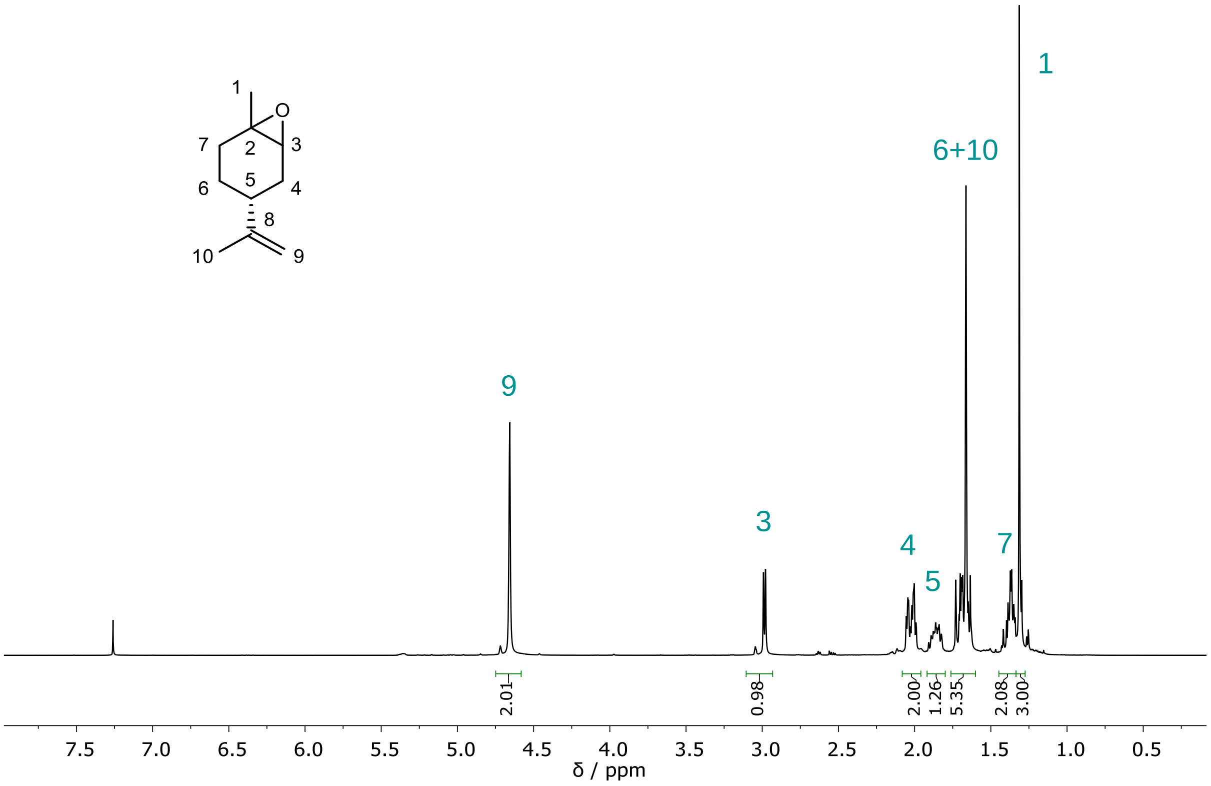

Supplementary Figure 17. ^{1}H NMR spectrum of 3, measured in $CDCl_3$.

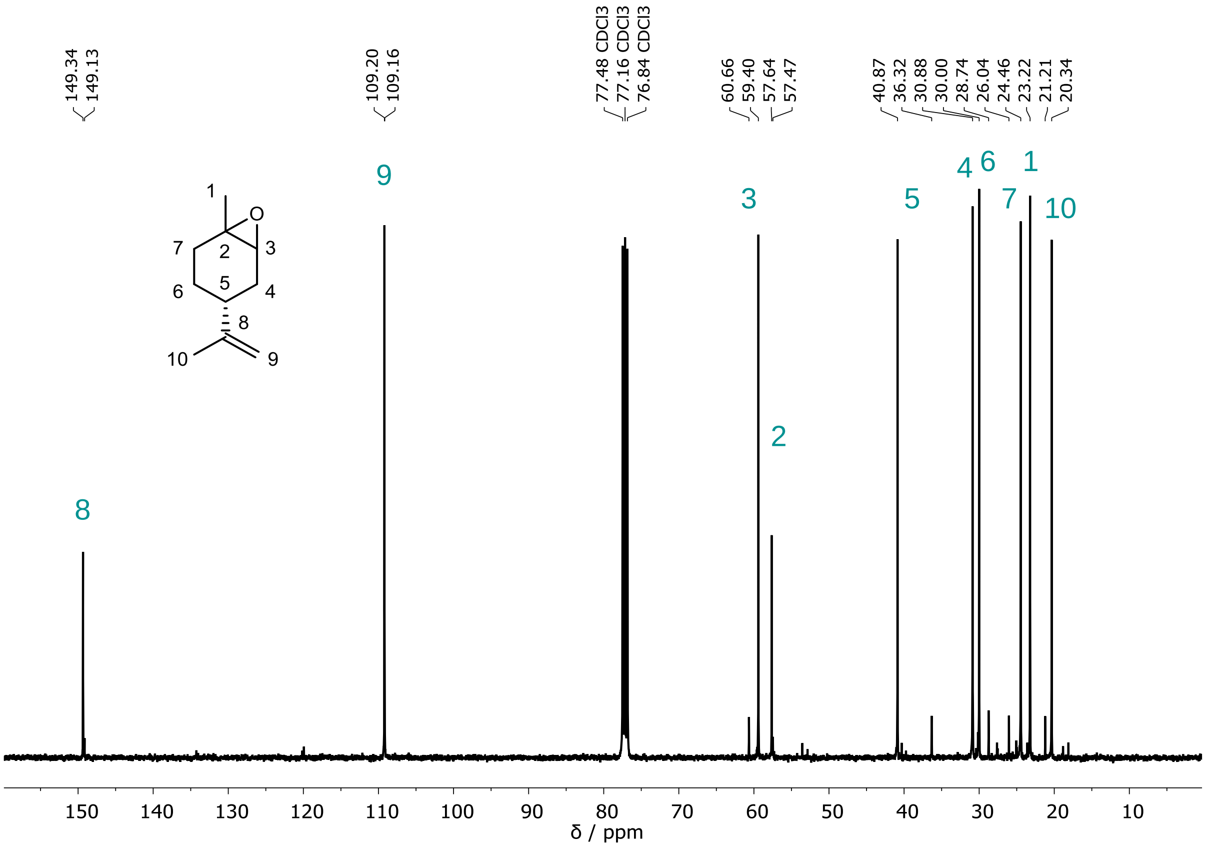

Supplementary Figure 18. ^{13}C NMR spectrum of 3, measured in $CDCl_3$.

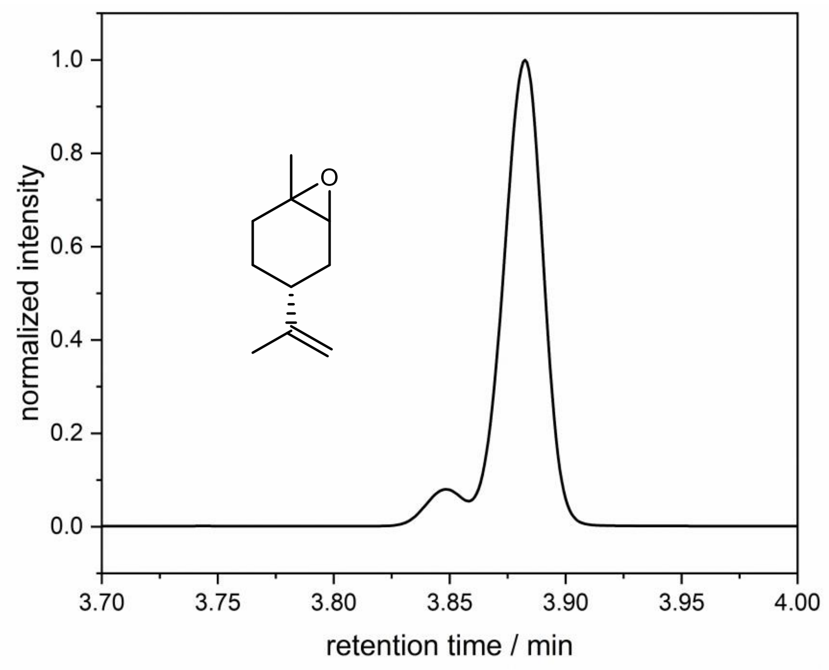

Supplementary Figure 19. Determination of diastereomeric ratio of compound 3 via GC-FID. The calculated diastereomeric ratio is 93:7.

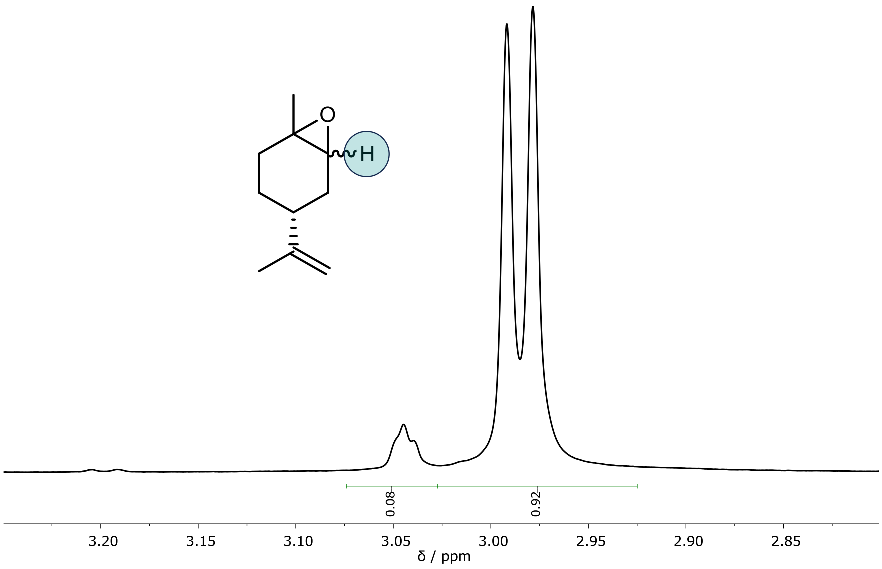

Supplementary Figure 20. Determination of diastereomeric ratio of compound 3 via NMR spectroscopy. The calculated diastereomeric ratio is 93:7.

Limonene monocarbonate 6

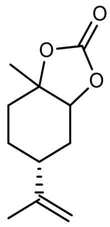

$C_{11}H_{16}O_3$
196.25 g/mol

A stainless-steel reactor with Teflon inset was charged with 8.00 g (52.6 mmol, 1.00 equiv.) limonene oxide **3**, 876 mg (3.15 mmol, 0.06 equiv.) TBACl and 30 bar CO_2 pressure. The reaction mixture was stirred and heated to 100 °C for 3 d. After completion of the reaction and cooling to room temperature, the viscous liquid was diluted with ethyl acetate (ca. 200 mL), washed with brine (3 × 100 mL) and extracted with ethyl acetate (50 mL). The combined organic phases were dried over Na_2SO_4 and the solvent was removed under reduced pressure. 8.23 g of almost pure limonene carbonate were obtained, as confirmed by ^{1}H NMR and GC-FID experiments, and was used directly for the synthesis of the urethane monomers **13** and **18**. For analysis, 1.00 g (12 wt%) of the crude reaction product was purified via column chromatography (cyclohexane/ethyl acetate 94:6 → 1:1) and 902 mg (4.56 mmol, corresponds to a yield of 72%) of the product were obtained as a colorless liquid.

R_f (cyclohexane/ethyl acetate 3:1) = 0.39, visualized by staining with Seebach solution.

^{1}H (400 MHz, $CDCl_3$) δ / ppm = 4.82-4.68 (m, 2H, **H_{11}**), 4.36 (dd, ^3J = 9.5, 7.0 Hz, 1H, **H_4**), 2.41-2.17 (m, 2H, **H_5+H_8**), 1.97-1.85 (m, 1H, **H_6**), 1.72 (s, 3H, **H_9**), 1.69-1.54 (m, 2H, **H_7+H_8**), 1.54-1.37 (m, 5H, **H_1+H_5+H_7**).

^{13}C (100 MHz, $CDCl_3$) δ / ppm = 154.9 (**C_2**), 147.4 (**C_{10}**), 110.3 (**C_{11}**), 82.2 (**C_3**), 80.7 (**C_4**), 40.0 (**C_6**), 34.1 (**C_5**), 33.2 (**C_8**), 26.3 (**C_1**), 25.81 (**C_7**), 20.7 (**C_9**).

IR (ATR platinum diamond): $\tilde{v}$ / cm^{-1} = 3082, 2938, 2868, 1789, 1646, 1561, 1455, 1442, 1382, 1348, 1293, 1271, 1247, 1214, 1188, 1150, 1127, 1075, 1024, 1010, 987, 941, 883, 817, 807, 780, 762, 724, 648, 615, 589, 559, 548, 523, 485, 454, 418.

ESI-MS: $[M+H]^+$ calc. 197.1172, detected 197.1171.

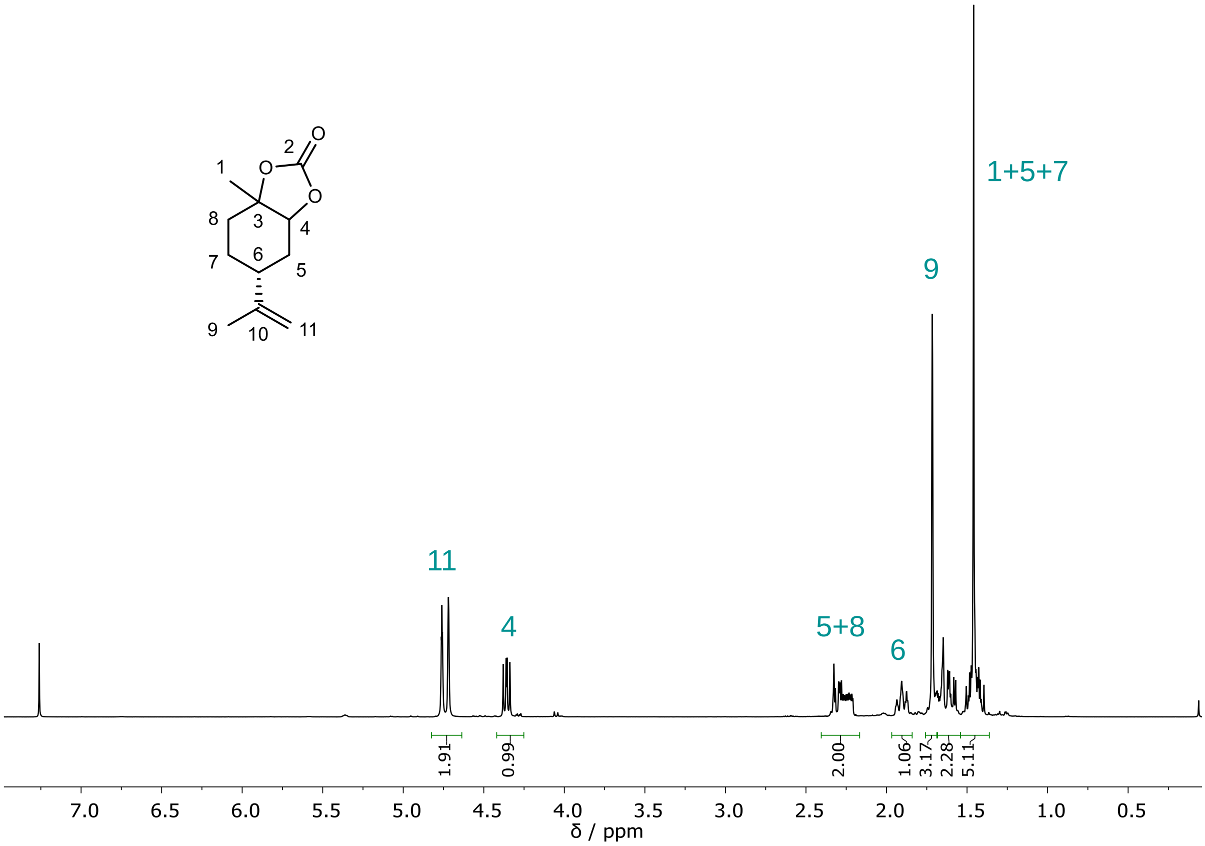

Supplementary Figure 21. ^{1}H NMR spectrum of 6, measured in $CDCl_3$.

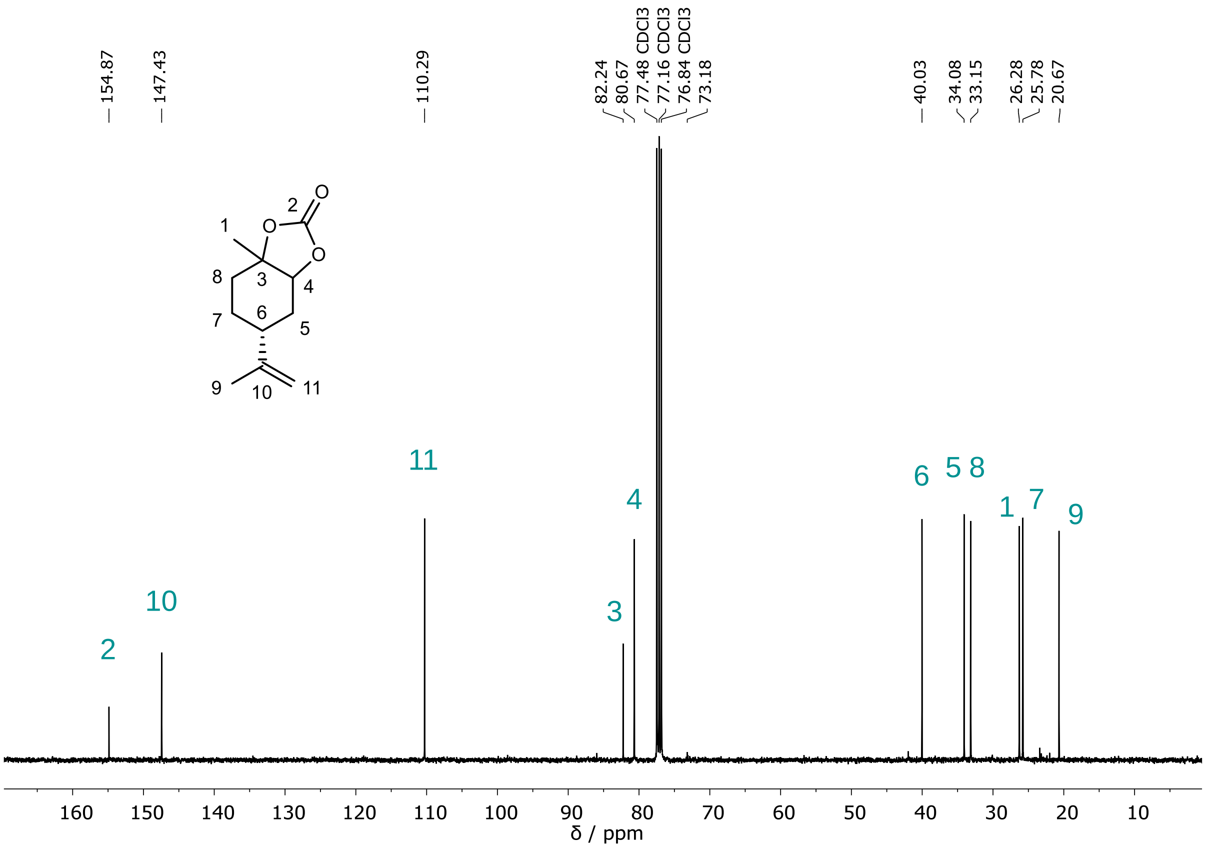

Supplementary Figure 22. ^{13}C NMR spectrum of 6, measured in $CDCl_3$.

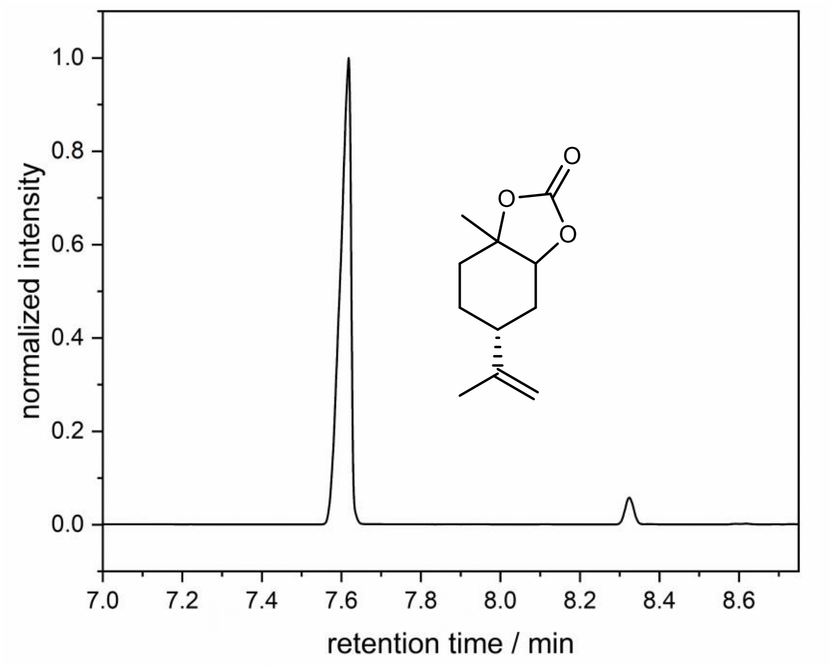

Supplementary Figure 23. Determination of diastereomeric ratio of compound 6 via GC-FID. The calculated diastereomeric ratio is 95:5.

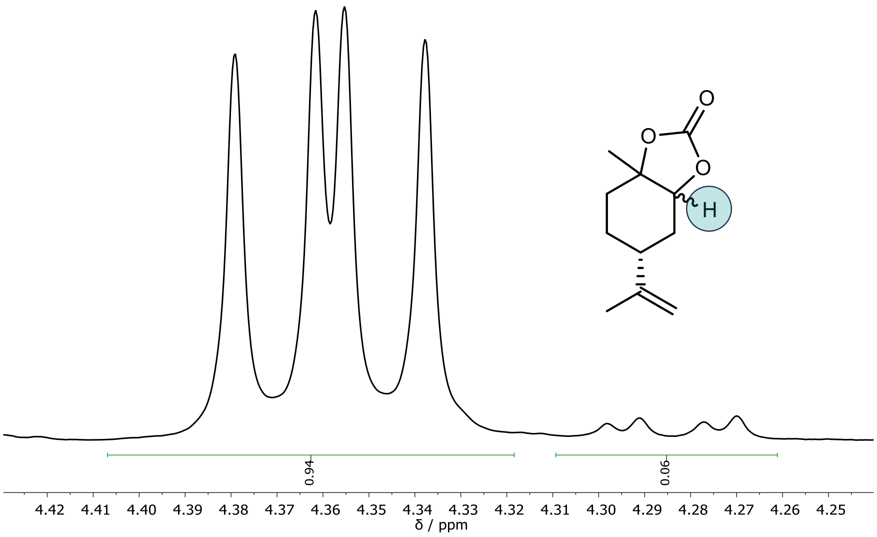

Supplementary Figure 24. Determination of diastereomeric ratio of compound 6 via NMR spectroscopy. The calculated diastereomeric ratio is 94:6.

Urethane monomer 13

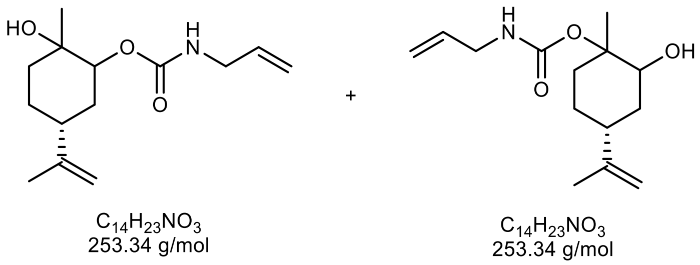

In a 5 mL pressure vial, 380 mg (1.0 mmol, 5.0 mol%) *N*-(3,5-bis(trifluormethyl)phenyl)-*N*'-cyclohexyl thiourea were added to 4.00 g (20.4 mmol, 1.00 equiv.) limonene carbonate **6** and 3.1 mL (2.3 g, 41 mmol, 2.0 equiv.) allylamine. The vial was sealed and the mixture was stirred at 70 °C overnight. After completion of the reaction (reaction control via TLC), the mixture was diluted with ethyl acetate, washed with brine (3 ×) and extracted with ethyl acetate. The combined organic phases were dried over Na_2SO_4 and the solvent was removed under reduced pressure. The crude product was purified via column chromatography (cyclohexane/ethyl acetate 3:1) and 4.39 g (17.3 mol, 85%) of the product were obtained as slightly yellow oil.

From GC-FID measurements, two isomers were observed in a ratio of 53:47.

R_f (cyclohexane/ethyl acetate 3:1) = 0.15, visualized by staining with Seebach solution.

^{1}H (400 MHz, $CDCl_3$) δ / ppm = 5.95-5.74 (m, **H_2**), 5.29-5.06 (m, **H_1**), 5.04-4.73 (m, **H_4**), 4.73-4.66 (m, **H_{16}**), 4.66-4.55 (m, **H_{8a}**), 4.54-4.39 (m, **H_{7b}**), 3.90-3.65 (m, **H_3**), 3.47-3.34 (m, **H_{9b}**) 2.36-2.21 (m, **H_{10b}**), 2.12-1.93 (m, **H_{7a}, H_{13}**), 1.92-1.74 (m, **H_{10a}, H_{12a}, H_{12b}**), 1.74-1.68 (m, **H_{15}**), 1.68-1.61 (m, **H_{12a}**), 1.61-1.56 (m, **H_{6b}**), 1.57-1.24 (m, **H_{10a}, H_{11}, H_{12b}**), 1.24-1.05 (m, **H_{6a}**).

Due to the occurrence of isomers, no integral values are given for the respective NMR signals. The sum of integrals matches the expected number of protons. The purity of the product was confirmed via GC-FID.

^{13}C (100 MHz, $CDCl_3$) δ / ppm = 156.5 (**C_5**), 155.9 (**C_5**), 148.9 (**C_{14}**), 148.7 (**C_{14}**), 134.4 (**C_2**), 134.3 (**C_2**), 116.2 (**C_1**), 116.1 (**C_1**), 109.1 (**C_{16}**), 108.8 (**C_{16}**), 83.9 (**C_{8b}**), 78.1 (**C_{8a}**), 76.1 (**C_{9b}**), 70.5 (**C_{9a}**), 43.9 (**C_{13}**), 43.5 (**C_3**), 37.4 (**C_{10a}**), 37.0 (**C_{10b}**), 36.1 (**C_{12b}**), 32.2 (**C_{12a}**), 27.2 (**C_{6a}**), 26.1 (**C_{11}**), 26.0 (**C_{11}**), 22.3 (**C_{6b}**), 21.0 (**C_{15}**), 20.8 (**C_{15}**).

IR (ATR platinum diamond): ṽ / cm^{-1} = 3330, 3083, 2935, 2863, 1683, 1645, 1525, 1453, 1394, 1375, 1246, 1186, 1141, 1071, 1008, 992, 917, 886, 851, 776, 655, 605, 582, 544, 515, 451.

ESI-MS: $[M+H]^+$ calc. 254.1751, detected 254.1747.

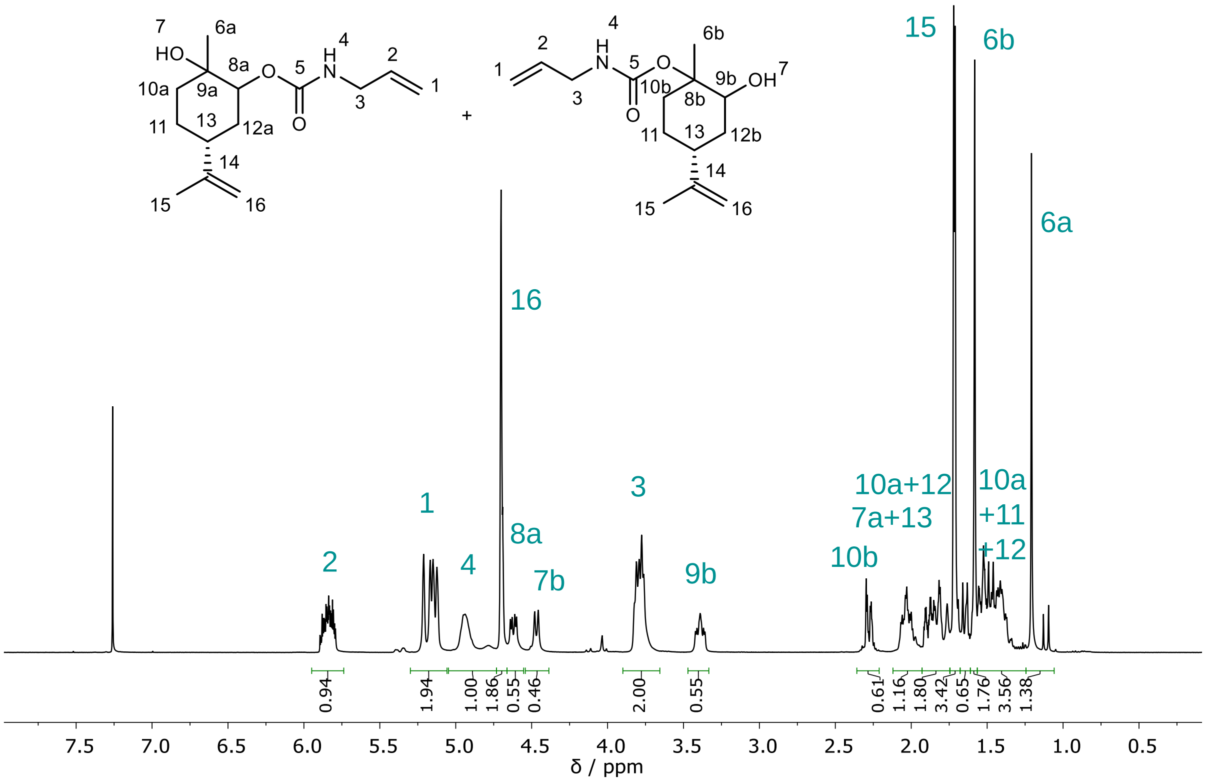

Supplementary Figure 25. ^{1}H NMR spectrum of 13, measured in $CDCl_3$.

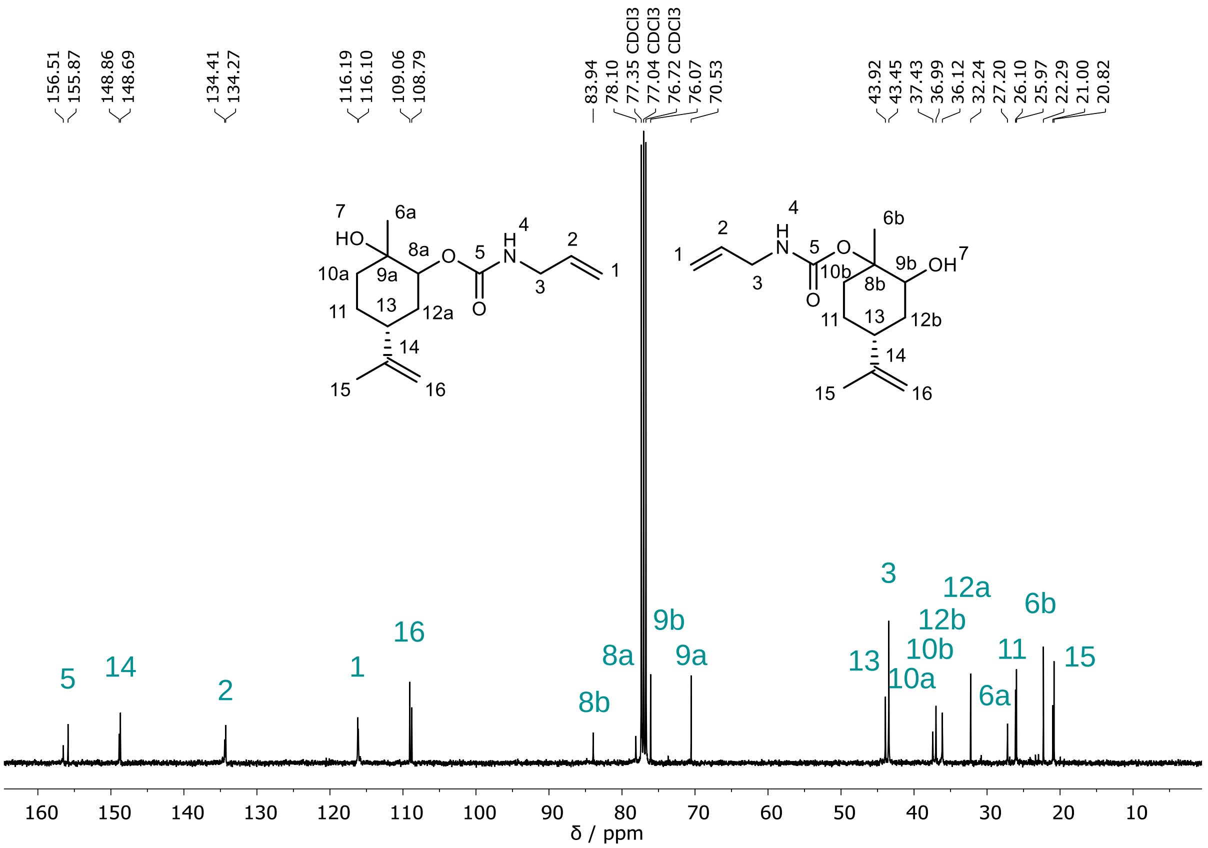

Supplementary Figure 25. ^{13}C NMR spectrum of 13, measured in $CDCl_3$.

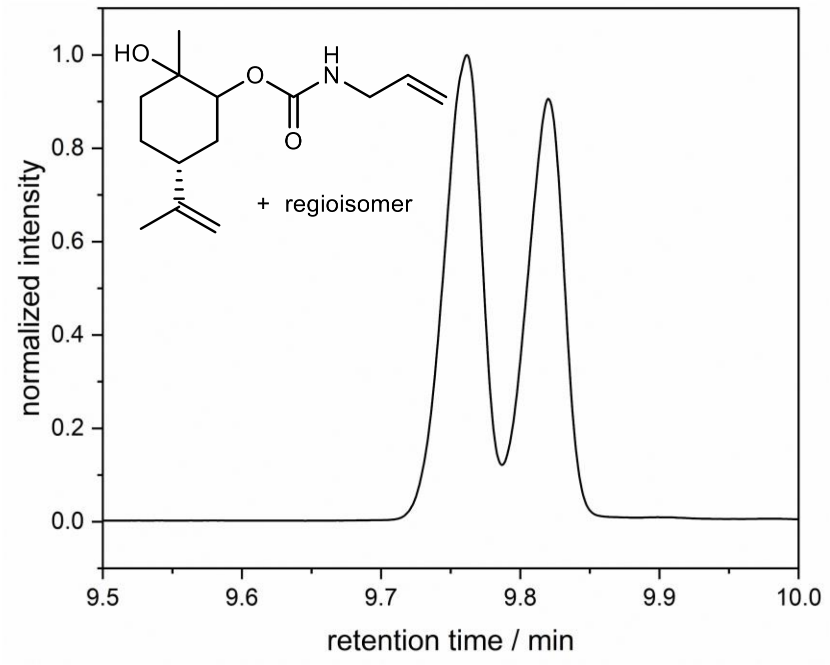

Supplementary Figure 26. Determination of regioisomeric ratio of compound 13 via GC-FID. The calculated regioisomeric ratio is 53:47.

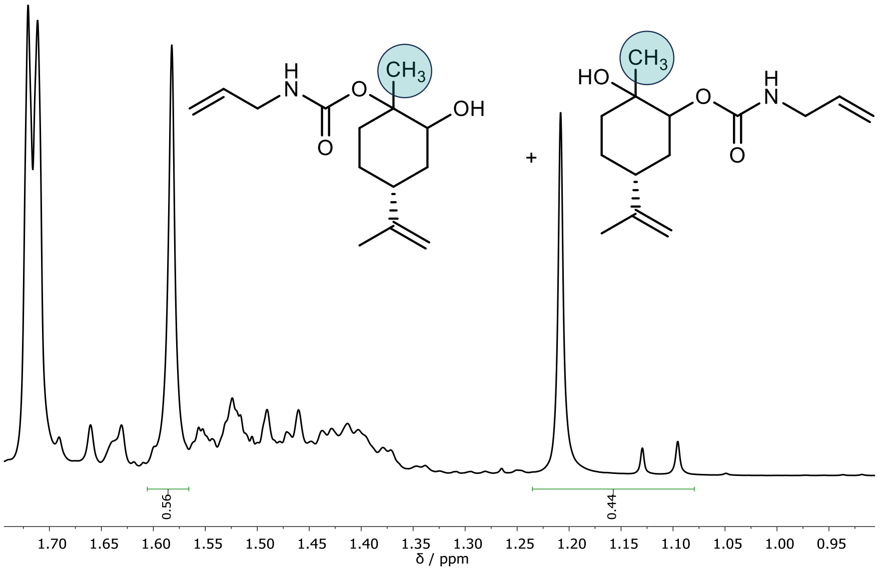

Supplementary Figure 27. Determination of regioisomeric ratio of compound 13 via NMR spectroscopy. The calculated regioisomeric ratio is 56:44.

4.2 Synthesis of monomer 15

Limonene dicarbonate 7

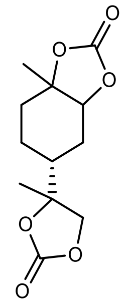

$C_{12}H_{16}O_6$
256.25 g/mol

A stainless-steel reactor with Teflon inset was charged with 10.0 g (59.4 mmol, 1.00 equiv.) limonene dioxide **4**, 991 mg (3.56 mmol, 0.06 equiv.) TBACl and 30 bar CO_2 pressure. The reaction mixture was stirred and heated to 130 °C for 3 d. After completion of the reaction and cooling to room temperature, the viscous liquid was purified via column chromatography (cyclohexane/ethyl acetate 5:1 → 1:1) and 13.8 g of a colorless liquid were obtained. The product was further recrystallized in cyclohexane/ ethyl acetate to yield 2.95 g (11.5 mmol, 19%) of the product as a colorless solid.

GC-FID showed the presence of two diastereomers that could not be separated sufficiently to determine their ratio.

$\mathbf{R_f}$ (cyclohexane/ethyl acetate 1:2) = 0.37, visualized by staining with Seebach solution.

^{1}H (400 MHz, $CDCl_3$) δ / ppm = 4.40-4.27 (m, $\mathbf{H_4}$), 4.27-4.15 (m, $\mathbf{H_{10}}$), 4.11-3.98 (m, $\mathbf{H_{10}}$), 2.35-2.23 (m, $\mathbf{H_5}$, $\mathbf{H_7}$), 2.12-2.01 (m, $\mathbf{H_5}$), 1.80-1.64 (m, $\mathbf{H_6}$, $\mathbf{H_8}$), 1.63-1.48 (m, $\mathbf{H_7}$, $\mathbf{H_8}$), 1.45-1.41 (m, $\mathbf{H_1}$), 1.41-1.37 (m, $\mathbf{H_{11}}$), 1.37-1.20 (m, $\mathbf{H_5}$, $\mathbf{H_8}$).

Due the occurrence of isomers, no integral values are given for the respective NMR signals. The sum of integrals matches the expected number of protons. The purity of the product was confirmed via GC-FID measurements.

^{13}C (100 MHz, $CDCl_3$) δ / ppm = 154.1 ($\mathbf{C_2}$/$\mathbf{C_{12}}$), 154.0 ($\mathbf{C_2}$/$\mathbf{C_{12}}$), 84.6 ($\mathbf{C_9}$), 81.9 ($\mathbf{C_3}$), 81.8 ($\mathbf{C_3}$), 79.6 ($\mathbf{C_4}$), 79.4 ($\mathbf{C_4}$), 73.1 ($\mathbf{C_{10}}$), 72.9 ($\mathbf{C_{10}}$), 40.7 ($\mathbf{C_6}$), 40.6 ($\mathbf{C_6}$), 32.5 ($\mathbf{C_7}$), 32.4 ($\mathbf{C_7}$), 29.0 ($\mathbf{C_5}$), 28.9 ($\mathbf{C_5}$), 26.0 ($\mathbf{C_1}$), 21.3 ($\mathbf{C_{11}}$), 20.9 ($\mathbf{C_{11}}$), 20.8 ($\mathbf{C_8}$), 20.7 ($\mathbf{C_8}$).

IR (ATR platinum diamond): $\tilde{\nu}$ / cm^{-1} = 2983, 2939, 2883, 2871, 1776, 1556, 1481, 1445, 1395, 1359, 1341, 1323, 1301, 1280, 1265, 1247, 1221, 1194, 1171, 1152, 1141, 1105, 1090, 1070, 1054, 1024, 942, 918, 897, 880, 842, 814, 773, 721, 703, 599, 579, 557, 524, 492, 476, 460, 425.

ESI-MS: $[M+H]^+$ calc. 257.1020, detected 257.1017.

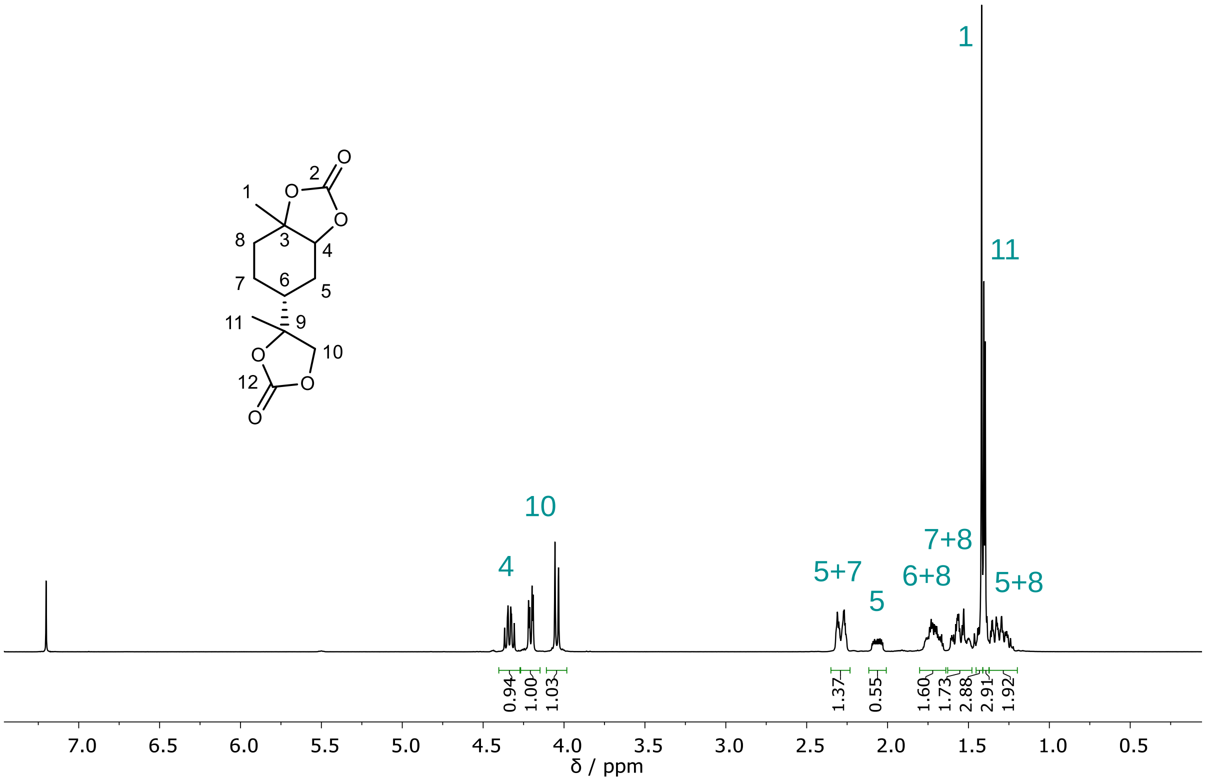

Supplementary Figure 28. **^{1}H NMR spectrum of 7, measured in $CDCl_3$.**

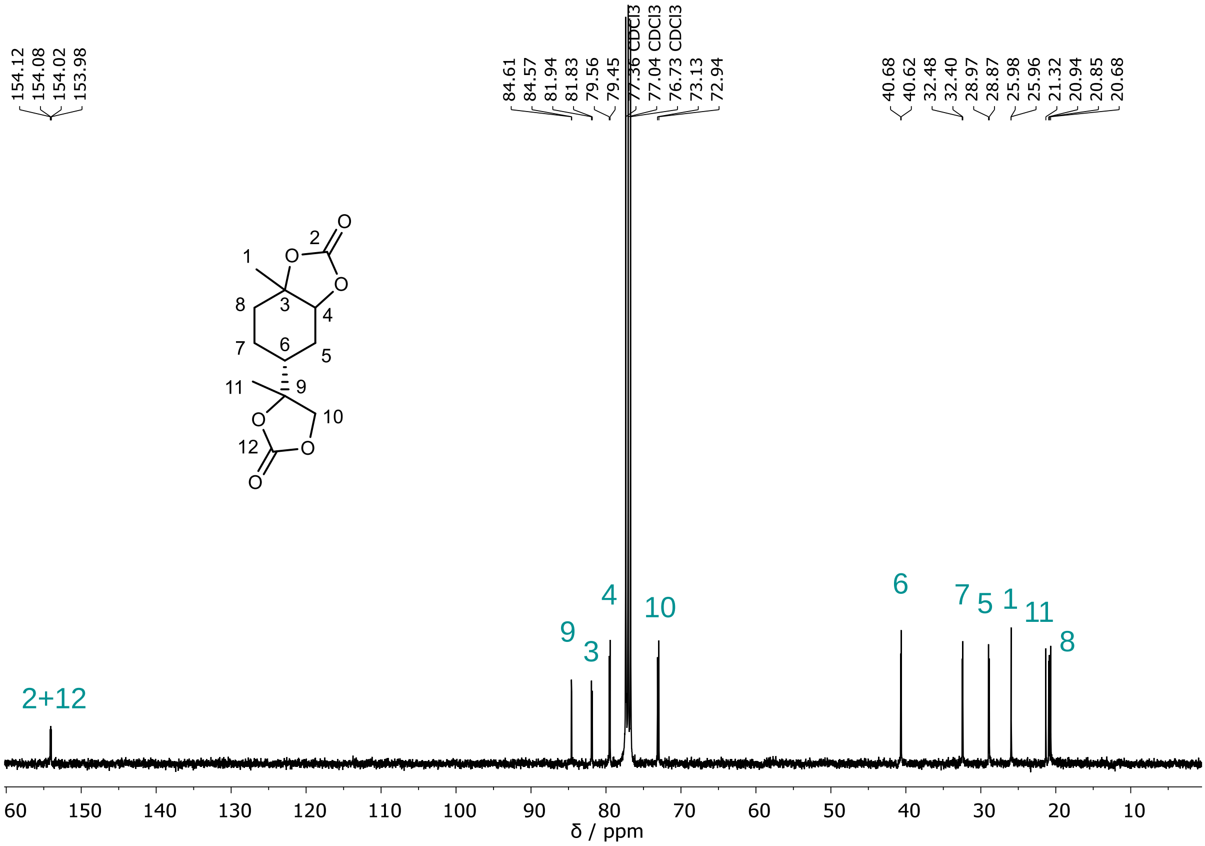

Supplementary Figure 29. **^{13}C NMR spectrum of 7, measured in $CDCl_3$.**

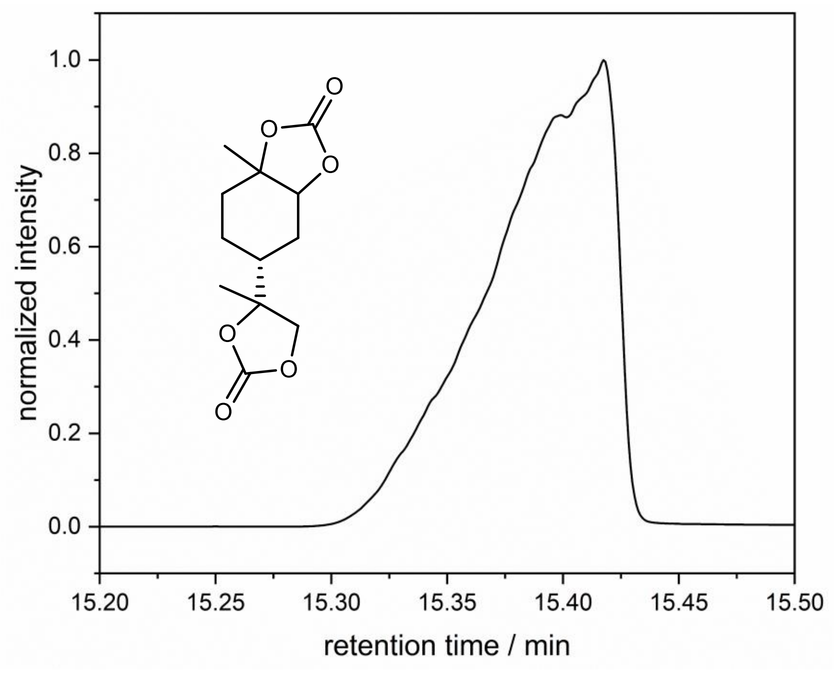

Supplementary Figure 30. Determination of diastereomeric ratio of compound 7 via GC-FID. The diastereomeric ratio could not be calculated due to insufficient separation of the signals.

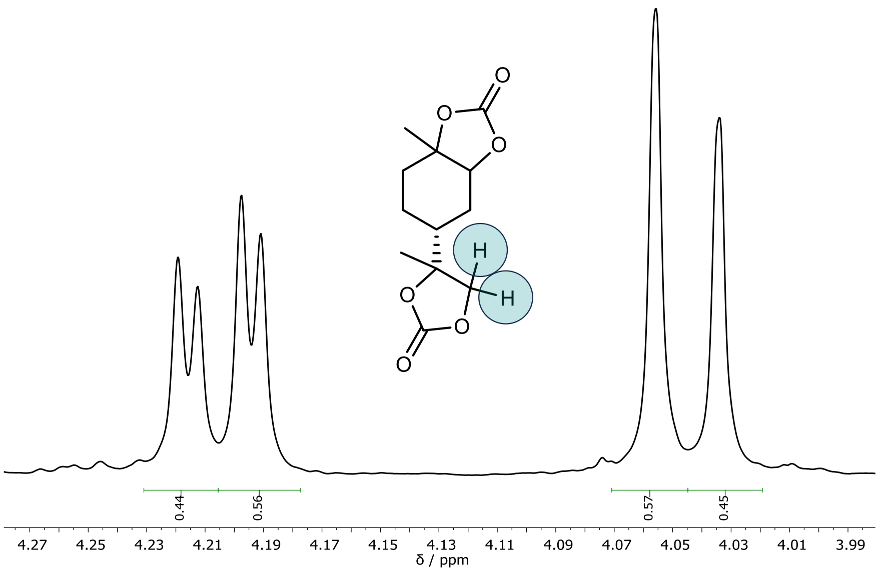

Supplementary Figure 31. Determination of diastereomeric ratio of compound 7 via NMR spectroscopy. The calculated diastereomeric ratio is 56:44.

Urethane monomer 15

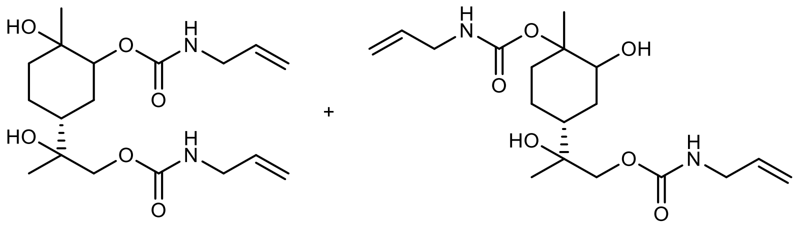

$C_{18}H_{30}N_2O_6$
370.45 g/mol

$C_{18}H_{30}N_2O_6$
370.45 g/mol

In a 5 mL pressure vial, 87 mg (0.23 mmol, 0.10 equiv.) *N*-(3,5-bis(trifluormethyl)phenyl)-*N'*-cyclohexyl thiourea **9** were added to 600 mg (2.34 mmol, 1.00 equiv.) limonene dicarbonate **7** and 1.06 mL (802 mg, 14.1 mmol, 6.00 equiv.) allylamine. The reaction was heated to 70 °C and stirred for 18 h. After complete conversion, the mixture was diluted in 100 mL ethyl acetate and washed with brine (3 ×). The aqueous phase was extracted with ethyl acetate (1 ×) and the combined organic phases were dried over Na_2SO_4. The solvent was evaporated and the crude product was purified via column chromatography (cyclohexane/ ethyl acetate 2:1 → 1:4) to yield 735 mg (1.99 mmol, 85%) of the product as slightly yellow, viscous liquid.

$\mathbf{R_f}$ (cyclohexane/ethyl acetate 1:1) = 0.15, visualized by staining with Seebach solution.

^{1}H (400 MHz, $CDCl_3$) δ / ppm = 5.95-5.75 (m, $\mathbf{H_{2}}$, $\mathbf{H_{21}}$), 5.34-4.82 (m, $\mathbf{H_{1}}$, $\mathbf{H_{4}}$, $\mathbf{H_{19}}$, $\mathbf{H_{22}}$), 4.78-4.64 (m, $\mathbf{H_{7b}}$), 4.63-4.50 (m, $\mathbf{H_{9a}}$), 4.18-3.93 (m, $\mathbf{H_{17}}$), 3.91-3.69 (m, $\mathbf{H_{3}}$, $\mathbf{H_{20}}$), 3.43-3.27 (m, $\mathbf{H_{9b}}$), 2.77-2.57 (m, $\mathbf{H_{14}}$), 2.34-2.19 (m, $\mathbf{H_{10\text{-}12}}$), 2.05-1.72 (m, $\mathbf{H_{10\text{-}12}}$), 1.71-1.22 (m, $\mathbf{H_{6b}}$, $\mathbf{H_{7a}}$, $\mathbf{H_{10\text{-}13}}$), 1.18 (s, $\mathbf{H_{6a}}$), 1.16-1.06 (m, $\mathbf{H_{15}}$).

Due to the occurrence of isomers, no integral values are given for the respective signals. The sum of integrals matches the expected number of protons. The purity of the product was confirmed via SEC measurements.

^{13}C (100 MHz, $CDCl_3$) δ / ppm = 160.0 ($\mathbf{C_{18}}$), 156.7 ($\mathbf{C_{5}}$), 156.0 ($\mathbf{C_{5}}$), 134.5 ($\mathbf{C_{2}/C_{21}}$), 134.4 ($\mathbf{C_{2}/C_{21}}$), 116.4 ($\mathbf{C_{1}/C_{22}}$), 116.2 ($\mathbf{C_{1}/C_{22}}$), 83.8 ($\mathbf{C_{8b}}$), 78.1 ($\mathbf{C_{9a}}$), 77.4 ($\mathbf{C_{8a}}$), 76.2 ($\mathbf{C_{9b}}$), 73.4 ($\mathbf{C_{16}}$), 70.7 ($\mathbf{C_{17}}$), 70.6 ($\mathbf{C_{17}}$), 43.9 ($\mathbf{C_{3}/C_{20}/C_{13}}$), 43.7 ($\mathbf{C_{3}/C_{20}/C_{13}}$), 43.6 ($\mathbf{C_{3}/C_{20}/C_{13}}$), 43.5 ($\mathbf{C_{3}/C_{20}/C_{13}}$), 43.3 ($\mathbf{C_{3}/C_{20}/C_{13}}$), 37.3 ($\mathbf{C_{10\text{-}12}}$), 36.9 ($\mathbf{C_{10\text{-}12}}$), 32.3 ($\mathbf{C_{10\text{-}12}}$), 28.5 ($\mathbf{C_{10\text{-}12}}$), 27.2 ($\mathbf{C_{6a}}$), 22.3 ($\mathbf{C_{6b}}$), 21.4 ($\mathbf{C_{15}/C_{10\text{-}12}}$), 21.2 ($\mathbf{C_{15}/C_{10\text{-}12}}$), 21.0 ($\mathbf{C_{15}/C_{10\text{-}12}}$), 20.8 ($\mathbf{C_{15}/C_{10\text{-}12}}$).

IR (ATR platinum diamond): ṽ / cm^{-1} = 3326, 3082, 2937, 1690, 1645, 1525, 1457, 1421, 1374, 1240, 1146, 1070, 1044, 1002, 916, 818, 776, 608, 547. (unvollständig)

ESI-MS: $[M+H]^+$ calc. 371.2177, detected 371.2174.

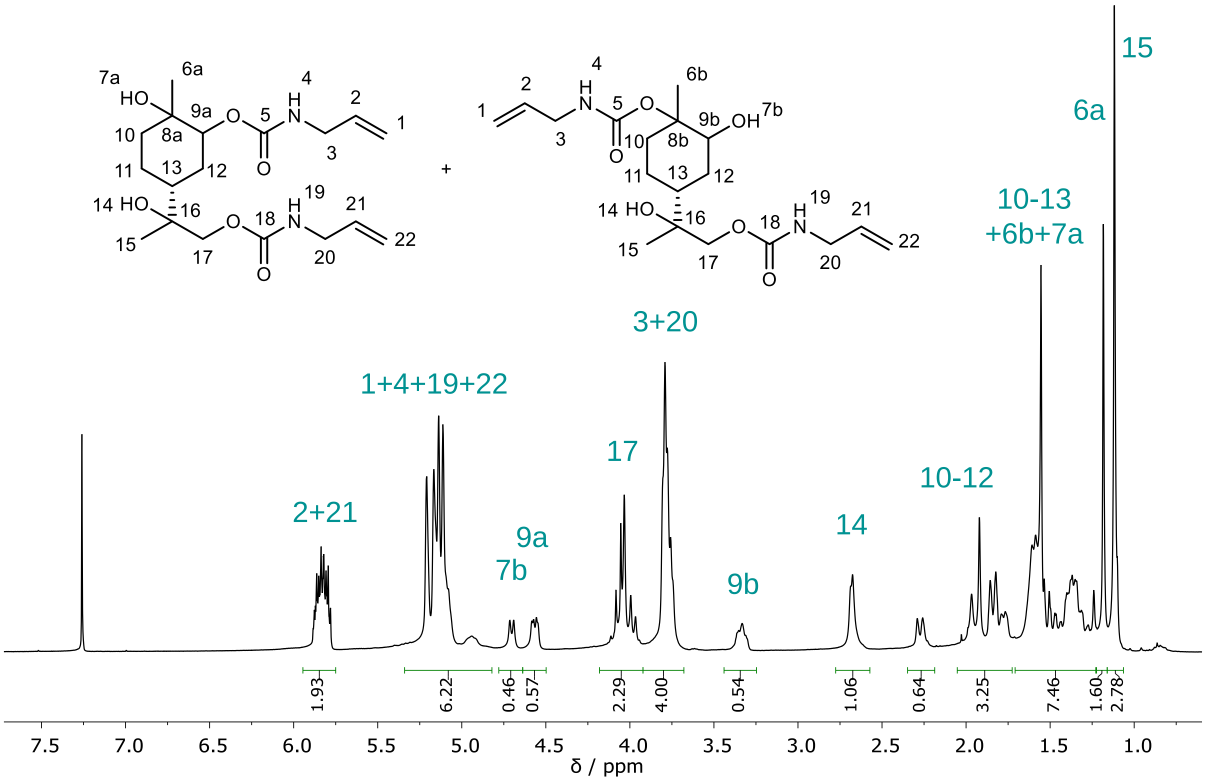

Supplementary Figure 32. ^{1}H NMR spectrum of 15, measured in $CDCl_3$.

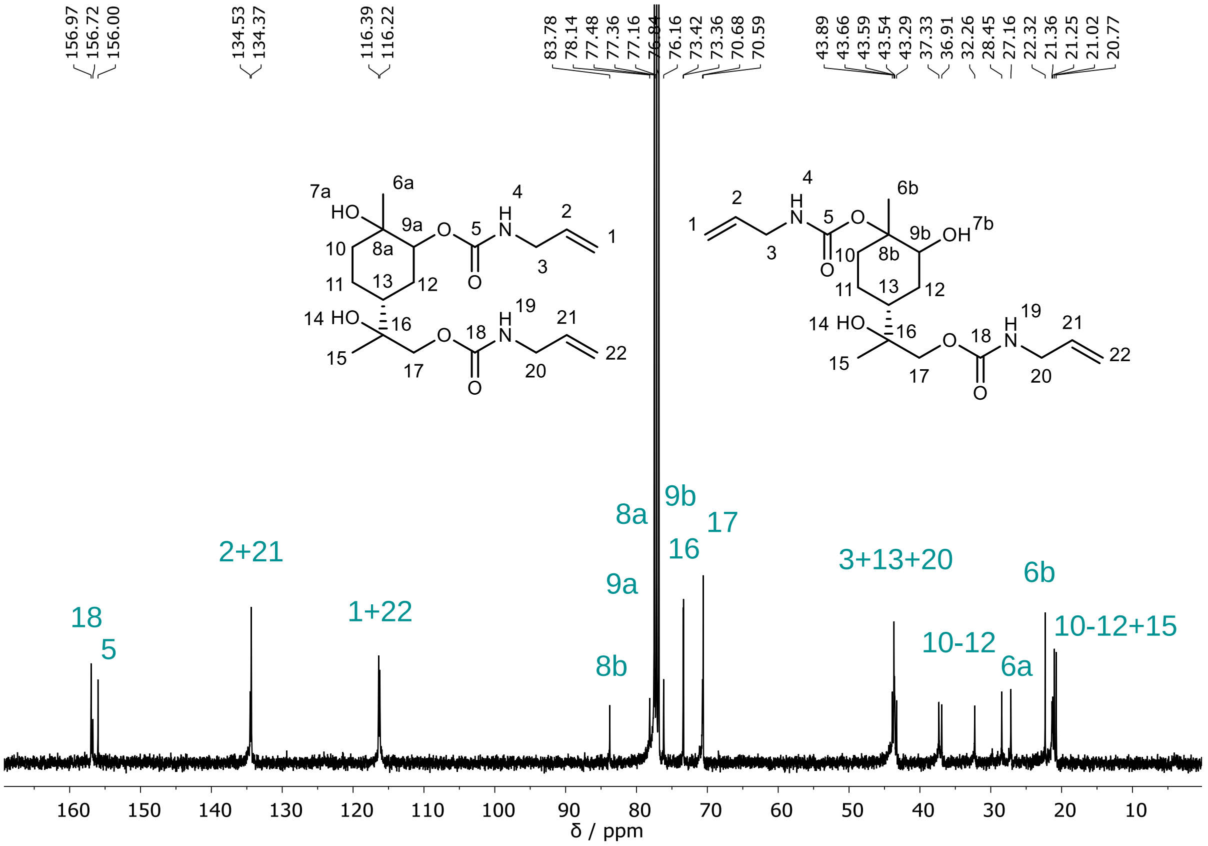

Supplementary Figure 33. ^{13}C NMR spectrum of 15, measured in $CDCl_3$.

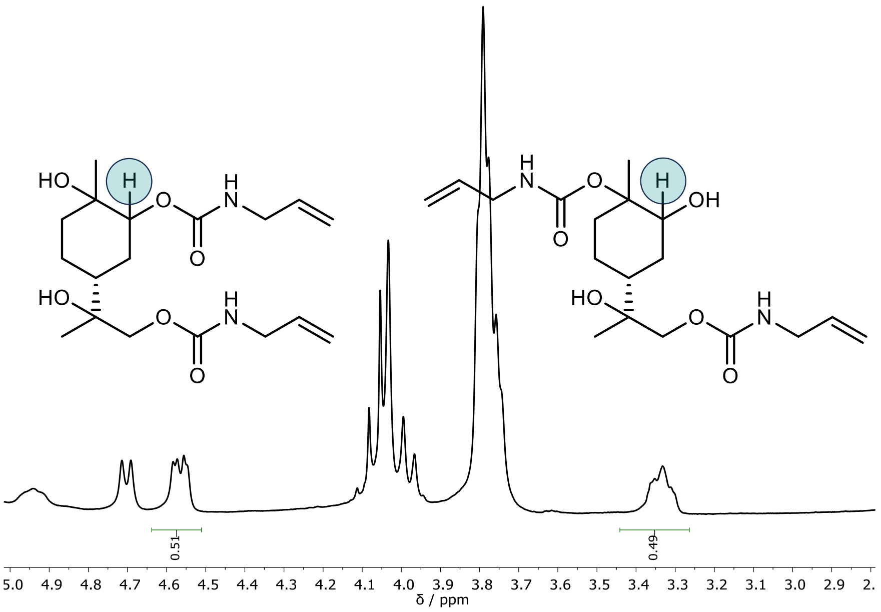

Supplementary Figure 34. Determination of regioisomeric ratio of compound 15 via NMR spectroscopy. The calculated regioisomeric ratio is 51:49.

As compound **15** was not sufficiently volatile to analyze via GC-FID, the constitution of regioisomers could only be determined based on NMR measurements.

4.3 Synthesis of monomer 14

Carvone oxide 5

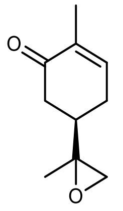

$C_{10}H_{14}O_2$
166.22 g/mol

5.00 g (33.3 mmol, 1.00 equiv.) (*S*)-carvone and 13.4 g (160 mmol, 4.80 equiv.) sodium bicarbonate were suspended in 100 mL acetone (0.33 M). 15.3 g (49.9 mmol, 1.50 equiv.) oxone®, dissolved in 100 mL water (0.5 M), were added slowly while stirring. The reaction mixture was stirred at room temperature for 45 minutes. Afterwards, the mixture was extracted with ethyl acetate (3 × 150 mL). The combined organic phases were washed with water (2 × 100 mL) and dried over Na_2SO_4. After removal of the solvent under reduced pressure, the mixture was purified via column chromatography (cyclohexane/ethyl acetate 4:1 → 3:1), yielding 4.69 g (28.2 mmol, 85%) of the product as slightly yellow oil.

R_f (cyclohexane/ethyl acetate 7:3) = 0.30, visualized by staining with Seebach solution.

^{1}H (400 MHz, $CDCl_3$) δ / ppm = 6.79-6.65 (m, 1H, **H_4**), 2.77-2.62 (m, 1H, **H_{10}**), 2.62-2.48 (m, 2H, **H_7**, **H_{10}**), 2.48-2.34 (m, 1H, **H_5**), 2.32-2.00 (m, 3H, **H_5**, **H_6**, **H_7**), 1.87-1.70 (m, 3H, **H_1**), 1.36-1.21 (m, 3H, **H_9**).

^{13}C (100 MHz, $CDCl_3$) δ / ppm = 198.9 (**C_2**), 144.2 (**C_4**), 144.0 (**C_4**), 135.8 (**C_3**), 135.7 (**C_3**), 58.1 (**C_8**), 58.0 (**C_8**), 53.0 (**C_{10}**), 52.5 (**C_{10}**), 41.5 (**C_6**), 40.8 (**C_6**), 40.5 (**C_7**), 40.1 (**C_7**), 28.0 (**C_5**), 27.8 (**C_5**), 19.1 (**C_9**), 18.5 (**C_9**), 15.8 (**C_1**).

IR (ATR platinum diamond): ṽ / cm^{-1} = 3038, 2975, 2924, 2891, 1710, 1667, 1486, 1450, 1435, 1365, 1249, 1205, 1144, 1106, 1051, 992, 957, 938, 903, 878, 830, 803, 763, 748, 704, 669, 625, 563, 524, 488, 426.

ESI-MS: $[M+H]^+$ calc. 167.1067, detected 167.1063.

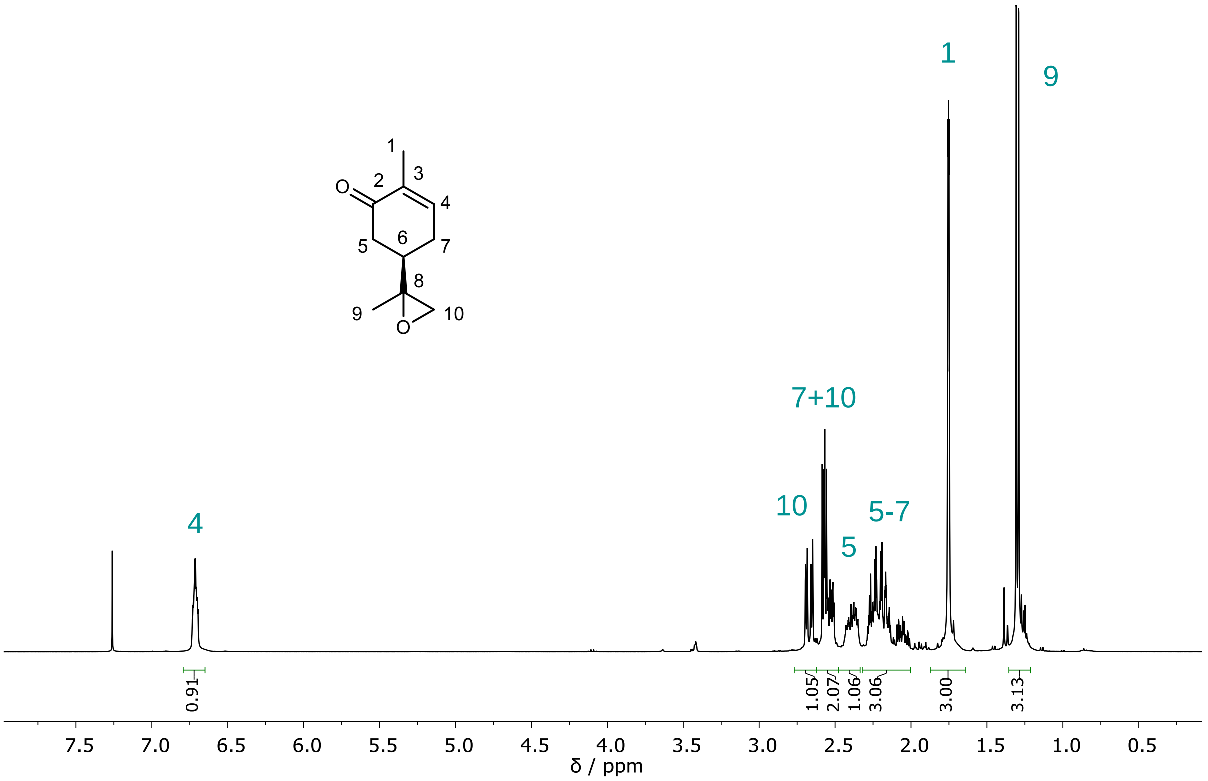

Supplementary Figure 35. **¹H NMR spectrum of 5, measured in $CDCl_3$.**

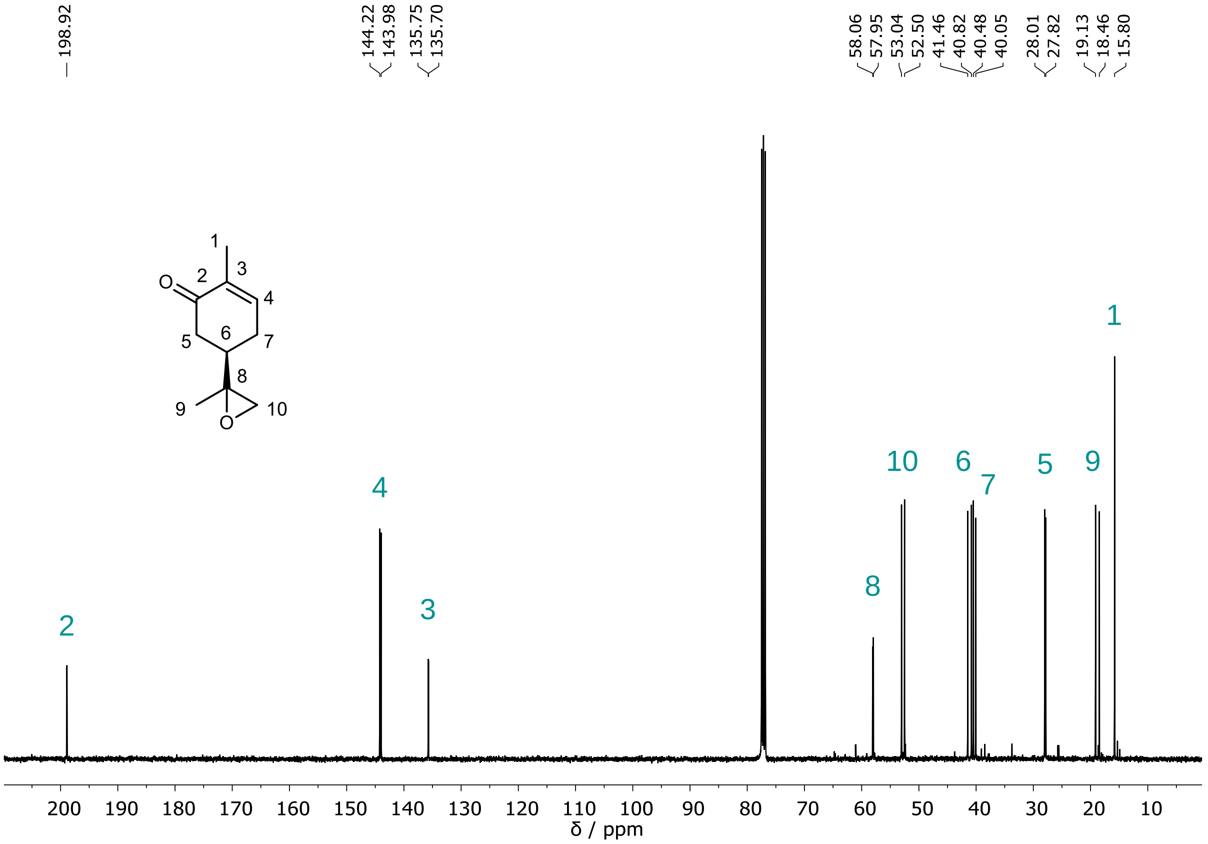

Supplementary Figure 36. **¹³C NMR spectrum of 5, measured in $CDCl_3$.**

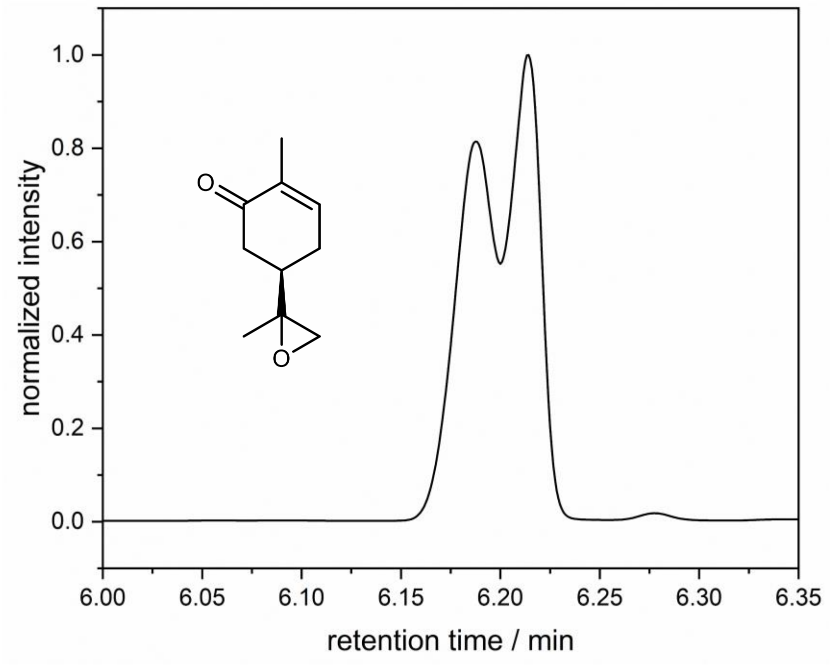

Supplementary Figure 37. Determination of diastereomeric ratio of compound 5 via GC-FID. The calculated diastereomeric ratio is 50:50.

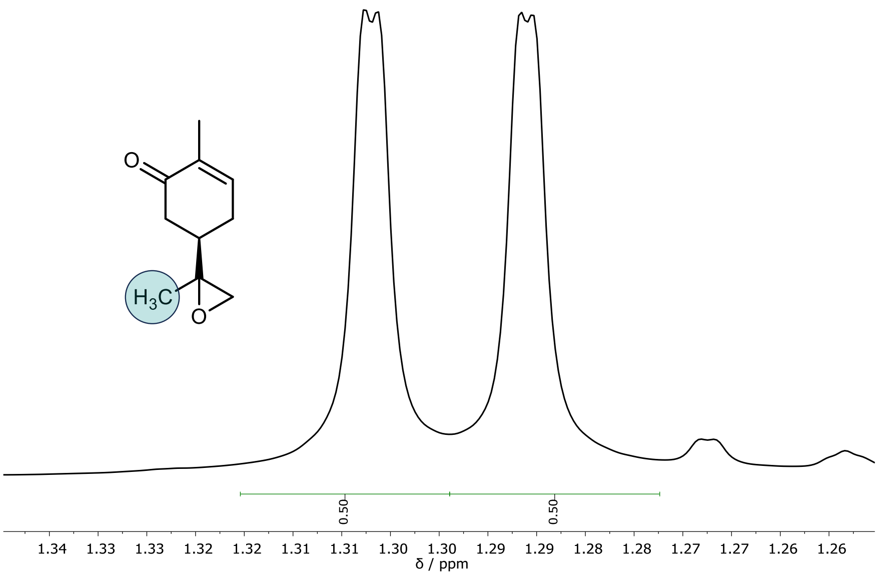

Supplementary Figure 38. Determination of diastereomeric ratio of compound 5 via NMR spectroscopy. The calculated diastereomeric ratio is 50:50.

Carvone carbonate 8

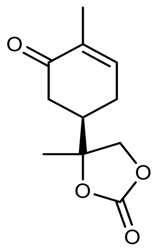

$C_{11}H_{14}O_4$
210,23 g/mol

A stainless-steel reactor with Teflon inset was charged with 3.00 g (18.0 mmol, 1.00 equiv.) carvone oxide **5**, 301 mg (1.08 mmol, 0.06 equiv.) TBACl and 30 bar CO_2 pressure. The reaction mixture was stirred and heated to 100 °C for 40 h. After completion of the reaction and cooling to room temperature, the viscous liquid was diluted with ethyl acetate (ca. 200 mL), washed with brine (3 × 50 mL) and extracted with ethyl acetate (50 mL). The combined organic phases were dried over Na_2SO_4 and the solvent was removed under reduced pressure. The residue was purified via column chromatography (cyclohexane/ethyl acetate 6:1 → 2:1), yielding 3.20 g (15.2 mmol, 85%) of the product as slightly yellow oil.

R_f (cyclohexane/ethyl acetate 1:1) = 0.30, visualized by staining with Seebach solution.

^{1}H (400 MHz, $CDCl_3$) δ / ppm = 6.84-6.67 (m, 1H, **H_4**), 4.43-4.26 (m, 1H, **H_{10}**), 4.18-4.04 (m, 1H, **H_{10}**), 2.66-2.18 (m, 5H, **H_{5-7}**), 1.89-1.72 (m, 3H, **H_1**), 1.59-1.44 (m, 3H, **H_9**).

^{13}C (100 MHz, $CDCl_3$) δ / ppm = 197.3 (**C_2**), 197.1 (**C_2**), 154.1 (**C_{11}**), 143.3 (**C_4**), 142.8 (**C_4**), 136.1 (**C_3**), 84.1 (**C_8**), 73.1 (**C_{10}**), 72.3 (**C_{10}**), 43.1 (**C_6**), 42.9 (**C_6**), 38.6 (**C_5**), 38.2 (**C_5**), 26.3 (**C_7**), 22.9 (O-C-**C**H_3), 21.7 (**C_9**), 15.7 (**C_1**).

IR (ATR platinum diamond): ṽ / cm^{-1} = 2981, 2954, 2926, 2902, 2853, 1767, 1670, 1547, 1487, 1452, 1398, 1384, 1363, 1322, 1289, 1260, 1241, 1223, 1186, 1167, 1106, 1057, 1019, 952, 906, 843, 801, 773, 721, 706, 599, 573, 557, 533, 476, 421.

ESI-MS: $[M+H]^+$ calc. 211.0965, detected 211.0960.

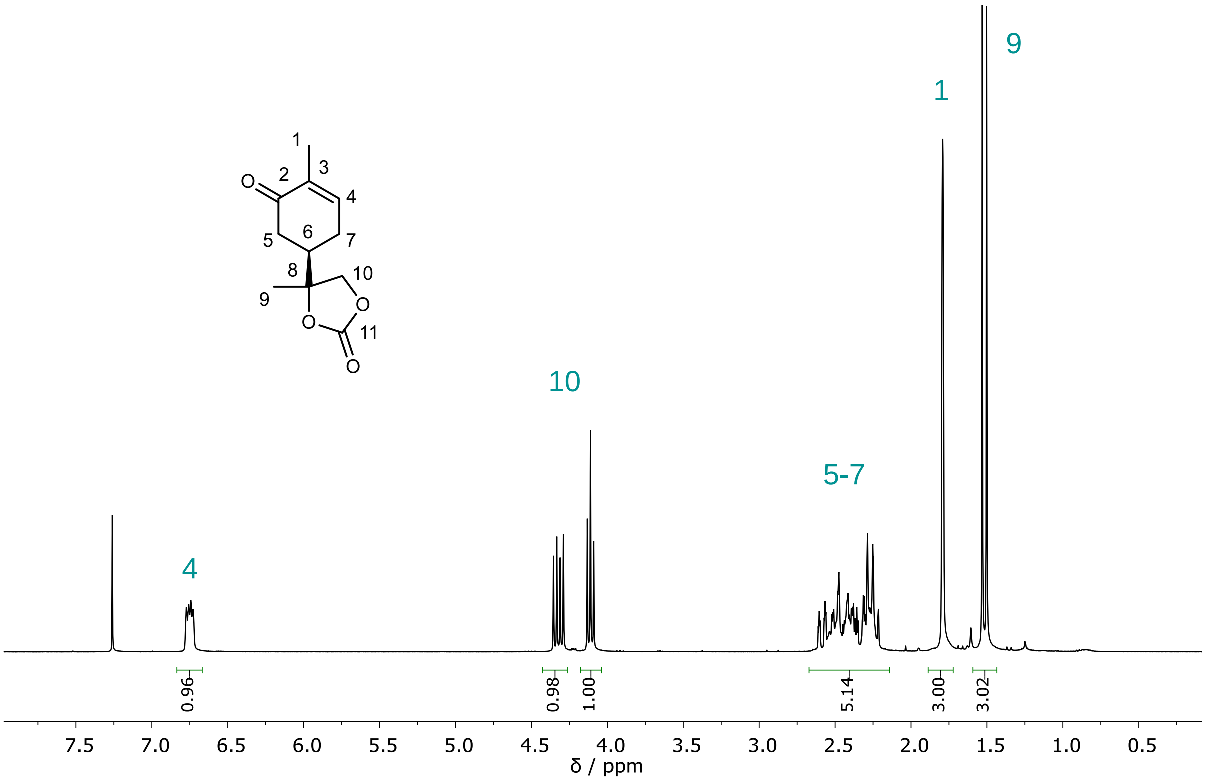

Supplementary Figure 39. ^{1}H **NMR spectrum of 8, measured in** $CDCl_3$**.**

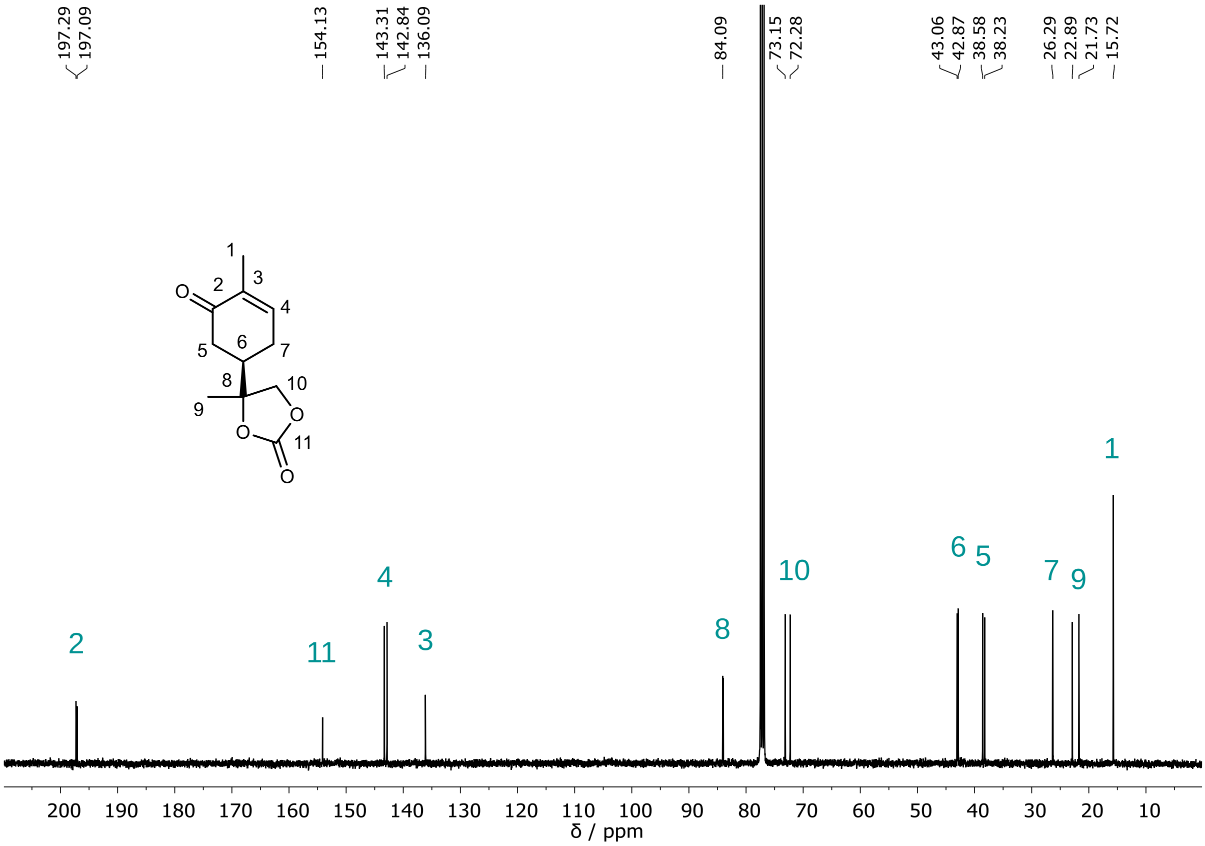

Supplementary Figure 40. ^{13}C **NMR spectrum of 8, measured in** $CDCl_3$**.**

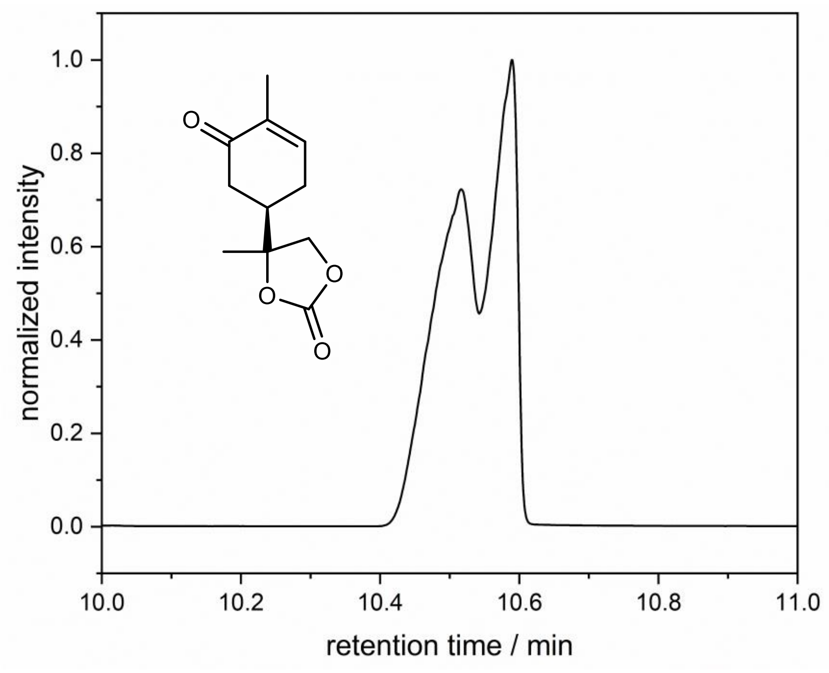

Supplementary Figure 41. Determination of diastereomeric ratio of compound 8 via GC-FID. The calculated diastereomeric ratio is 53:47.

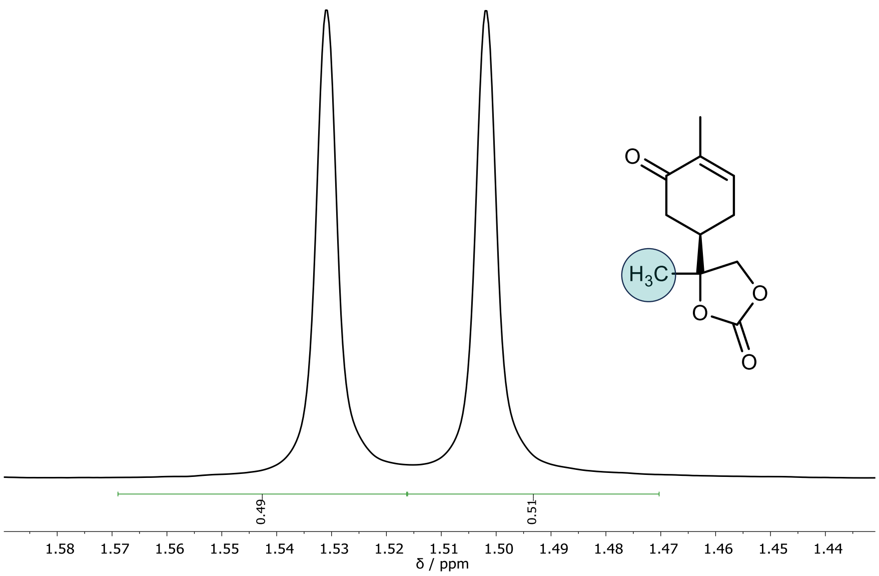

Supplementary Figure 42. Determination of diastereomeric ratio of compound 8 via NMR spectroscopy. The calculated diastereomeric ratio is 51:49.

Urethane monomer 14

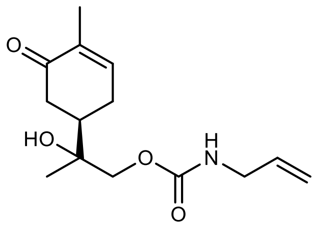

$C_{14}H_{21}NO_4$
267.32 g/mol

In a round bottom flask, 300 mg (1.43 mmol, 1.00 equiv.) carvone carbonate **8**, 214 µL (163 mg, 2.85 mmol, 2.00 equiv.) allylamine and 110 µL (110 mg, 0.71 mmol, 0.50 equiv.) DBU were dissolved in 2.0 mL DMSO (20 equiv.). The mixture was heated to 40 °C and stirred for 6 h. The progress of the reaction was controlled via TLC. The reaction mixture was dissolved in ethyl acetate (~50 mL), washed with brine (3 ×) and extracted with ethyl acetate. After removal of the solvent, the mixture was purified via column chromatography (cyclohexane/ ethyl acetate 3:1 → 1:2), yielding 233 mg (0.87 mmol, 61%) of the slightly product as a slightly yellow oil.

R_f (cyclohexane/ethyl acetate 1:1) = 0.17, visualized by staining with Seebach solution.

^{1}H (400 MHz, $CDCl_3$) δ / ppm = 6.83-6.67 (m, 1H, **H_4**), 5.91-5.74 (m, 1H, **H_{15}**), 5.25-5.09 (m, 2H, **H_{16}**), 5.09-4.80 (m, 1H, **H_{13}**), 4.21-3.94 (m, 2H, **H_{11}**), 3.86-3.70 (m, 2H, **H_{14}**), 2.83-2.17 (m, 6H, **H_5**, **H_6**, **H_7**, **H_{10}**), 1.82-1.71 (m, 3H, **H_1**), 1.21-1.11 (m, 3H, **H_9**).

^{13}C (100 MHz, $CDCl_3$) δ / ppm = 200.0 (**C_2**), 199.7 (**C_2**), 156.8 (**C_{12}**), 156.6 (**C_{12}**), 145.3 (**C_4**), 144.5 (**C_4**), 135.5 (**C_3**), 135.3 (**C_3**), 134.2 (**C_{15}**), 134.1 (**C_{15}**), 116.5 (**C_{16}**), 72.8 (**C_8**), 72.7 (**C_8**), 70.3 (**C_{11}**), 70.2 (**C_{11}**), 43.6 (**C_{14}**), 42.4 (**C_6**), 42.1 (**C_6**), 39.5 (**C_5**), 38.8 (**C_5**), 27.3 (**C_7**), 26.5 (**C_7**), 21.3 (**C_9**), 21.2 (**C_9**), 15.7 (**C_1**).

IR (ATR platinum diamond): ṽ / cm^{-1} = 3342, 3082, 2979, 2952, 2924, 2905, 1699, 1659, 1527, 1452, 1433, 1370, 1242, 1109, 1058, 1017, 994, 922, 832, 801, 776, 711, 634, 556, 468.

ESI-MS: $[M+H]^+$ calc. 268.1543, detected 268.1541.

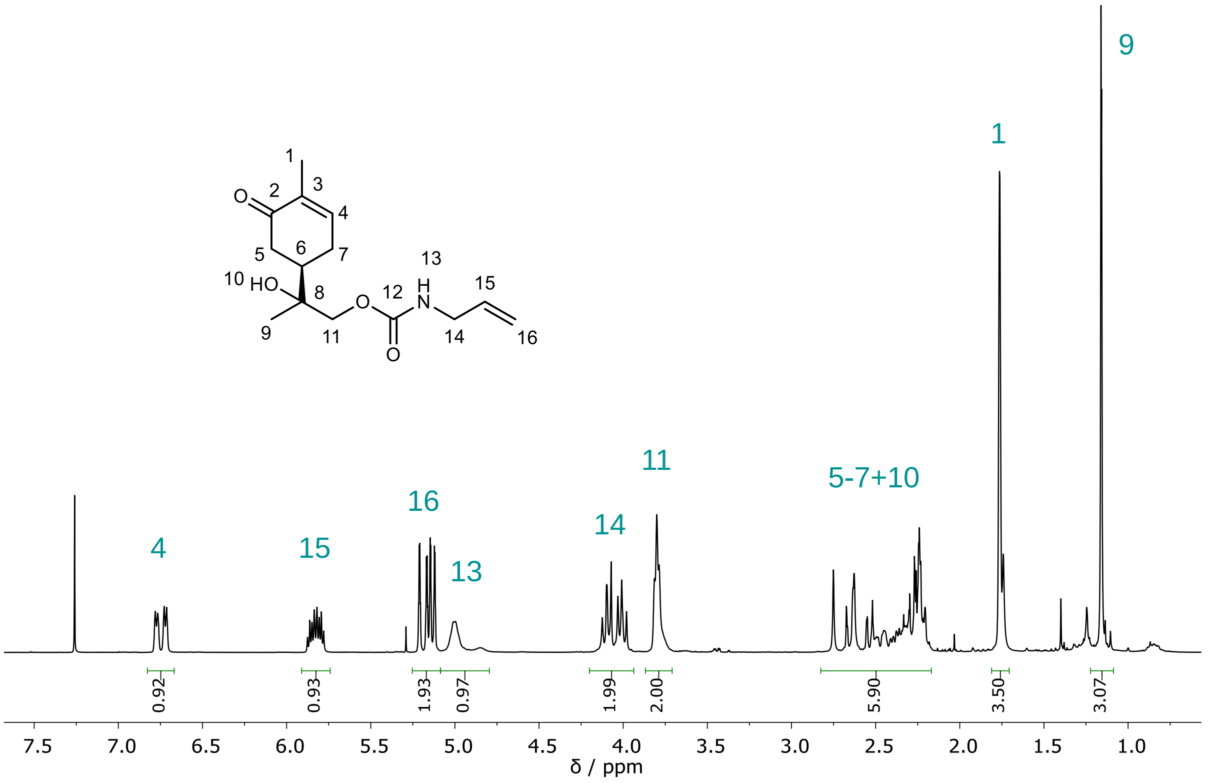

Supplementary Figure 43. ^{1}**H NMR spectrum of 14, measured in CDCl**$_3$**.**

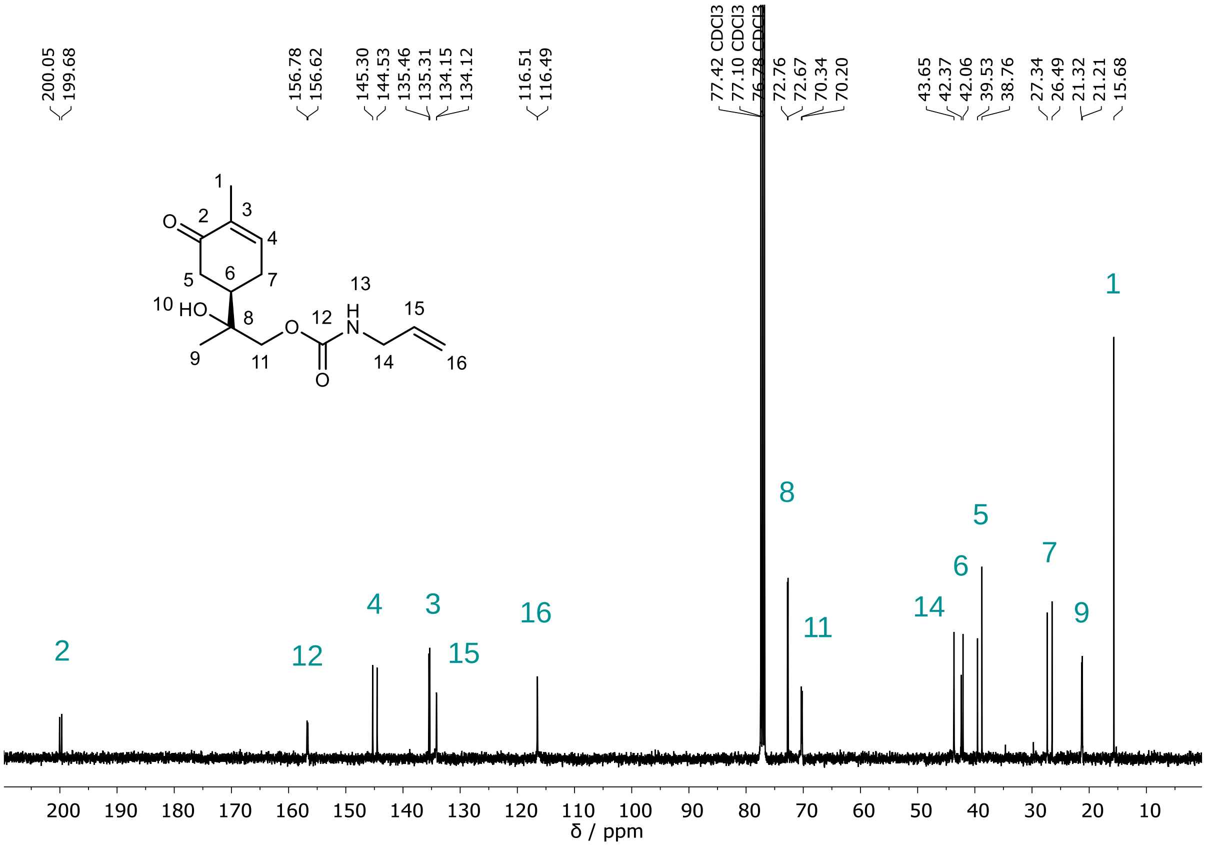

Supplementary Figure 44. ^{13}**C NMR spectrum of 14, measured in CDCl**$_3$**.**

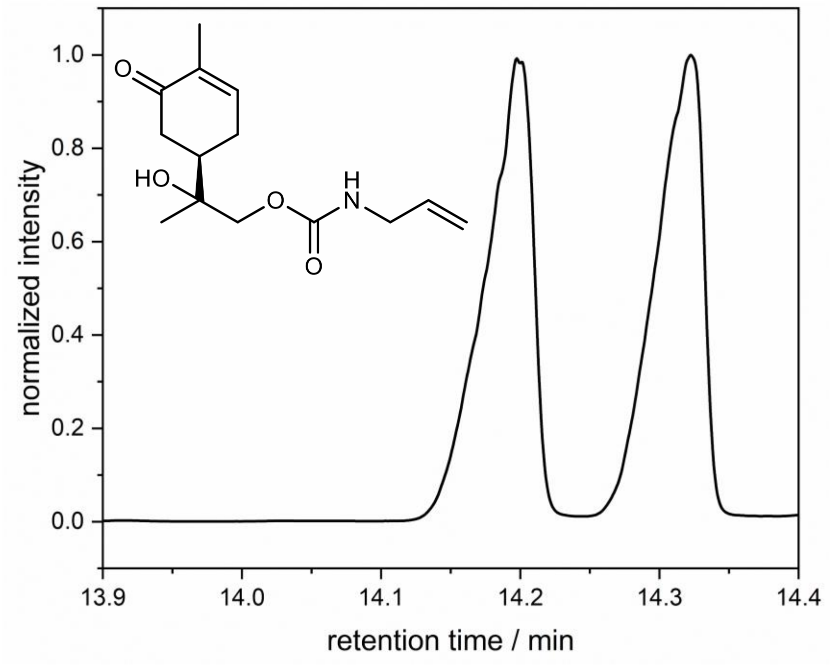

Supplementary Figure 45. Determination of diastereomeric ratio of compound 14 via GC-FID. The calculated diastereomeric ratio is 50:50.

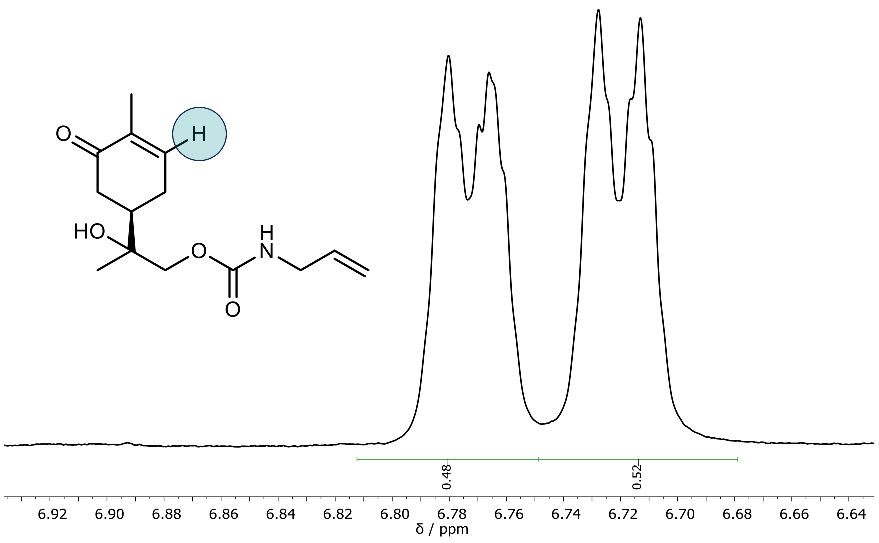

Supplementary Figure 46. Determination of diastereomeric ratio of compound 14 via NMR spectroscopy. The calculated diastereomeric ratio is 52:48.

4.4 Synthesis of monomers 18 and 19

Methyl undec-10-enoate 23

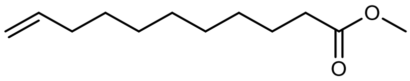

$C_{12}H_{22}O_2$
198.31 g/mol

In a round bottom flask, 82.0 g (445 mmol, 1.00 equiv.) undec-10-enoic acid were dissolved in in 200 mL methanol. 5.93 mL, (10.9 g, 111 mmol, 0.25 equiv.) H_2SO_4 were added and the reaction mixture was stirred under reflux conditions for 25 h. After cooling to room temperature, the solution was neutralized with sodium bicarbonate (21 g). The solution was filtered and the solvent was removed under reduced pressure. The residue was diluted in ethyl acetate (200 mL) and washed with saturated $NaHCO_3$ solution (3 × 50 mL), water (50 mL) and brine (50 mL). The organic phase was dried over Na_2SO_4 and the solvent was removed under reduced pressure. The crude mixture was purified via vacuum distillation (p = 30 mbar, T = 165 °C), yielding 71.7 g (362 mmol, 81 %) of the product as a colorless liquid.

R_f (*n*-hexane/diethyl ether 7:3) = 0.67, visualized by staining with Seebach solution.

^{1}H (400 MHz, $CDCl_3$) δ / ppm = 5.80 (ddt, *J* = 17.0, 10.2, 6.7 Hz, 1H, **H_2**), 5.07-4.87 (m, 2H, **H_1**), 3.66 (s, 3H, **H_{12}**), 2.30 (t, *J* = 7.6 Hz, 2H, **H_{10}**), 2.03 (tdd, *J* = 6.5, 5.3, 1.5 Hz, 2H, **H_3**), 1.71-1.54 (m, 2H, **H_9**), 1.47-1.19 (m, 10H, **$H_{4\text{-}8}$**).

^{13}C (100 MHz, $CDCl_3$) δ / ppm = 174.5 (**C_{11}**), 139.3 (**C_2**), 114.3 (**C_1**), 51.6 (**C_{12}**), 34.2 (**C_{10}**), 33.9 (**C_3**), 29.4 (**$C_{4\text{-}8}$**), 29.3 (**$C_{4\text{-}8}$**), 29.2 (**$C_{4\text{-}8}$**), 29.0 (**$C_{4\text{-}8}$**), 25.1 (**C_9**).

IR (ATR platinum diamond): ṽ / cm^{-1} = 3077, 2976, 2926, 2855, 1740, 1641, 1436, 1361, 1318, 1239, 1196, 1169, 1116, 1047, 994, 909, 882, 858, 772, 724, 635, 589, 554, 434.

ESI-MS: $[M+H]^+$ calc. 199.1693, detected 199.1691.

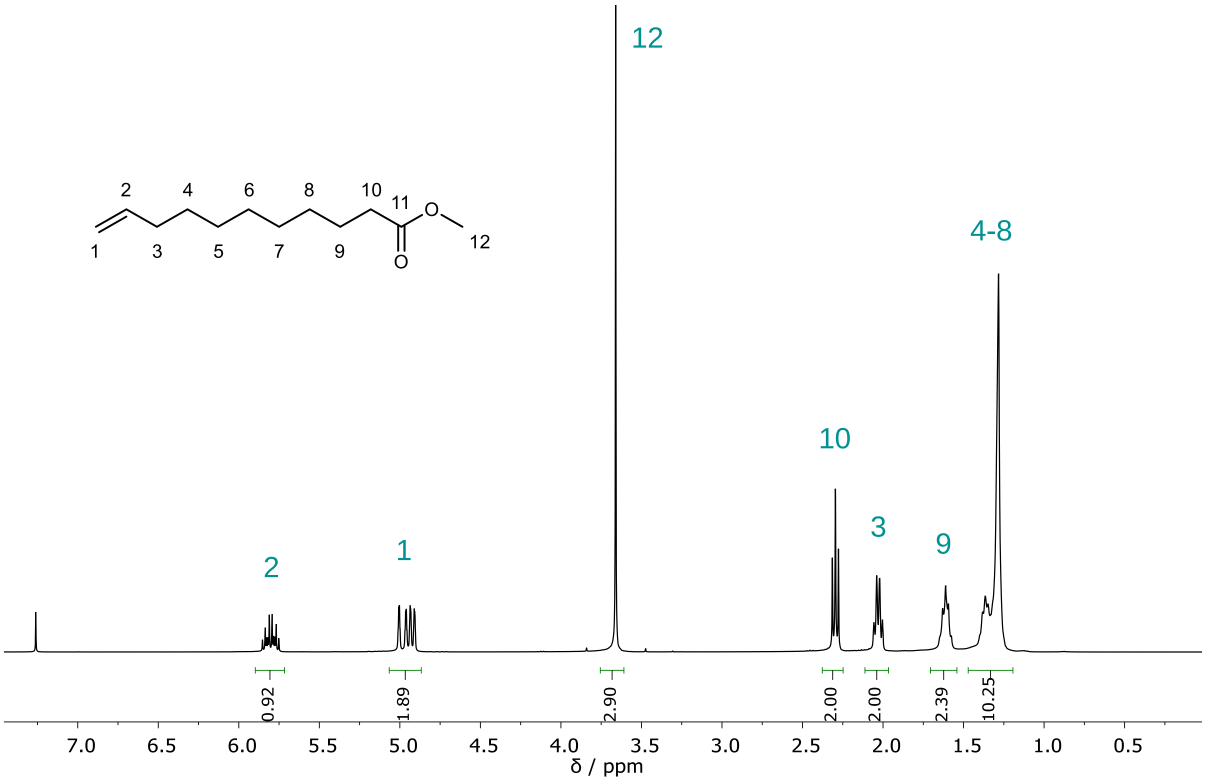

Supplementary Figure 47. ^{1}H NMR spectrum of 23, measured in $CDCl_3$.

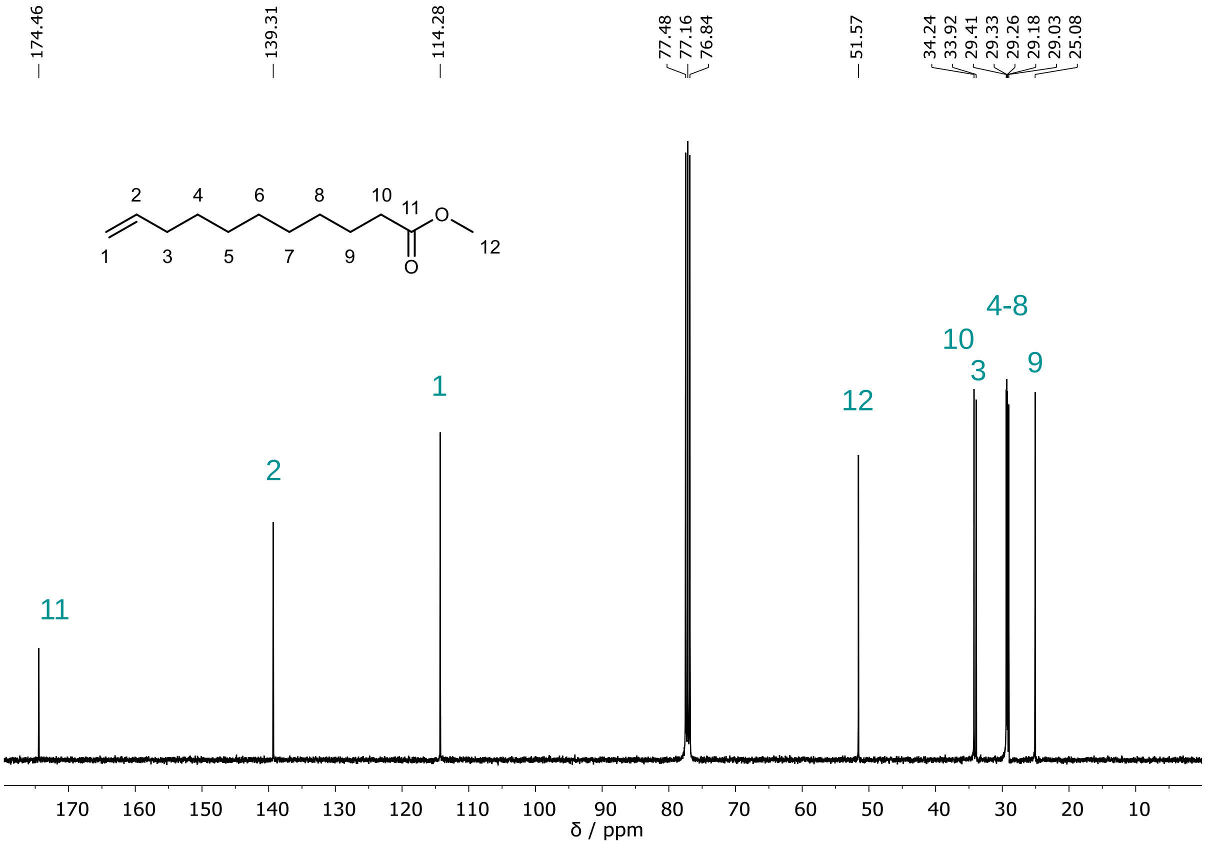

Supplementary Figure 48. ^{13}C NMR spectrum of 23, measured in $CDCl_3$.

***N*-Hydroxyundec-10-enamide 24**

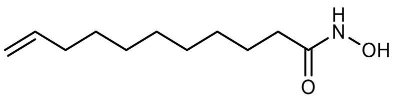

$C_{11}H_{21}NO_2$
199.29 g/mol

20.3 g (110 mmol, 1.00 equiv.) methyl 10-undecenoate **23** and 10.7 g (154 mmol, 1.50 equiv.) hydroxylamine hydrochloride were suspended in 460 mL methanol. After adding 17.1 g (305 mmol, 3.00 equiv.) KOH, the mixture was stirred strongly under reflux conditions over night. After completion of the reaction (control via TLC), the mixture was brought to a pH of 5 using 25% acetic acid. Part of the reaction mixture was removed under reduced pressure. The residue was diluted with water (100 mL) and ethyl acetate (300 mL). The organic phase was separated, dried over Na_2SO_4 and the solvent was removed under reduced pressure. Recrystallization (*n*-hexane/ethyl acetate 5:1) yielded 7.46 g (39.9 mmol, 36%) of the product as colorless crystals.

R$_f$ (ethyl acetate) = 0.59, visualized by staining with Seebach solution.

^{1}H (400 MHz, $CDCl_3$) δ / ppm = 8.31 (bs, 2H, **H$_{12}$**, **H$_{13}$**), 5.91-5.71 (m, 1H, **H$_2$**), 5.07-4.86 (m, 2H, **H$_1$**), 2.14 (t, *J* = 7.5 Hz, 1H, **H$_{10}$**), 2.03 (q, *J* = 6.9 Hz, 1H, **H$_3$**), 1.72-1.56 (m, 2H, **H$_9$**), 1.42-1.20 (m, 10H, **H$_{4-8}$**).

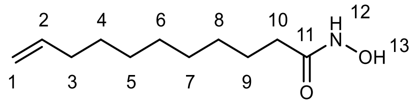

^{13}C (100 MHz, $CDCl_3$) δ / ppm = 171.7 (**C$_{11}$**), 139.3 (**C$_2$**), 114.3 (**C$_1$**), 33.9 (**C$_3$**), 33.1 (**C$_{10}$**), 29.4 (**C$_{4-8}$**), 29.3 (**C$_{4-8}$**), 29.2 (**C$_{4-8}$**), 29.0 (**C$_{4-8}$**), 25.4 (**C$_9$**).

IR (ATR platinum diamond): ṽ / cm^{-1} = 3259, 3081, 3000, 2980, 2945, 2914, 2847, 2701, 1834, 1659, 1642, 1606, 1570, 1468, 1428, 1383, 1327, 1290, 1273, 1234, 1121, 1070, 1043, 1022, 993, 967, 913, 850, 759, 745, 720, 656, 548, 477, 449, 425.

ESI-MS: $[M+H]^+$ calc. 200.1645, detected 200.1644.

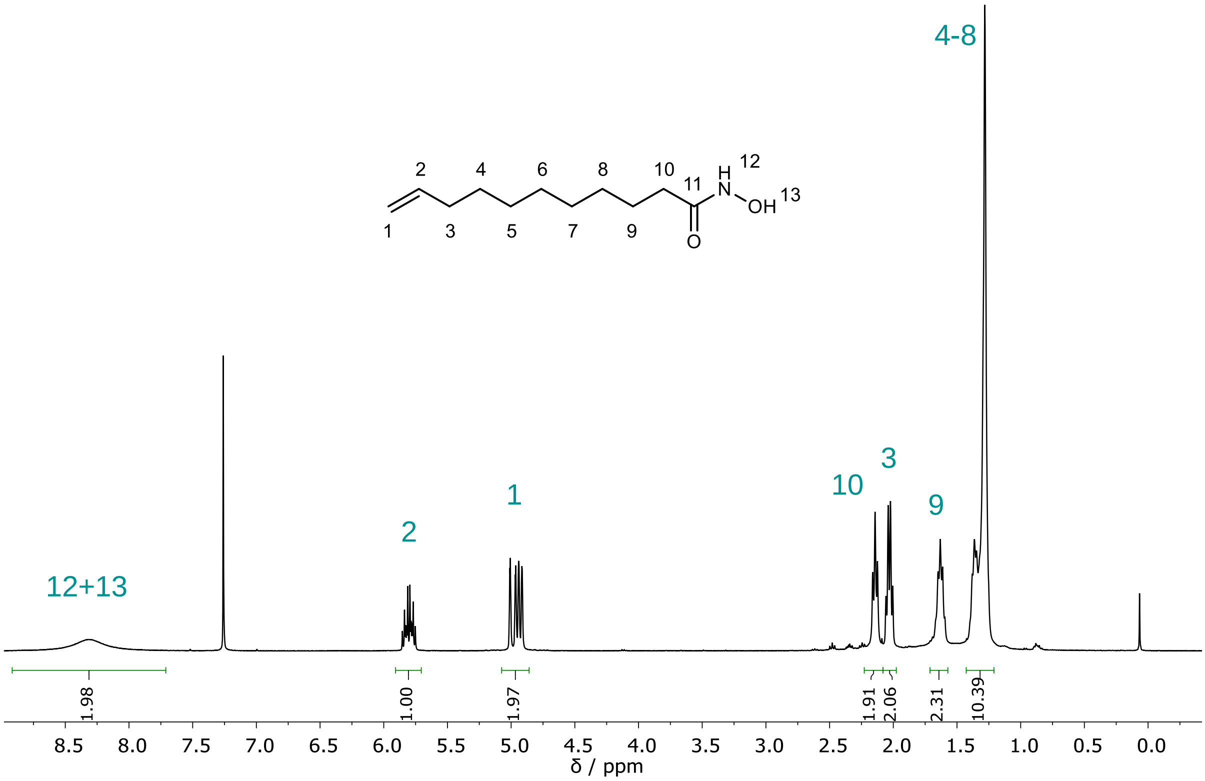

Supplementary Figure 49. ^{1}H **NMR spectrum of 24, measured in** $CDCl_3$**.**

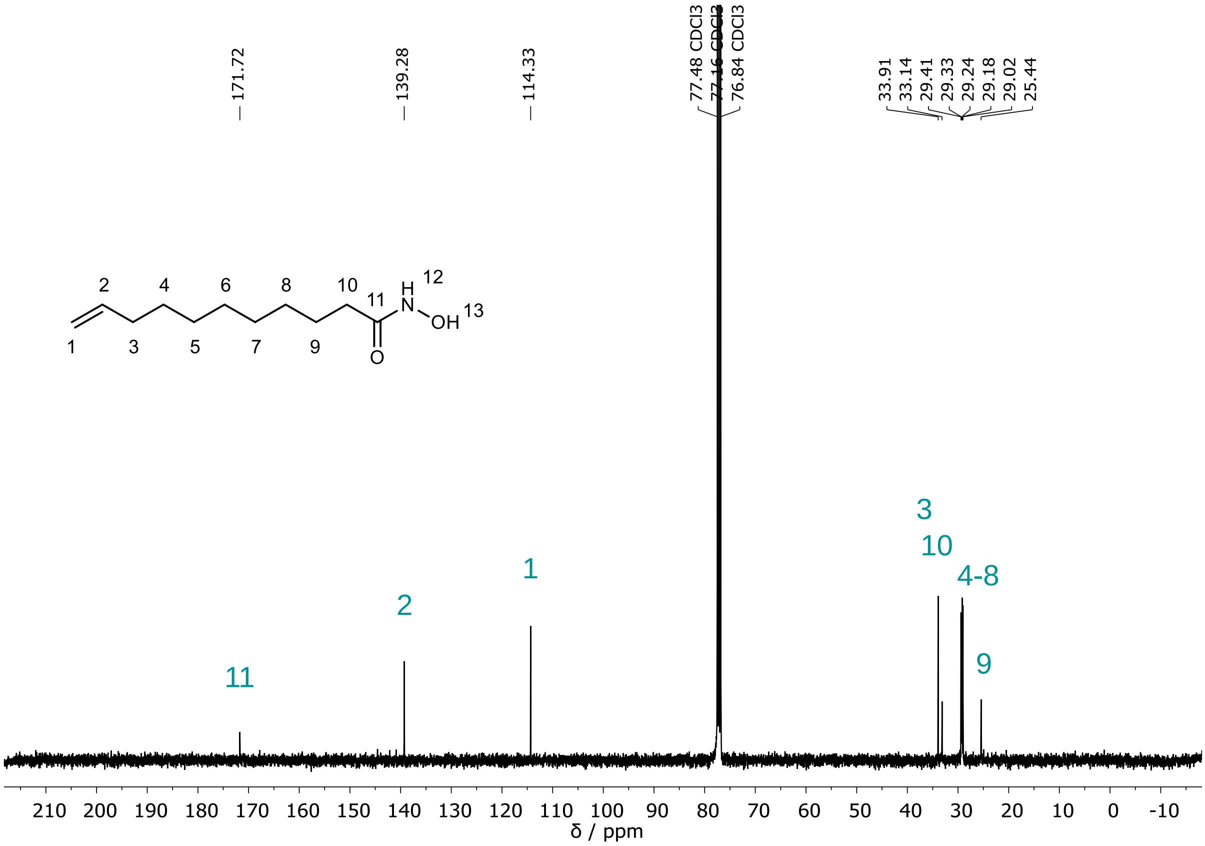

Supplementary Figure 50. ^{13}C **NMR spectrum of 24, measured in** $CDCl_3$**.**

Methyl dec-9-en-1-ylcarbamate 25

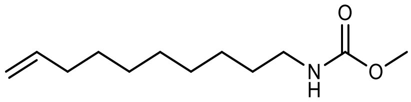

$C_{12}H_{23}NO_2$
213.32 g/mol

7.11 g (35.7 mmol, 1,00 equiv.) *N*-hydroxyundec-10-enamide **24** was dissolved in 59 mL (64 g, 700 mmol, 20 equiv.) dimethyl carbonate and 3.0 mL (2.3 g, 70 mmol, 2.0 equiv.) methanol. The mixture was heated under reflux conditions and 980 mg (7.0 mmol, 0.20 equiv.) TBD were added. After completion of the reaction (control via TLC), the mixture was evaporated to dryness. Purification via column chromatography (*n*-hexane/ethyl acetate 9:1 → 7:3) yielded 4.61 g (21.6 mmol, 61%) of the product as a colorless oil.

R_f (cyclohexane/ethyl acetate 5:1) = 0.44, visualized by staining with Seebach solution.

^{1}H (400 MHz, $CDCl_3$) δ / ppm = 5.89-5.70 (m, 1H, **H_2**), 5.05-4.86 (m, 2H, **H_1**), 4.78-4.38 (m, 1H, **H_{11}**), 3.65 (s, 3H, **H_{13}**), 3.25-3.02 (m, 2H, **H_{10}**), 2.11-1.97 (m, 2H, **H_3**), 1.56-1.42 (m, 2H, **H_9**), 1.42-1.16 (m, 10H, **H_{4-8}**).

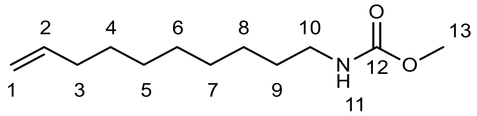

^{13}C (100 MHz, $CDCl_3$) δ / ppm = 157.2 (**C_{12}**), 139.3 (**C_2**), 114.3 (**C_1**), 52.1 (**C_{13}**), 41.2 (**C_{10}**), 33.9 (**C_3**), 30.1 (**C_9**), 29.5 (**C_{4-7}**), 29.3 (**C_{4-7}**), 29.2 (**C_{4-7}**), 29.0 (**C_{4-7}**), 26.8 (**C_8**).

IR (ATR platinum diamond): ṽ / cm^{-1} = 3334, 3077, 2925, 2854, 1699, 1640, 1532, 1463, 1443, 1367, 1346, 1250, 1193, 1143, 1112, 1037, 994, 908, 851, 779, 723, 687, 634, 555.

ESI-MS: $[M+H]^+$ calc. 214.1802, detected 214.1800.

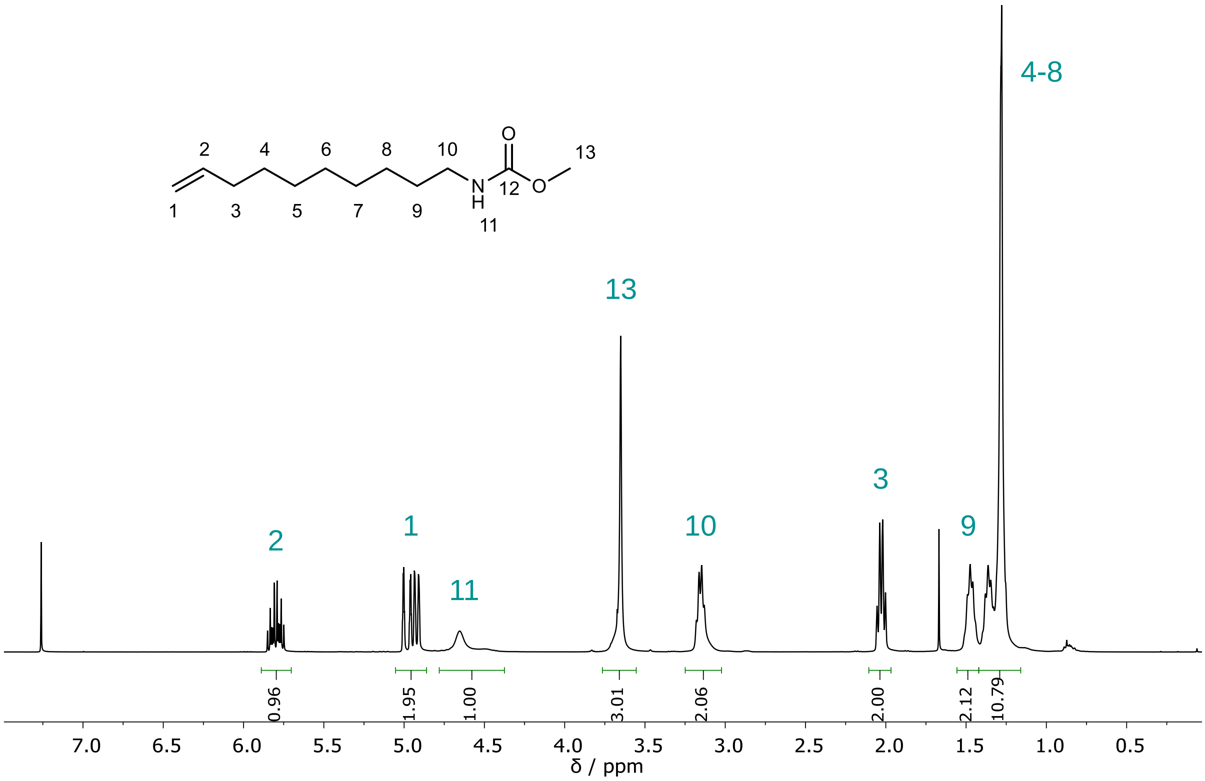

Supplementary Figure 51. ^{1}H NMR spectrum of 25, measured in $CDCl_3$.

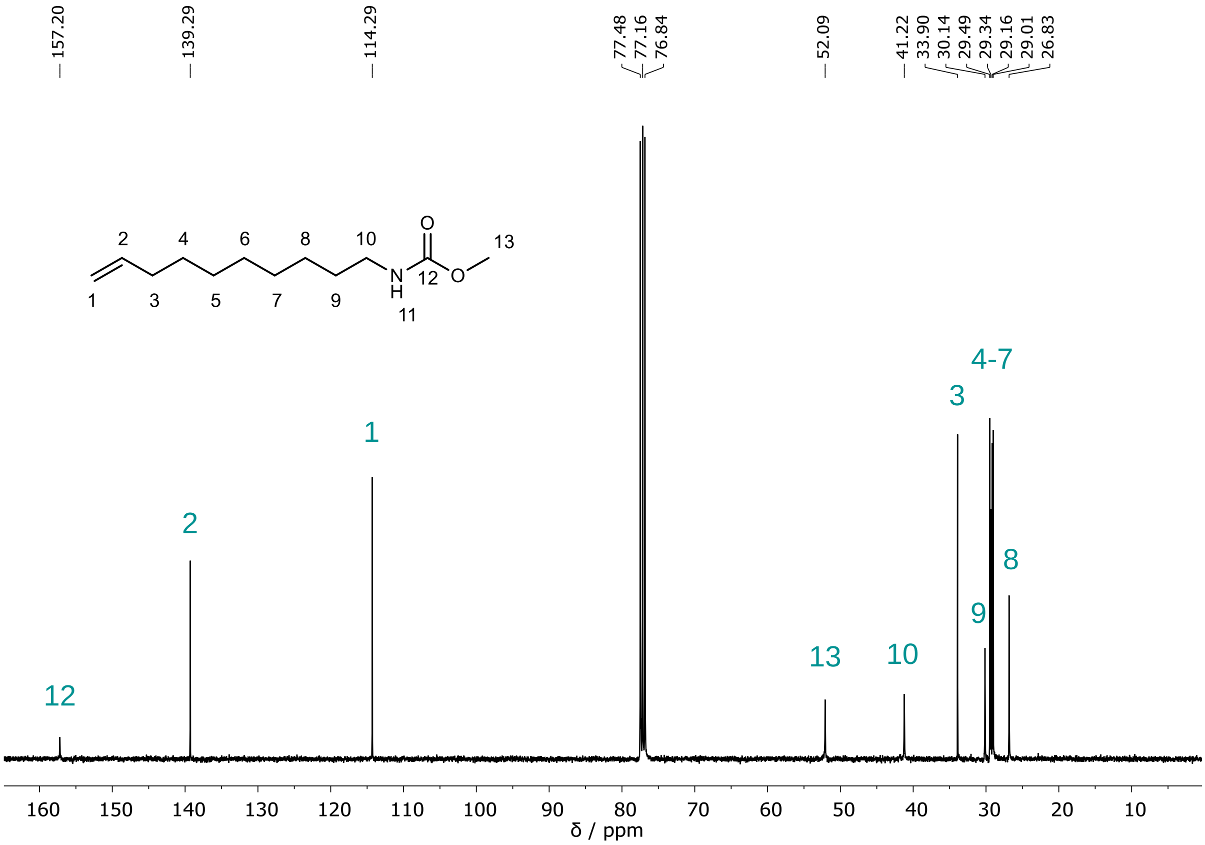

Supplementary Figure 52. ^{13}C NMR spectrum of 25, measured in $CDCl_3$.

Dec-9-en-1-amine 17

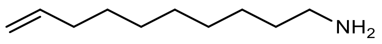

$C_{10}H_{21}N$
155.28 g/mol

5.00 g (23.4 mmol, 1.00 equiv.) methyl dec-9-enylcarbamate **25** were dissolved in isopropanol (65 mL) and 16 M potassium hydroxide solution (45 mL) was added. With strong stirring, the mixture was heated to reflux for 16 hours until TLC showed full conversion. After cooling to room temperature, diethyl ether (100 mL) and water (50 mL) were added. After separation of the water layer, the organic layer was washed with brine (80 mL). Subsequently, the organic solution was dried over sodium sulfate and then evaporated to dryness to obtain 3.74 g of dec-9-en-1-amine as colorless oil in a purity of 82% according to measurements with remaining impurity of unreacted **25**, corresponding to a yield of 2.87 g (18.5 mmol, 79%). The mixture was used directly for the synthesis of monomers **18** and **19** without further purification.

^{1}H NMR (400 MHz, $CDCl_3$) δ / ppm = 5.80 (ddt, *J* = 16.9, 10.2, 6.7 Hz, 1H, **H_2**), 5.05-4.84 (m, 2H, **H_1**), 2.66 (t, *J* = 7.0 Hz, 2H, **H_{10}**), 2.02 (q, *J* = 6.9 Hz, 2H, **H_3**), 1.70-1.53 (m, 2H, **H_{11}**) 1.52-1.39 (m, 2H, **H_9**), 1.39-1.32 (m, 2H, **H_{4-7}**), 1.32-1.23 (m, 8H, **H_{4-8}**).

^{13}C (100 MHz, $CDCl_3$) δ / ppm = 139.3 (**C_2**), 114.2 (**C_1**), 42.3 (**C_{10}**), 33.9 (**C_3**, **C_9**), 29.6 (**C_{4-7}**), 29.2 (**C_{4-7}**), 29.0 (**C_{4-7}**), 27.0 (**C_{4-7}**), 26.9 (**C_{4-7}**), 22.3 (**C_8**).

IR (ATR platinum diamond): ṽ / cm^{-1} = 3374, 3240, 3076, 2976, 2923, 2853, 1822, 1715, 1640, 1557, 1464, 1384, 1371, 1305, 1258, 1178, 1142, 1114, 993, 908, 819, 723, 634, 554, 442, 418.

ESI-MS: $[M+H]^+$ calc. 156.1747, detected 156.1746.

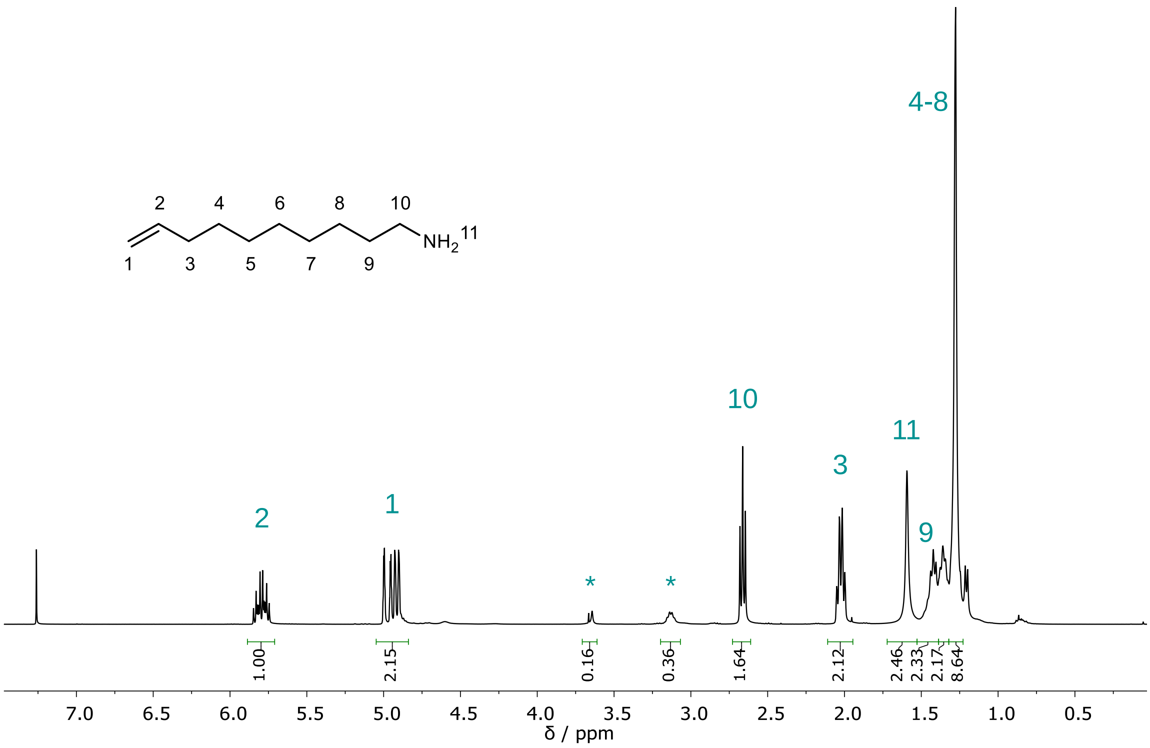

Supplementary Figure 53. ¹H NMR spectrum of 17, measured in $CDCl_3$. * marks impurities of unreacted **25**.

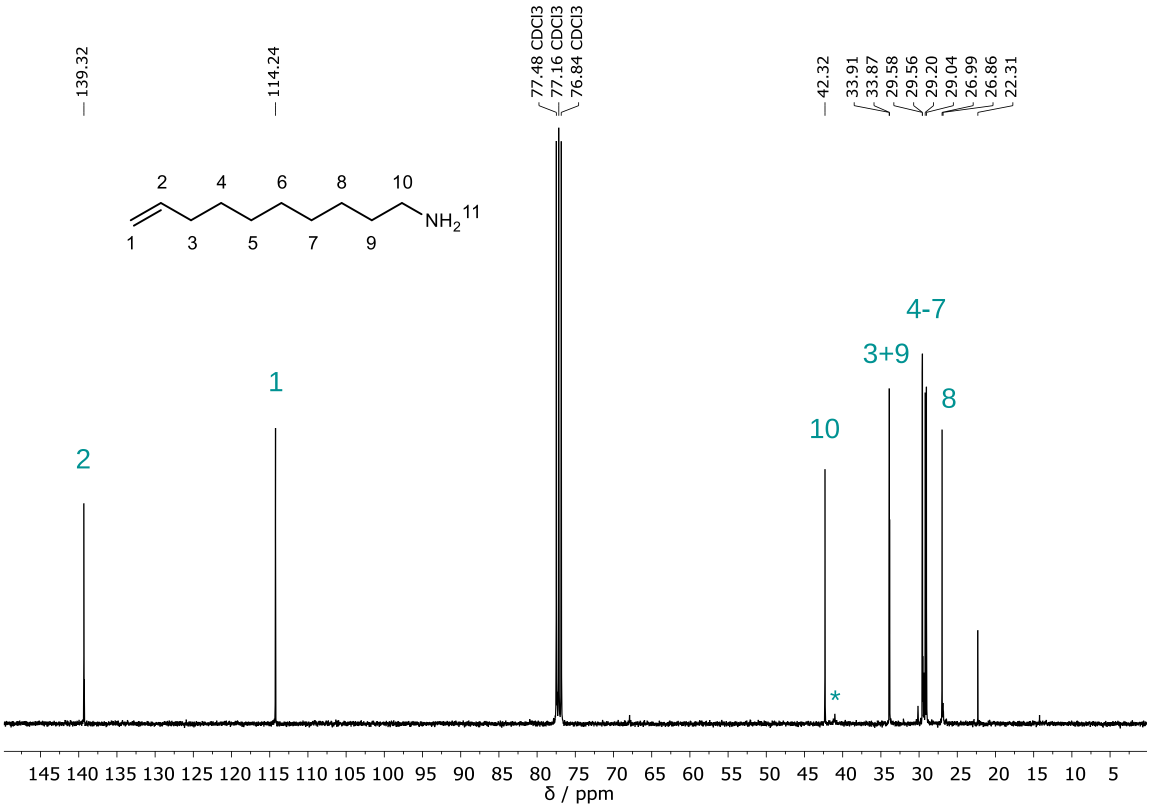

Supplementary Figure 54. ¹³C NMR spectrum of 17, measured in $CDCl_3$. * marks impurities of unreacted **25**.

Urethane monomer 18

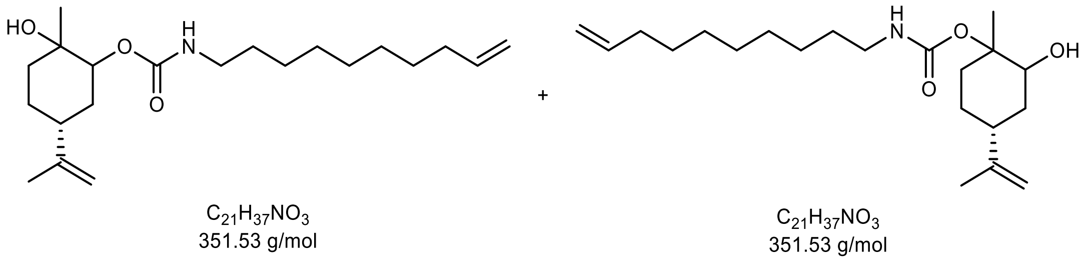

In a 5 mL pressure vial, 89 mg (0.241 mmol, 5.0 mol%) *N*-(3,5-bis(trifluormethyl)phenyl)-*N'*-cyclohexyl thiourea **9** were added to 947 mg (4.83 mmol, 1.00 equiv.) limonene dicarbonate **7** and 1.50 g (9.66 mmol, 2.00 equiv.) dec-9-en-1-amine **17**. The vial was sealed and the mixture was stirred at 70 °C. After completion of the reaction (reaction control via TLC), the mixture was purified via column chromatography (cyclohexane/ethyl acetate 20:1 → 1:1) and 1.17 g (3.34 mol, 69%) of the product were obtained as slightly yellow oil.

$\mathbf{R_f}$ (cyclohexane/ethyl acetate 3:1) = 0.25, visualized by staining with Seebach solution.

^{1}H (400 MHz, CDCl$_3$) δ / ppm = 5.85-5.65 (m, **H_2**), 5.01-4.81 (m, **H_1**) 4.81-4.68 (m, **H_{11}**), 4.68-4.61 (s, **H_{23}**), 4.61-4.49 (m, **H_{15a}**), 3.46-3.25 (m, **H_{15b}**), 3.18-2.99 (m, **H_{10}**), 2.24-2.08 (m, **H_{17}**), 2.06-1.89 (m, **H_3**, **H_{20}**), 1.89-1.70 (m, **H_{17}**, **H_{19a}**, **H_{19b}**), 1.70-1.63 (m, **H_{22}**), 1.62-1.48 (m, **H_{13b}**, **H_{19a}**), 1.48-1.37 (m, **H_{4-9}**, **H_{18}**, **H_{19b}**), 1.37-1.27 (m, **H_{4-9}**, **H_{17}**, **H_{18}**), 1.27-1.17 (m, **H_{4-9}**), 1.17-0.97 (m, **H_{13a}**).

Due to the occurrence of isomers, no integral values are given for the respective NMR signals. The sum of integrals matches the expected number of protons. The purity of the product was confirmed via SEC measurements. The signal corresponding to the OH-proton 14 could not be assigned unambiguously due to overlapping signals.

^{13}C (100 MHz, CDCl$_3$) δ / ppm = 156.7 (**C_{12}**), 156.0 (**C_{12}**), 149.0 (**C_{21}**), 148.7 (**C_{21}**), 139.2 (**C_2**), 114.2 (**C_1**), 109.0 (**C_{23}**), 108.7 (**C_{23}**), 83.7 (**C_{16b}**), 77.8 (**C_{15a}**), 76.0 (**C_{15b}**), 70.6 (**C_{16a}**), 44.0 (**C_{20}**), 43.5 (**C_{20}**), 41.1 (**C_{10}**), 37.4 (**C_{17}**), 37.2 (**C_{17}**), 36.2 (**C_{19b}**), 33.8 (**C_3**), 32.3 (**C_{19a}**), 29.9 (**C_{4-9}**), 29.4 (**C_{4-9}**), 29.2 (**C_{4-9}**), 29.0 (**C_{4-9}**), 28.9 (**C_{4-9}**), 27.2 (**C_{13a}**), 26.7 (**C_{4-9}**), 26.1 (**C_{18}**), 26.0 (**C_{18}**), 22.3 (**C_{13b}**), 21.0 (**C_{22}**), 20.8 (**C_{22}**).

IR (ATR platinum diamond): ṽ / cm^{-1} = 3328, 3077, 2926, 2855, 1683, 1643, 1531, 1454, 1440, 1403, 1374, 1248, 1187, 1142, 1076, 1013, 994, 943, 908, 887, 851, 775, 723, 632, 545, 450, 606, 582, 514, 490, 422.

ESI-MS: $[M+H]^+$ calc. 352.2846, detected 352.2842.

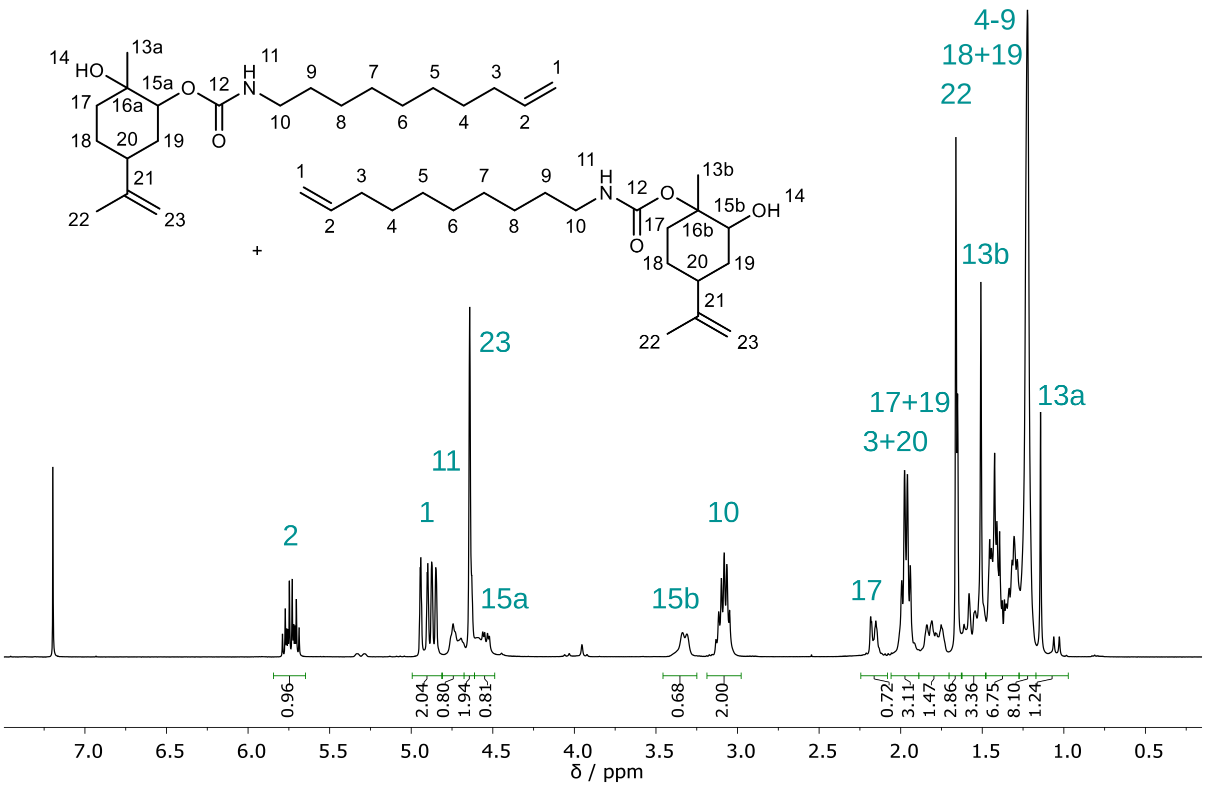

Supplementary Figure 55. ^{1}H NMR spectrum of 18, measured in $CDCl_3$.

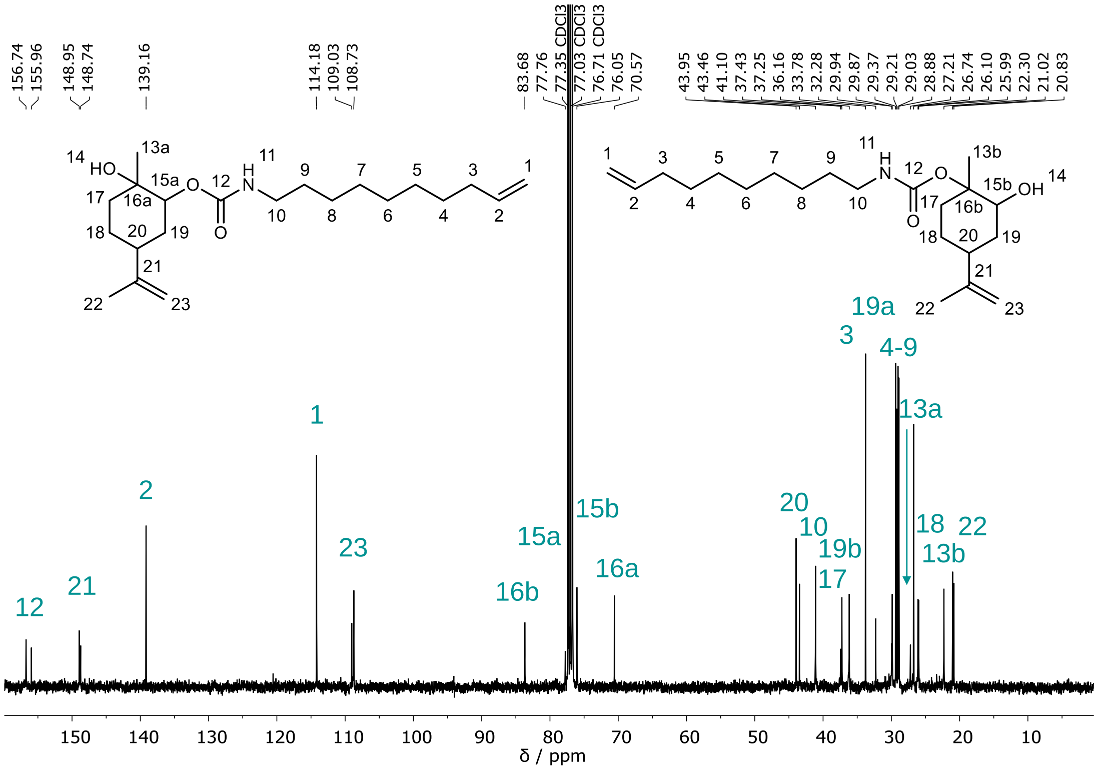

Supplementary Figure 56. ^{13}C NMR spectrum of 18, measured in $CDCl_3$.

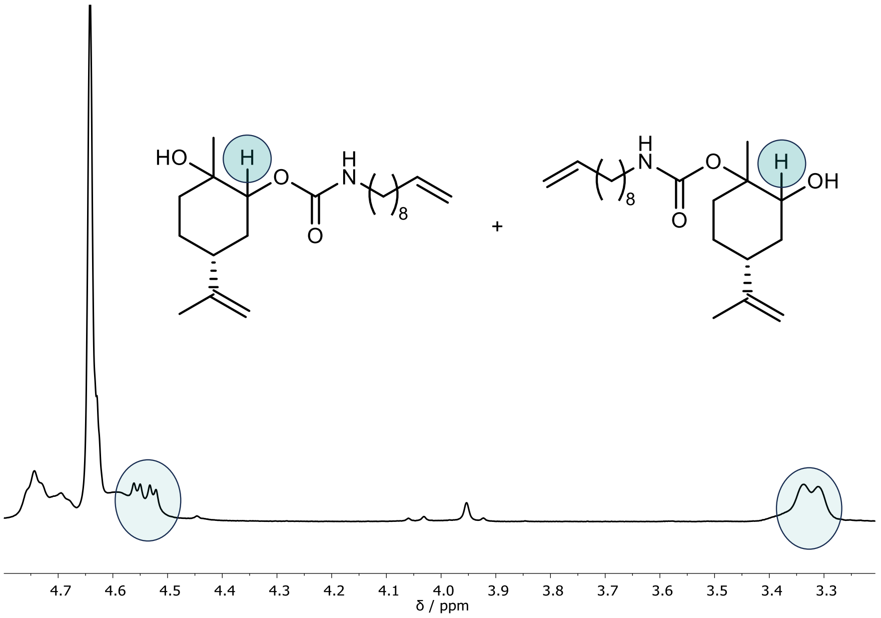

Supplementary Figure 57. Observation of different regioisomers of compound 18 via NMR spectroscopy.

As compound **18** was not sufficiently volatile to analyze via GC-FID, the occurring of regioisomers could only be observed via NMR measurements. Due to overlapping signals, no determination of a regioisomeric ratio was possible.

Urethane monomer 19

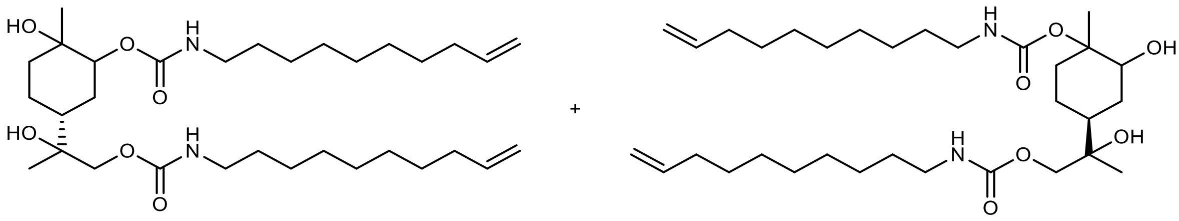

$C_{32}H_{58}N_2O_6$
566.82 g/mol

$C_{32}H_{58}N_2O_6$
566.82 g/mol

In a 5 mL pressure vial, 160 mg (0.43 mmol, 10 mol%) *N*-(3,5-bis(trifluormethyl)phenyl)-*N*'-cyclohexyl thiourea **9** were added to 1.10 g (4.29 mmol, 1.00 equiv.) limonene dicarbonate **9** and 2.00 g (12.9 mmol, 3.00 equiv.) dec-9-en-1-amine **17**. The vial was sealed and the mixture was stirred at 70 °C. After stirring for 6 days and with 334 mg (2.15 mmol, 0.500 equiv.) added after 4 days, the mixture was purified via column chromatography (dichloromethane/acetone 20:1 → 4:1) and 361 mg (0.637 mol, 15%) of the product were obtained as colorless, viscous liquid.

$\mathbf{R_f}$ (dichloromethane/acetone 4:1) = 0.38, visualized by staining with vanillin solution.

^{1}H (400 MHz, CDCl$_3$) δ / ppm = 5.89-5.71 (m, $\mathbf{H_{2}}$, $\mathbf{H_{35}}$), 5.04-4.89 (m, $\mathbf{H_{1}}$, $\mathbf{H_{36}}$), 4.89-4.50 (m, $\mathbf{H_{11}}$, $\mathbf{H_{13a}}$, $\mathbf{H_{26}}$), 4.19-3.88 (m, $\mathbf{H_{24}}$), 3.43-3.28 (m, $\mathbf{H_{13b}}$), 3.24-3.03 (m, $\mathbf{H_{10}}$, $\mathbf{H_{27}}$), 2.29-2.10 (m, $\mathbf{H_{17\text{-}19}}$), 2.10-1.97 (m, $\mathbf{H_{3}}$), 1.97-1.57 (m, $\mathbf{H_{16\text{-}21}}$), 1.55 (s, $\mathbf{H_{15b}}$), 1.54-1.43 (m, $\mathbf{H_{17\text{-}19}}$, $\mathbf{H_{4\text{-}9}}$, $\mathbf{H_{28\text{-}33}}$), 1.43-1.33 (m, $\mathbf{H_{17\text{-}19}}$, $\mathbf{H_{4\text{-}9}}$, $\mathbf{H_{28\text{-}33}}$), 1.33-1.21 (m, $\mathbf{H_{17\text{-}19}}$, $\mathbf{H_{4\text{-}9}}$, $\mathbf{H_{28\text{-}33}}$), 1.19 (s, $\mathbf{H_{15a}}$), 1.17-1.06 (m, $\mathbf{H_{22}}$).

Due to the occurrence of isomers, no integral values are given for the respective NMR signals. The sum of integrals matches the expected number of protons. The purity of the product was confirmed via SEC measurements.

^{13}C (100 MHz, CDCl$_3$) δ / ppm = 157.0 ($\mathbf{C_{12}/C_{25}}$), 156.8 ($\mathbf{C_{12}/C_{25}}$), 156.7 ($\mathbf{C_{12}/C_{25}}$), 139.2 ($\mathbf{C_{2}}$, $\mathbf{C_{35}}$), 114.2 ($\mathbf{C_{1}}$, $\mathbf{C_{36}}$), 83.4 ($\mathbf{C_{14b}}$), 77.7 ($\mathbf{C_{13a}}$), 76.2 ($\mathbf{C_{13b}}$), 73.4 ($\mathbf{C_{23}}$), 73.2 ($\mathbf{C_{23}}$), 70.5 ($\mathbf{C_{14a}}$), 70.3 ($\mathbf{C_{24}}$), 44.0 ($\mathbf{C_{20}}$), 43.1 ($\mathbf{C_{20}}$), 41.2 ($\mathbf{C_{10}/C_{27}}$), 41.1 ($\mathbf{C_{10}/C_{27}}$), 37.2 ($\mathbf{C_{17\text{-}19}}$), 37.0 ($\mathbf{C_{17}/C_{18}}$), 33.8 ($\mathbf{C_{3}}$), 31.4 ($\mathbf{C_{19}}$), 29.9 ($\mathbf{C_{4\text{-}9}/C_{28\text{-}33}}$), 29.8 ($\mathbf{C_{4\text{-}9}/C_{28\text{-}33}}$), 29.4 ($\mathbf{C_{4\text{-}9}/C_{28\text{-}33}}$), 29.2 ($\mathbf{C_{4\text{-}9}/C_{28\text{-}33}}$), 29.0 ($\mathbf{C_{4\text{-}9}/C_{28\text{-}33}}$), 28.9 ($\mathbf{C_{4\text{-}9}/C_{28\text{-}33}}$), 27.1 ($\mathbf{C_{15a}}$), 26.8 ($\mathbf{C_{4\text{-}9}/C_{28\text{-}33}}$), 26.7 ($\mathbf{C_{4\text{-}9}/C_{28\text{-}33}}$), 22.2 ($\mathbf{C_{15b}}$), 21.8 ($\mathbf{C_{17\text{-}19}}$), 21.3 ($\mathbf{C_{22}}$), 21.0 ($\mathbf{C_{22}}$), 20.9 ($\mathbf{C_{22}}$), 20.6 ($\mathbf{C_{17}/C_{18}}$).

IR (ATR platinum diamond): ṽ / cm^{-1} = 3331, 3077, 2973, 2925, 2854, 1688,1641, 1531, 1462, 1444, 1412, 1373, 1248, 1172, 1145, 1072, 1011, 994, 943, 908, 817, 775, 723, 631, 555.

ESI-MS: $[M+H]^+$ calc. 567.4368, detected 567.4362.

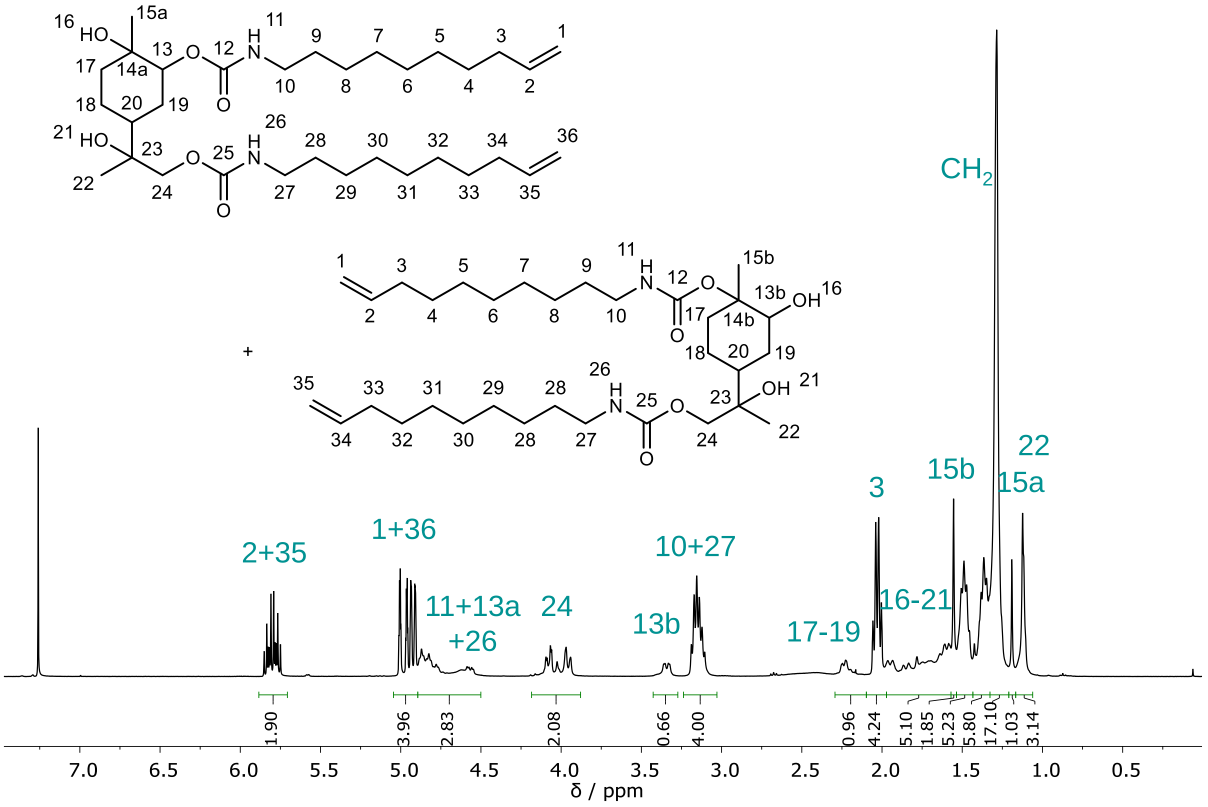

Supplementary Figure 58. ^{1}H NMR spectrum of 19, measured in CDCl$_3$.

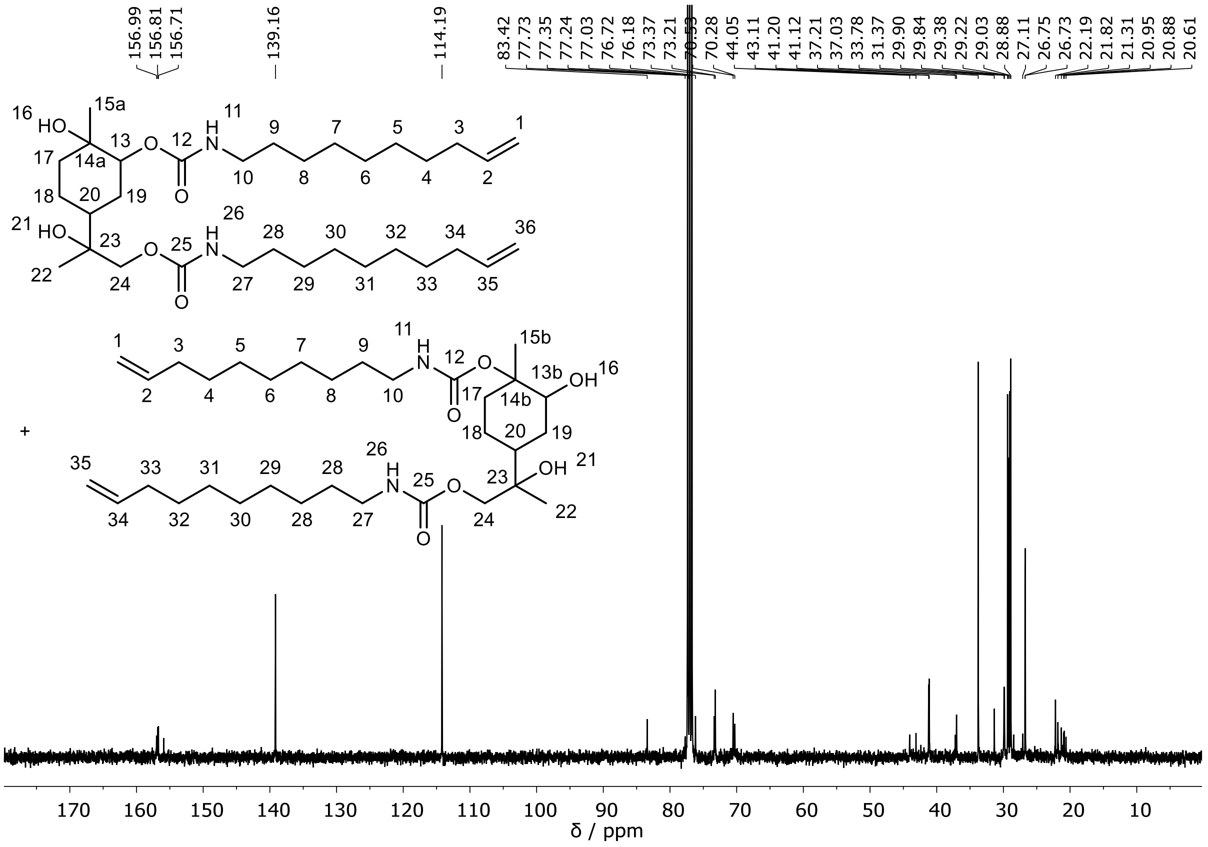

Supplementary Figure 59. ^{13}C NMR spectrum of 19, measured in CDCl$_3$. Assignment within the figure is omitted for higher clarity.

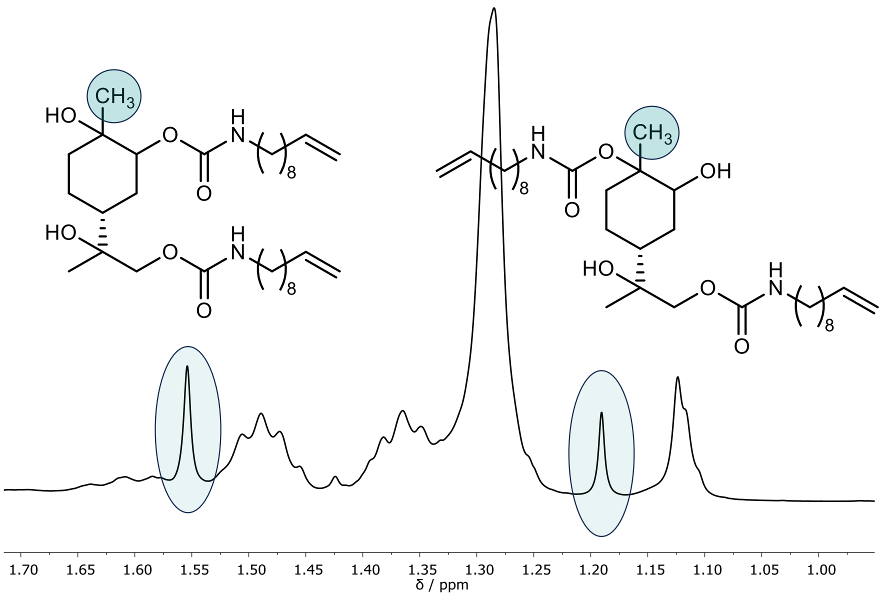

Supplementary Figure 60. Observation of different regioisomers of compound 19 via NMR spectroscopy.

As compound **18** was not sufficiently volatile to analyze via GC-FID, the occurring of regioisomers could only be observed via NMR measurements. Due to overlapping signals, no determination of a regioisomeric ratio was possible.

4.5 Synthesis of dithiol 22

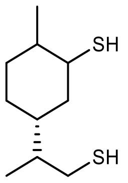

$C_{10}H_{20}S_2$
204.39 g/mol

10.0 g (73.4 mmol, 1.00 equiv.) (*R*)-limonene were reacted with 13.1 mL (14.0 g, 184 mmol, 2.50 equiv.) thioacetic acid for 18 h. After complete reaction (reaction control via TLC), the residue was dissolved in ethyl acetate (10 mL) and washed with brine (3×20 mL). The aqueous phase was extracted with ethyl acetate (20 mL) and the combined organic phases were dried over Na_2SO_4. After evaporation of the solvent, the crude dithioacetic ester was transesterified by adding 6.0 mL methanol (1.5 mol, 20 equiv.) and 2.0 g TBD (14.7 mmol, 0.20 equiv.) and stirring under reflux conditions and Argon atmosphere overnight. Excess methanol was removed under reduced pressure. After purification via column chromatography (*n*-hexane/ ethyl acetate 30:1), 395 mg (1.93 mmol, 2.6%) of a pure product fraction (99% according to GC-FID) were obtained. A second fraction of 6.08 g containing mainly the product (90% purity according to GC-FID) was purified by a second column chromatography (*n*-hexane/ethyl acetate 30:1), yielding 378 mg (1.85 mmol, 2.5%) of a pure product fraction (>99% according to GC-FID).

From GC-FID measurements, two isomers were observed in a ratio of 89:11.

R_f (dichloromethane) = 0.60, visualized by staining with Seebach solution.

^{1}H (400 MHz, $CDCl_3$) δ / ppm = 3.45-3.27 (m, **H_2**), 2.67-2.52 (m, **H_{11}**), 2.49-2.33 (m, **H_{11}**), 2.05-1.95 (m, **H_{minor}**), 1.90-1.74 (m, **H_7**, **H_8**), 1.74-1.61 (m, **H_3**, **H_6**), 1.61-1.51 (m, **H_7**), 1.51-1.36 (m, **H_5**, **H_9**), 1.33-1.21 (m, **H_1**, **H_{12}**), 1.19-1.00 (m, **H_6**), 1.00-0.84 (m, **H_4**, **H_{10}**).

Due to the occurrence of isomers, no integral values are given for the respective NMR signals and signals of the minor isomer are not assigned. The sum of integrals matches the expected number of protons. The purity of the product was confirmed via GC-FID measurements.

^{13}C (100 MHz, $CDCl_3$) δ / ppm = 46.3 (**C_2**), 44.1 (**C_2**), 44.0 (**C_2**), 43.3 (**C_{minor}**), 41.8 (**C_{minor}**), 41.7 (**C_{minor}**), 41.4 (**C_{minor}**), 41.0 (**C_{minor}**), 40.8 (**C_9**), 39.4 (**C_7**), 37.2 (**C_7**), 36.3 (**C_3**), 35.5 (**C_{minor}**), 35.4 (**C_{minor}**), 34.0 (**C_8**), 33.8 (**C_8**), 30.5 (**C_6**), 30.4 (**C_{minor}**), 29.7 (**C_{11}**), 29.5 (**C_{11}**), 28.4 (**C_6**), 28.3 (**C_5**), 28.2 (**C_5**), 20.9 (**C_{minor}**), 20.6 (**C_4**), 15.5 (**C_{10}**), 15.4 (**C_{10}**).

IR (ATR platinum diamond): ṽ / cm^{-1} = 2955, 2919, 2868, 2852, 2562, 1452, 1375, 1331, 1309, 1292, 1268, 1238, 1170, 1104, 1022, 998, 979, 939, 906, 865, 811, 766, 720, 666, 623, 524, 477, 455, 419.

ESI-MS: $[M+H]^+$ calc. 205.1079, detected

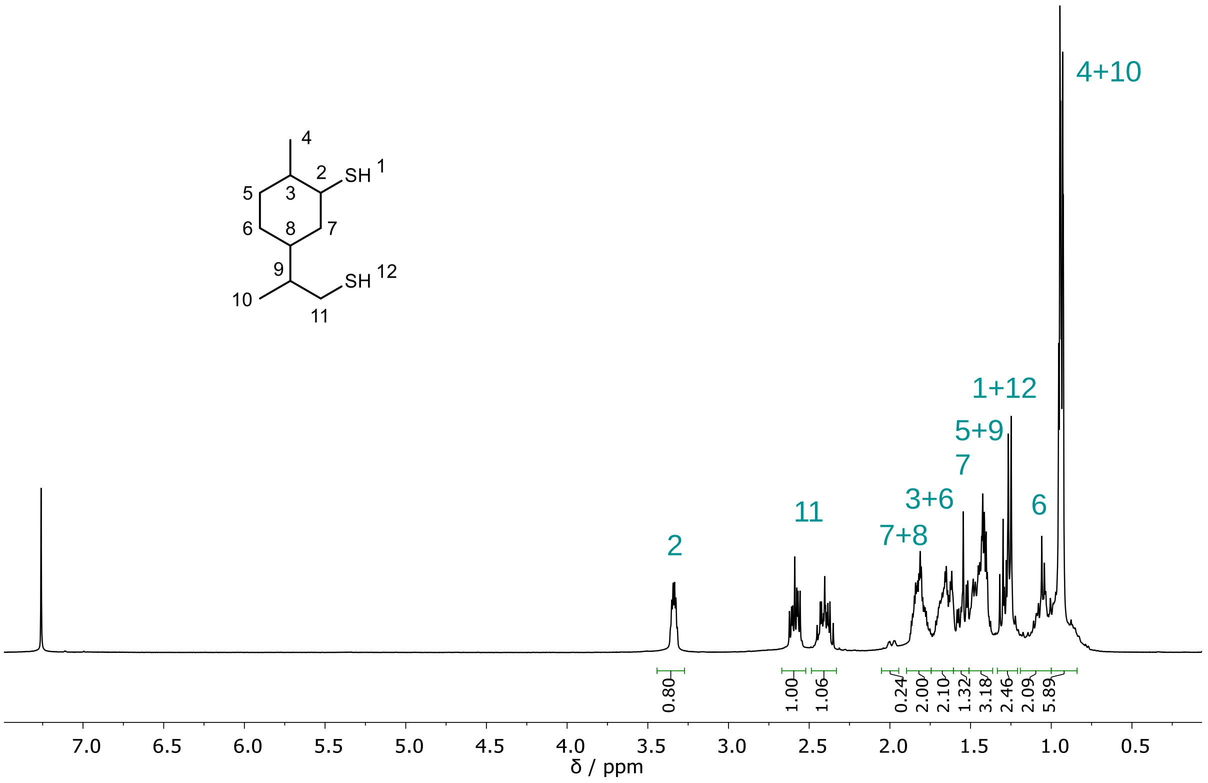

Supplementary Figure 61. **^{1}H NMR spectrum of 22, measured in $CDCl_3$.**

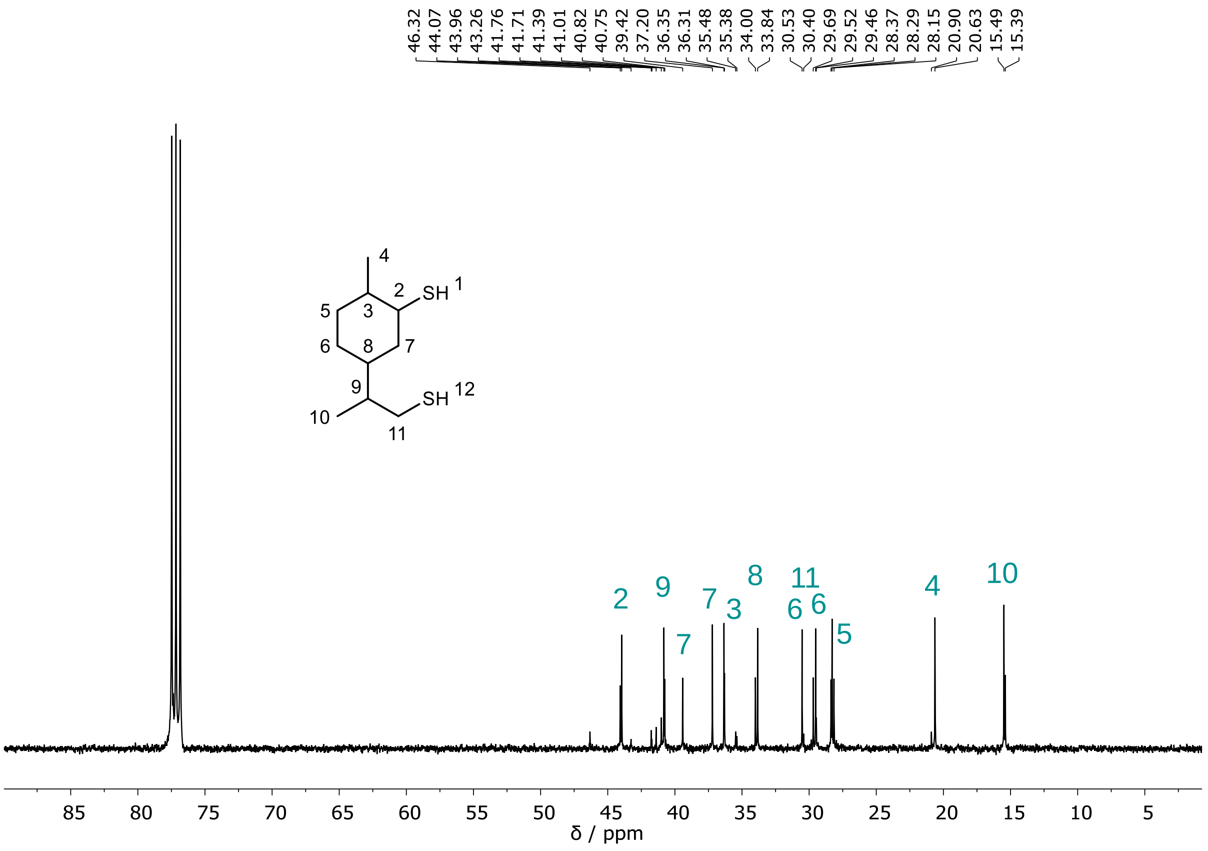

Supplementary Figure 62. **^{13}C NMR spectrum of 22, measured in $CDCl_3$.**

4.6 Synthesis of thiourea catalysts

N-(3,5-Bis(trifluormethyl)phenyl)-*N*´-cyclohexyl thiourea 9

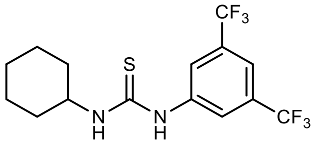

$C_{15}H_{16}F_6N_2S$
370.36 g/mol

2.00 g (7.38 mmol, 1.00 equiv.) 1-Isothiocyanato-3,5-bi(trifluormethyl)benzene were dissolved in 7.5 mL ethyl acetate. 0.94 mL (810 mg, 8.1 mmol, 1.1 equiv.) cyclohexylamine were added and the mixture was stirred at room temperature until TLC (cyclohexane/ ethyl acetate 2:1) detected full conversion. Afterwards, 10 mL water and 10 mL ethyl acetate were added and the phases were separated. The aqueous phase was extracted with ethyl acetate (2 × 25 mL) and washed with water (2 × 25 mL). After drying over Na_2SO_4, the solvent was removed under reduced pressure and 2.65 g (7.16 mmol, 97%) of the product were obtained as colorless solid.

R_f (cyclohexane/ethyl acetate 2:1) = 0.72, visualized by staining with permanganate solution.

^{1}H (400 MHz, DMSO-*d*$_6$) δ / ppm = 9.85 (br s, 1H, **H_7**), 8.23 (s, 2H, **H_9**), 8.14 (br s, 1H, **H_5**), 7.71 (s, 1H, **H_{11}**), 4.10 (m, 1H, **H_4**), 2.05-1.83 (m, 2H, **H_3**), 1.78-1.65 (m, 2H, **H_2**), 1.66-1.50 (m, 1H, **H_1**), 1.42-1.09 (m, 5H, **$H_{1\text{-}3}$**).

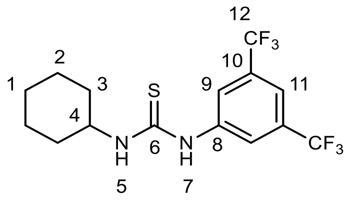

^{13}C (100 MHz, DMSO-*d*$_6$) δ / ppm = 179.2 (**C_6**), 142.0 (**C_8**), 130.1 (q, ^2J = 32.2 Hz, **C_{10}**), 123.2 (q, ^1J = 273.6 Hz, **C_{12}**), 121.7 (**C_9**), 115.8 (**C_{11}**), 52.3 (**C_4**), 31.6 (**C_3**), 25.1 (**C_1**), 24.4 (**C_2**).

IR (ATR platinum diamond): ṽ / cm^{-1} = 3281, 3189, 3163, 3040, 3001, 2933, 2860, 1791, 1622, 1553, 1526, 1467, 1437, 1382, 1364, 1336, 1269, 1176, 1127, 1105, 1039, 1024, 941, 904, 888, 862, 847, 810, 754, 706, 679, 618, 587, 516, 468, 454, 420.

ESI-MS: $[M+H]^+$ calc. 371.1011, detected 371.1003.

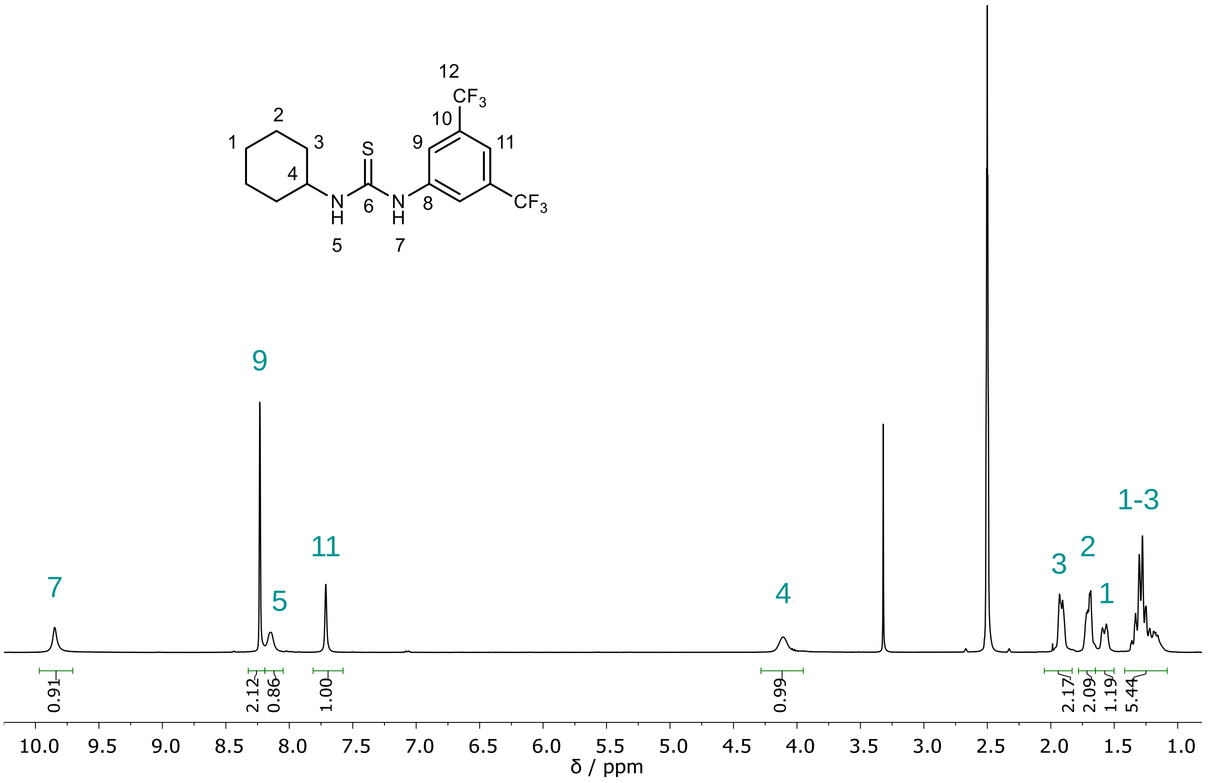

Supplementary Figure 63. **^{1}H NMR spectrum of 9, measured in DMSO-*d*$_6$.**

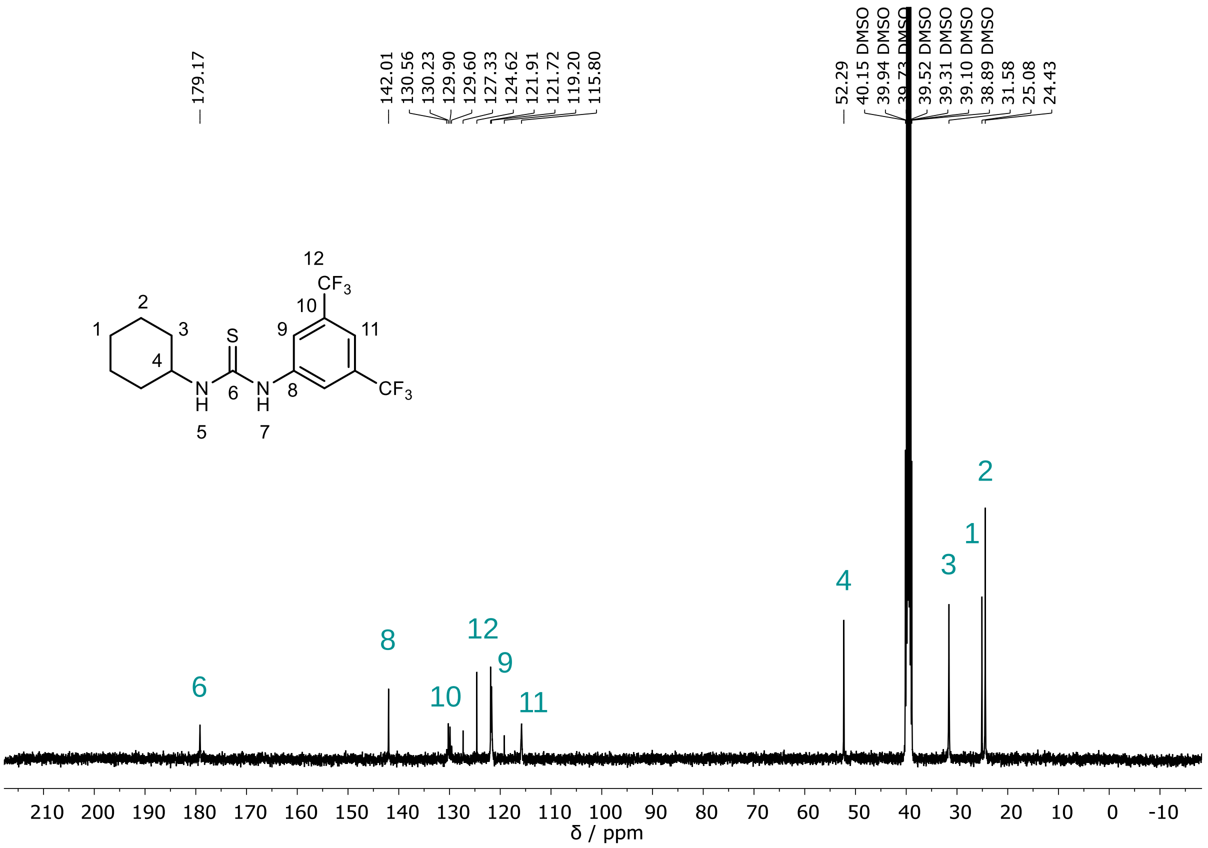

Supplementary Figure 64. **^{13}C NMR spectrum of 9, measured in DMSO-*d*$_6$.**

Synthesis of thiourea 10

4-(*n*-Hexylthio) aniline 26

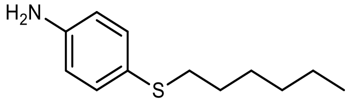

$C_{12}H_{19}NS$
209.35 g/mol

The product was prepared according to a literature-known procedure.[3]

6.02 g (48.1 mmol, 1.00 equiv.) 4-amino thiophenol were dissolved in 150 mL ethanol (0.33 M). To this solution, 1.90 g (47.5 mmol, 0.988 equiv.) NaOH were added. Afterwards, 6.70 mL (7.91 g, 47.9 mmol, 1.00 equiv.) 1-bromohexane were added within the course of 1 h. The mixture was stirred at room temperature overnight and afterwards poured into 360 mL ice-cold water. The product was extracted with diethyl ether. The organic phase was washed with saturated Na_2CO_3 solution (3 × 160 mL) and with aqueous NH_4Cl (3 × 160 mL) solution and dried over Na_2SO_4. After removal of the solvent under reduced pressure, the brown oil was purified via column chromatography (cyclohexane/ethyl acetate 95:5 → 7:3). The product was yielded as brownish oil in a yield of 7.20 g (34.4 mmol, 71%).

R_f (cyclohexane/ethyl acetate 7:3) = 0.71, visualized by staining with vanillin solution.

^{1}H (400 MHz, DMSO-*d*$_6$) δ / ppm = 7.13-7.03 (m, 2H, **H_3**), 6.58-6.41 (m, 2H, **H_4**), 5.20 (s, 2H, **H_1**), 2.67 (t, *J* = 7.2 Hz, 2H, **H_6**), 1.55-1.38 (m, 2H, **H_7**), 1.33 (ddt, *J* = 13.9, 9.3, 6.3 Hz, 2H, **H_8**), 1.28-1.13 (m, 4H, **H_9**, **H_{10}**), 0.93-0.75 (m, 3H, **H_{11}**).

^{13}C (100 MHz, DMSO-*d*$_6$) δ / ppm = 148.2 (**C_2**), 133.5 (**C_3**), 119.1 (**C_5**), 114.4 (**C_4**), 35.7 (**C_6**), 30.8 (**C_9**), 28.8 (**C_7**), 27.6 (**C_8**), 22.0 (**C_{10}**), 13.9 (**C_{11}**).

IR (ATR platinum diamond): ṽ / cm^{-1} = 3452, 3360, 3211, 3025, 2953, 2924, 2854, 1730, 1619, 1597, 1494, 1464, 1422, 1376, 1277, 1176, 1124, 1096, 1010, 820, 725, 681, 623, 514.

ESI-MS: $[M+H]^+$ calc. 210.1311, detected 210.1306.

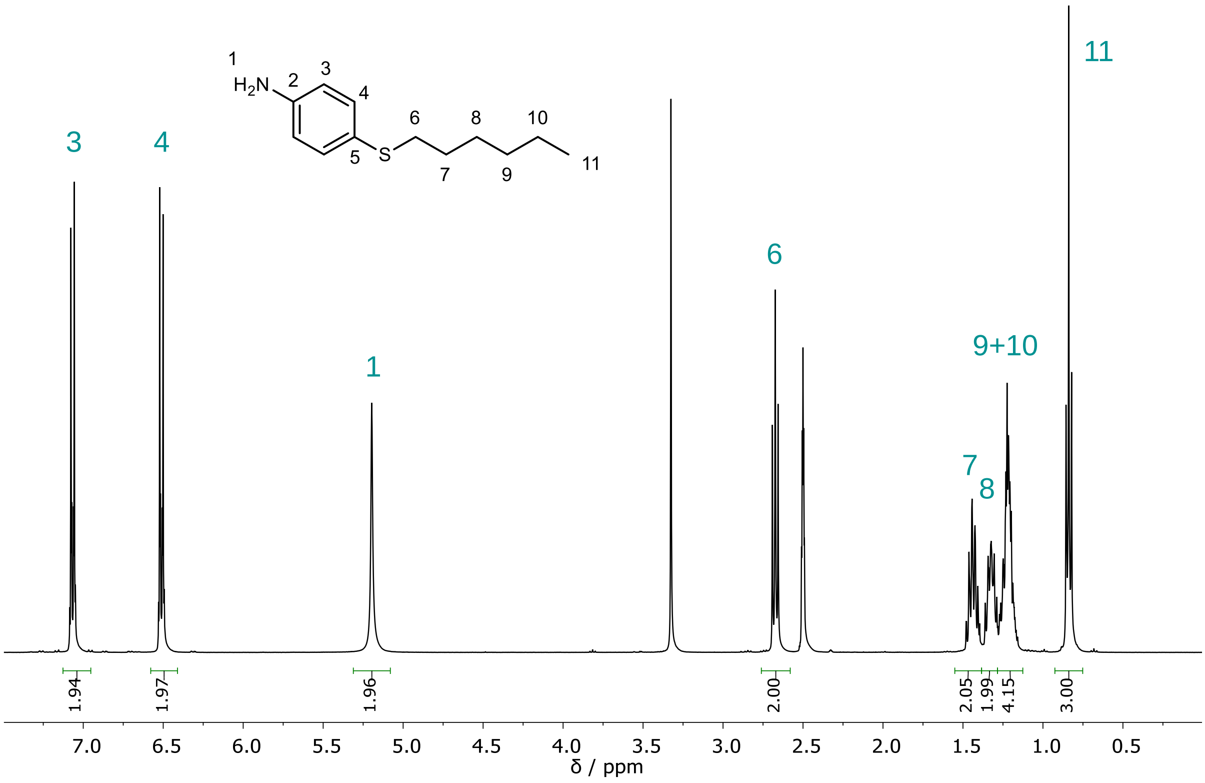

Supplementary Figure 65. ^{1}H NMR spectrum of 26, measured in DMSO-d_6.

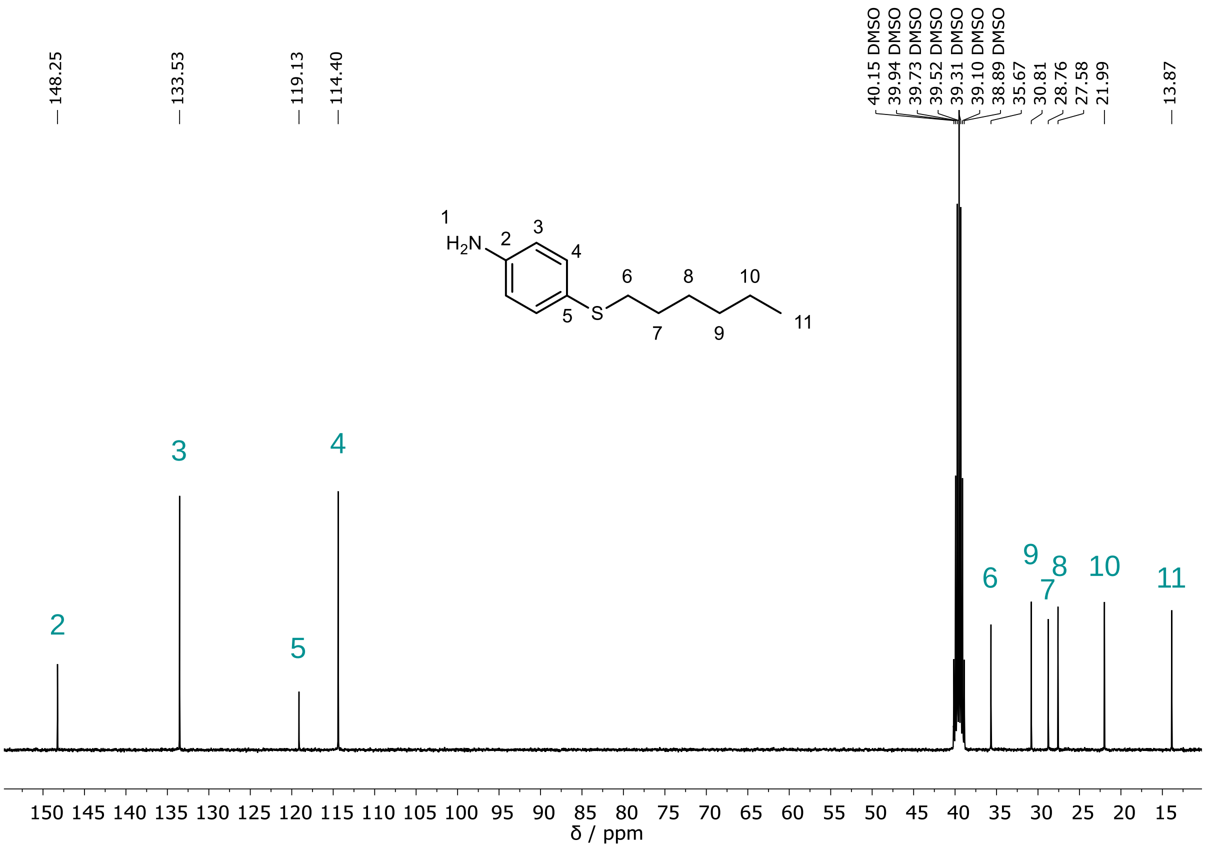

Supplementary Figure 66. ^{13}C NMR spectrum of 26, measured in DMSO-d_6.

4-(n-Hexylsulfonyl) aniline 27

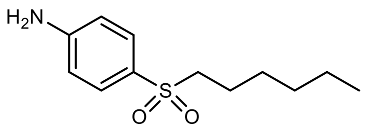

$C_{12}H_{19}NO_2S$
241.35 g/mol

The product was prepared according to a literature-known procedure.[3]

4.00 g 4-(*n*-hexylthio) aniline **26** (19.1 mmol, 1.00 equiv.) were dissolved in 38 mL acetonitrile (0.5 M) and 19.5 mL of aqueous hydrogen peroxide solution (30 wt-% in water, 22 g, 191 mmol, 10.0 equiv.) were added. The reaction mixture was stirred at 40 °C for 23 hours. Then, the reaction mixture was cooled to room temperature, the same amount of hydrogen peroxide was added and the mixture was stirred further at 40 °C for 17 hours. Afterwards, 38 mL of saturated Na_2SO_3 solution were added slowly, while cooling the mixture with a water bath. The organic phase was separated and the aqueous phase was extracted with ethyl acetate (2×40 mL). The combined organic phases were dried over Na_2SO_4 and the solvent was removed under reduced pressure. Because of the formation of two phases during evaporation, the mixture was diluted with dichloromethane, washed with water and the aqueous phase was extracted with dichloromethane (2 × 30 mL). The combined organic phases were dried over Na_2SO_4 and the solvent was again removed under reduced pressure. 4.61 g (19.1 mmol, quant.) of the product were obtained as a brown solid.

R_f (cyclohexane/ethyl acetate 2:1) = 0.25, visualized by staining with vanillin solution.

^{1}H (400 MHz, DMSO-*d*$_6$) δ / ppm = 7.54-7.35 (m, 2H, **H_4**), 6.75-6.54 (m, 2H, **H_3**), 6.10 (s, 2H, **H_1**), 3.15-2.97 (m, 2H, **H_6**), 1.64-1.39 (m, 2H, **H_7**), 1.37-1.08 (m, 6H, **H_{8-10}**), 0.82 (t, *J* = 6.7 Hz, 3H, **H_{11}**).

^{13}C (100 MHz, DMSO-*d*$_6$) δ / ppm = 153.5 (**C_5**), 129.5 (**C_4**), 123.7 (**C_2**), 112.7 (**C_3**), 55.4 (**C_6**), 30.7 (**C_9**), 27.1 (**C_8**), 22.6 (**C_7**), 21.8 (**C_{10}**), 13.8 (**C_{11}**).

IR (ATR platinum diamond): ṽ / cm^{-1} = 3468, 3372, 3247, 3223, 2954, 2923, 2871, 2857, 1906, 1630, 1594, 1503, 1459, 1438, 1410, 1378, 1322, 1301, 1271, 1241, 1217, 1184, 1127, 1084, 1013, 964, 945, 828, 767, 726, 701, 681, 633, 593, 563, 526, 489, 441, 421.

ESI-MS: $[M+H]^+$ calc. 238.1260, detected 238.1256.

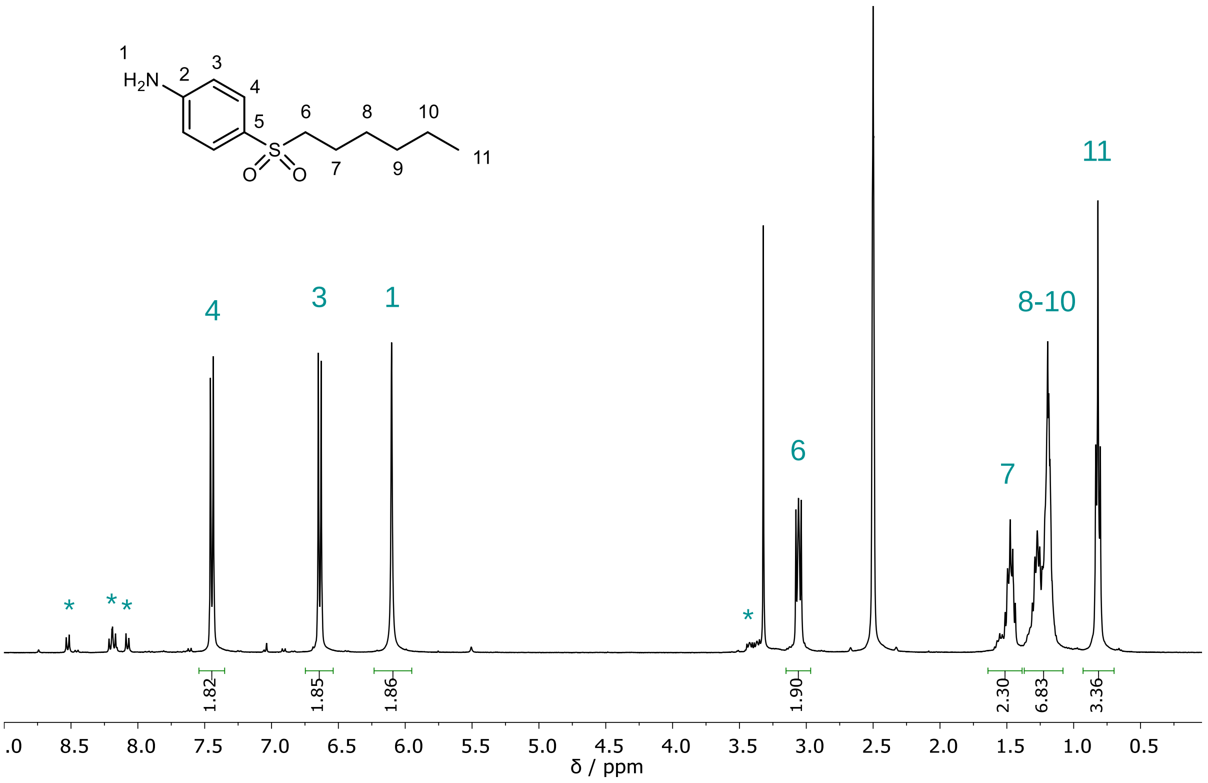

Supplementary Figure 67. ^{1}H NMR spectrum of 27, measured in DMSO-d_6. Signals marked with * can be attributed to a *N*-oxide side product.

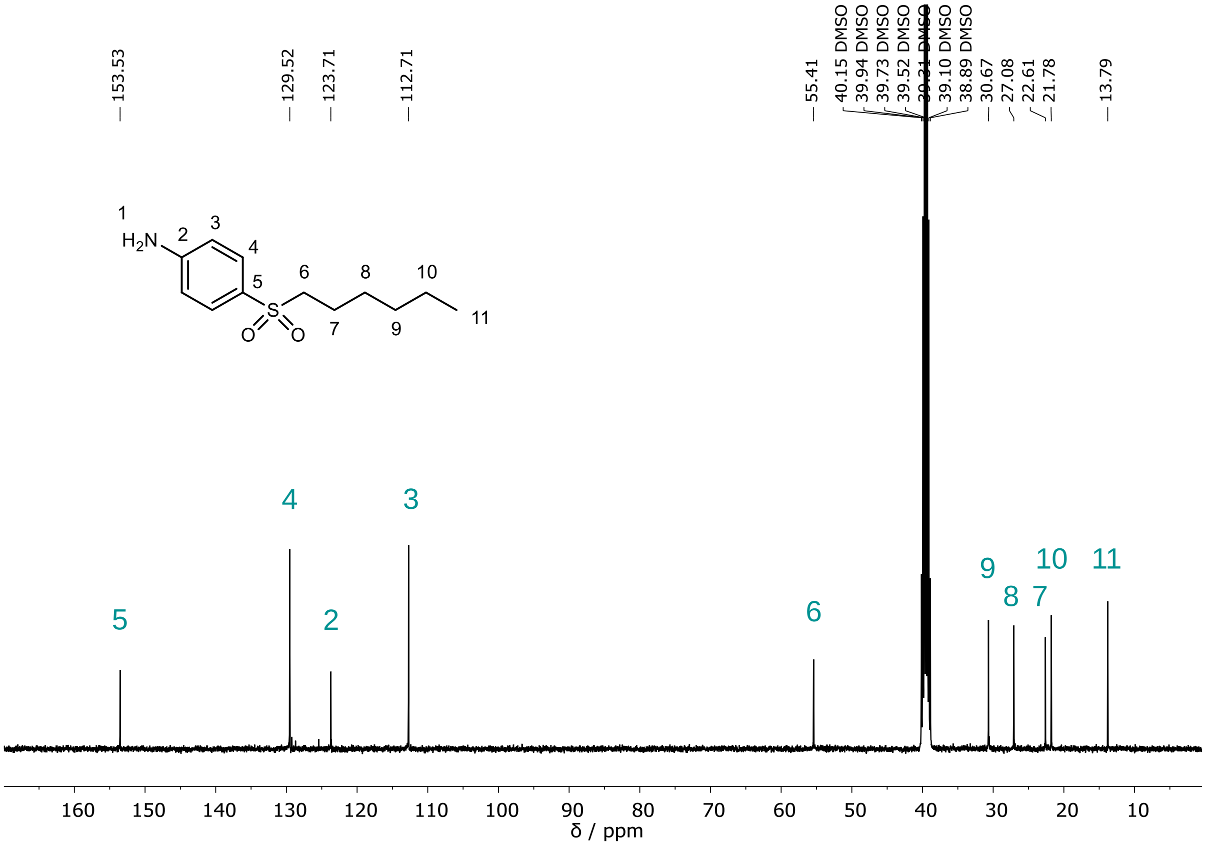

Supplementary Figure 68. ^{13}C NMR spectrum of 27, measured in DMSO-d_6.

4-(*n*-Hexylsulfonyl) formamidobenzene 28

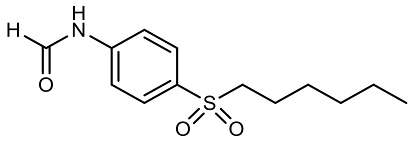

$C_{13}H_{19}NO_3S$
269.36 g/mol

To 4.61 g (19.1 mmol, 1.00 equiv.) of dimethyl 5-formamidoisophtalate **27**, 7.21 mL (8.80 g, 191 mmol, 10.0 equiv.) formic acid were added. The mixture was stirred at 60 °C for 24 hours. Subsequently, the remaining formic acid and water were removed under reduced pressure. Afterwards, 5.11 g (19.0 mmol, 99%) of the product were obtained as a brown solid. The product was used directly for the synthesis of **29** without further purification.

R_f (cyclohexane/ethyl acetate 2:1) = 0.10, visualized by staining with vanillin solution.

^{1}H (400 MHz, DMSO-*d*$_6$) δ / ppm = 10.65 (s, **H_{1a}**), 10.54 (d, *J* = 10.6 Hz, **H_{1b}**), 9.00 (d, *J* = 10.6 Hz, **H_{3b}**), 8.37 (d, *J* = 1.7 Hz, **H_{3a}**), 7.91-7.70 (m, **H_{5a}, H_6**), 7.51-7.37 (m, **H_{5b}**), 3.31-3.10 (m, 2H, **H_8**), 1.66-1.39 (m, 2H, **H_9**), 1.39-1.05 (m, 6H, **H_{10-12}**), 0.93-0.71 (m, 3H, **H_{13}**).

Due to the occurrence of rotamers, no integral values are given for the aromatic and carbonyl ^{1}H NMR signals. The signals of the rotamers are labelled with a and b for the major and minor rotamer, respectively.

^{13}C (100 MHz, DMSO-*d*$_6$) δ / ppm = 162.7 (**C_{2b}**), 160.4 (**C_{2a}**), 143.3 (**C_{4b}**) 142.7 (**C_{4a}**), 133.3 (**C_{7a}**) 133.2 (**C_{7b}**), 129.4 (**C_{6b}**), 129.1 (**C_{6a}**), 119.1 (**C_{5a}**), 116.8 (**C_{5b}**), 54.8 (**C_8**), 30.6 (**C_{11}**), 27.1 (**C_{10}**), 22.3 (**C_9**), 21.8 (**C_{12}**), 13.8 (**C_{13}**).

IR (ATR platinum diamond): ṽ / cm^{-1} = 3360, 3263, 3202, 3070, 3030, 2993, 2953, 2934, 2921, 2871, 2858, 1750, 1682, 1590, 1519, 1490, 1468, 1456, 1416, 1380, 1273, 1236, 1217, 1193, 1135, 1084, 1033, 1012, 966, 948, 915, 889, 833, 813, 767, 744, 724, 707, 688, 618, 592, 565, 536, 496, 462, 441, 423.

ESI-MS: $[M+H]^+$ calc. 270.1158, detected 270.1155.

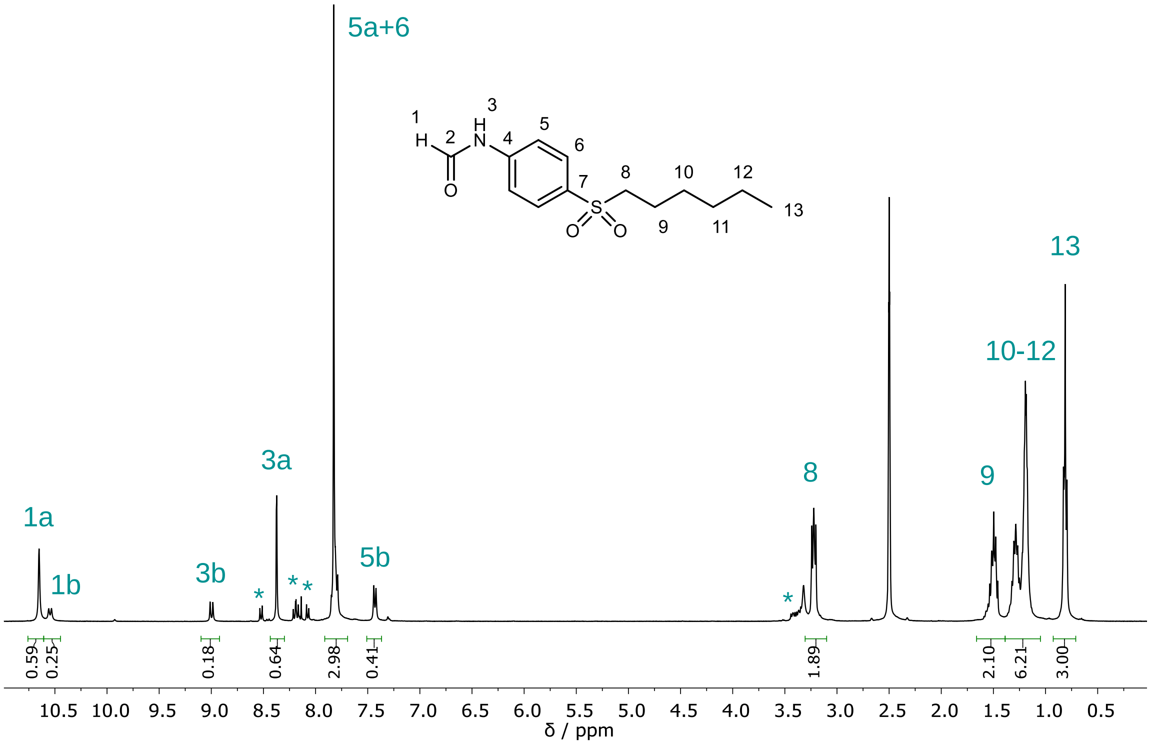

Supplementary Figure 69. ¹H NMR spectrum of 28, measured in DMSO-*d*₆. * can be attributed to a side product from the synthesis of **27**. Rotamers are labelled as a and b.

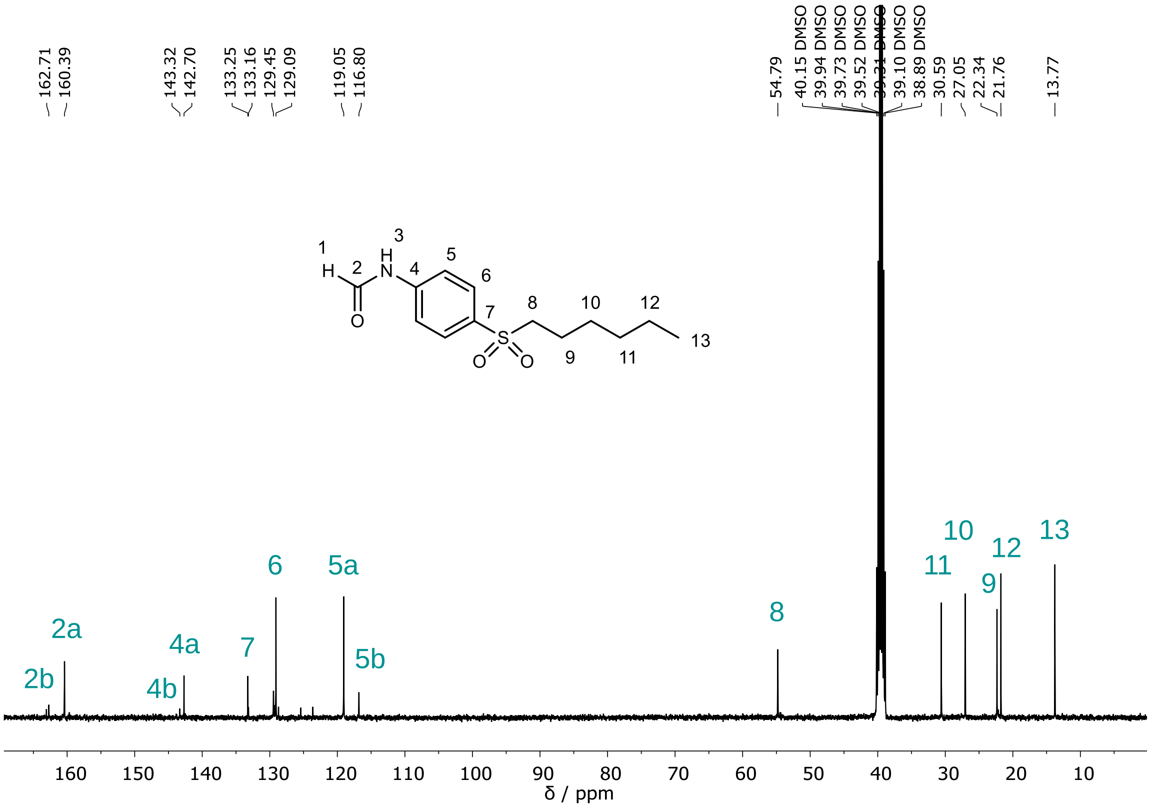

Supplementary Figure 70. ¹³C NMR spectrum of 27, measured in DMSO-*d*₆. Rotamers are labelled as a and b.

4-(n-Hexylsulfonyl) isocyanobenzene 29

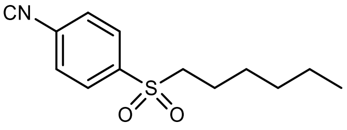

$C_{13}H_{17}NO_2S$
251.34 g/mol

1.76 g (6.55 mmol, 1.00 equiv.) 4-(n-hexylsulfonyl) formamidobenzene **28** were dissolved in 20 mL dichloromethane (0.33 M) and 2.85 mL (2.05 g, 20.3 mmol, 3.10 equiv.) diisoproplyl amine (DIPA) were added. The reaction mixture was cooled to 0 °C and 777 µL (1.31 g, 8.51 mmol, 1.30 equiv.) phosphorus oxychloride were added dropwise while keeping the temperature at 0 °C. Afterwards, the reaction was stirred at room temperature for 2 hours. Purification was performed via flash column chromatography, in which the reaction mixture was added dropwise directly onto the dry silica loaded column (height ca. 10 cm, ø ca. 5 cm) to quench the remaining phosphorus oxychloride. Then, the product was purified by eluting with dichloromethane with 3 vol.-% triethyl amine. 1.20 g (9% impurity of DIPA, corresponds to 4.60 mmol, product, 70%) of the product were obtained as dark red solid.

^{1}H (400 MHz, DMSO-d_6) δ / ppm = 8.08-7.97 (m, 2H, **H_4**), 7.94-7.81 (m, 2H, **H_3**), 3.45-3.30 (m, 2H, **H_6**), 1.61-1.44 (m, 2H, **H_7**), 1.37-1.09 (m, 6H, **H_{8-10}**), 0.91-0.74 (m, 3H, **H_{11}**).

Further analysis was not performed due to the sensitivity of the product.

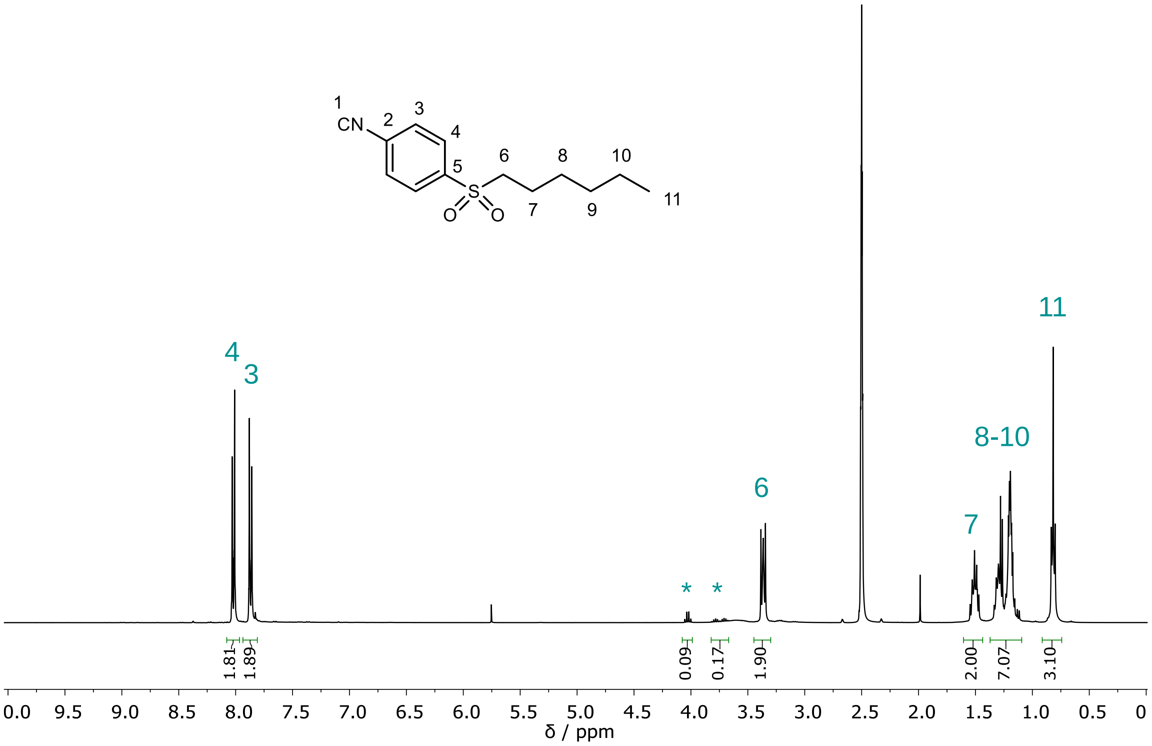

Supplementary Figure 71. ^{1}H NMR spectrum of 29, measured in DMSO-d_6. Signals marked with * can be attributed to residual DIPA.

***N*-(4-(*n*-Hexylsulfony)phenyl)-*N*′-cyclohexyl thiourea 10**

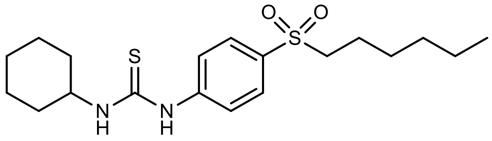

$C_{19}H_{30}N_2O_2S_2$
382.58 g/mol

197 mg (771 µmol, 0.125 equiv.) elemental sulfur were suspended in 6.9 mL methanol (0.90 M corresponding to isocyanide) and 711 µL (612 mg, 6.17 mmol, 1.00 equiv.) cyclohexylamine were added. 1.55 g (6.17 mmol, 1.00 equiv.) 4-(*n*-hexylsulfonyl) isocyanobenzene were added and the reaction mixture was stirred at room temperature for 17 hours. Afterwards, the solvent was removed under reduced pressure and the product was purified via column chromatography (cyclohexane/ethyl acetate 6:4 → 3:1). 1.49 g (3.89 mmol, 63%) of the product were obtained as slightly red solid.

R_f (cyclohexane/ethyl acetate 2:1) = 0.32, visualized by staining with vanillin solution.

^{1}H (400 MHz, DMSO-*d*$_6$) δ / ppm = 9.77 (bs, 1H, **H_7**), 8.12-7.97 (m, 1H, **H_5**), 7.85-7.79 (m, 2H, **H_{10}**), 7.78-7.73 (m, 2H, **H_9**), 4.18-4.04 (m, 1H, **H_4**), 3.26-3.17 (m, 2H, **H_{12}**), 1.99-1.84 (m, 2H, **H_3**), 1.76-1.65 (m, 2H, **H_2**), 1.64-1.09 (m, 18H, **$H_{1\text{-}3}$**, **$H_{13\text{-}16}$**), 0.88-0.77 (m, 3H, **H_{17}**).

^{13}C (100 MHz, DMSO-*d*$_6$) δ / ppm = 178.8 (**C_6**), 144.7 (**C_8**), 132.4 (**C_{11}**), 128.3 (**C_9**), 120.7 (**C_{10}**), 54.8 (**C_{12}**), 52.1 (**C_4**), 31.6 (**C_3**), 30.6 (**C_{15}**), 27.0 (**C_{14}**), 25.1 (**C_1**), 24.4 (**C_2**), 22.3 (**C_{13}**), 21.7 (**C_{16}**), 13.7 (**C_{17}**).

IR (ATR platinum diamond): ṽ / cm^{-1} = 3340, 3224, 3181, 3087, 3063, 2929, 2852, 1911, 1623, 1596, 1532, 1497, 1449, 1419, 1333, 1299, 1273, 1251, 1212, 1181, 1135, 1111, 1086, 1011, 981, 889, 876, 832, 772, 740, 722, 688, 666, 649, 621, 596, 573, 550, 530, 490, 443.

ESI-MS: $[M+H]^+$ calc. 383.1821, detected 383.1818.

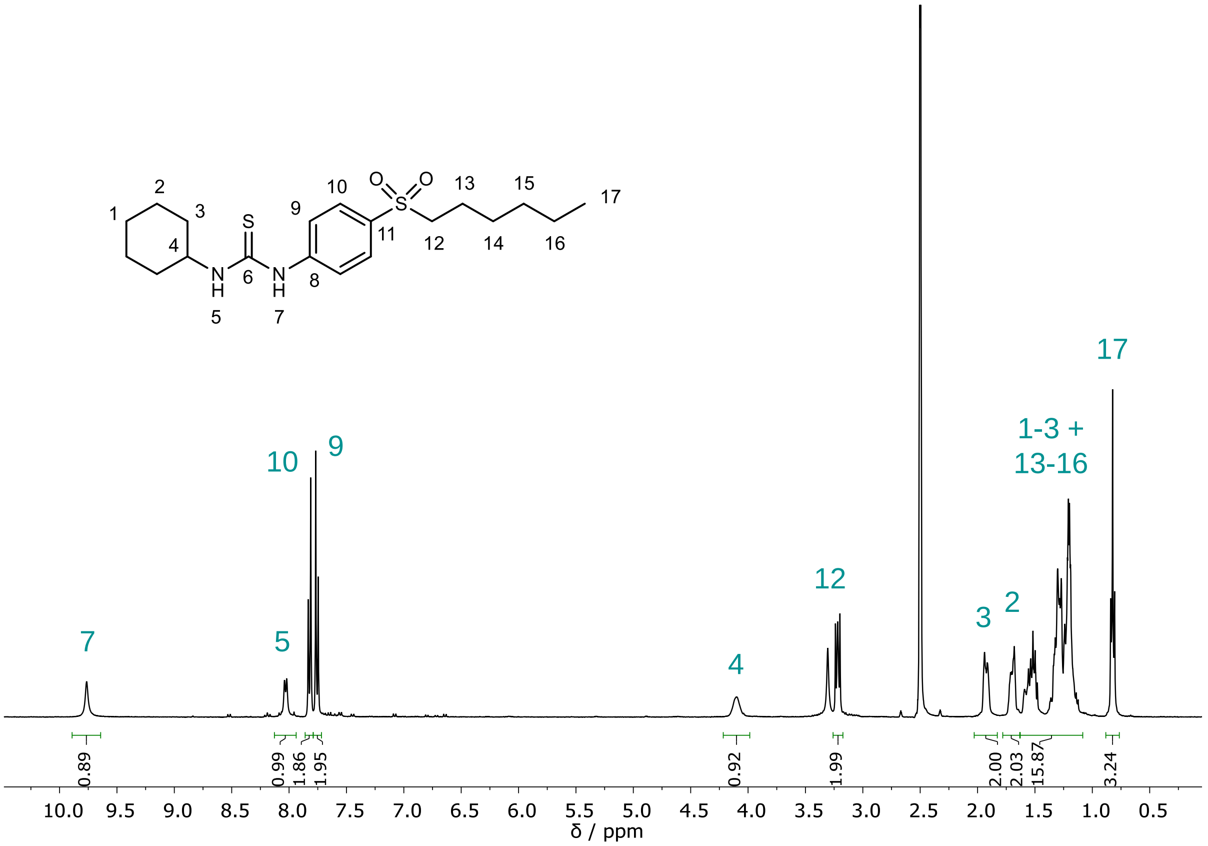

Supplementary Figure 72. ^{1}H NMR spectrum of 10, measured in DMSO-*d*$_6$.

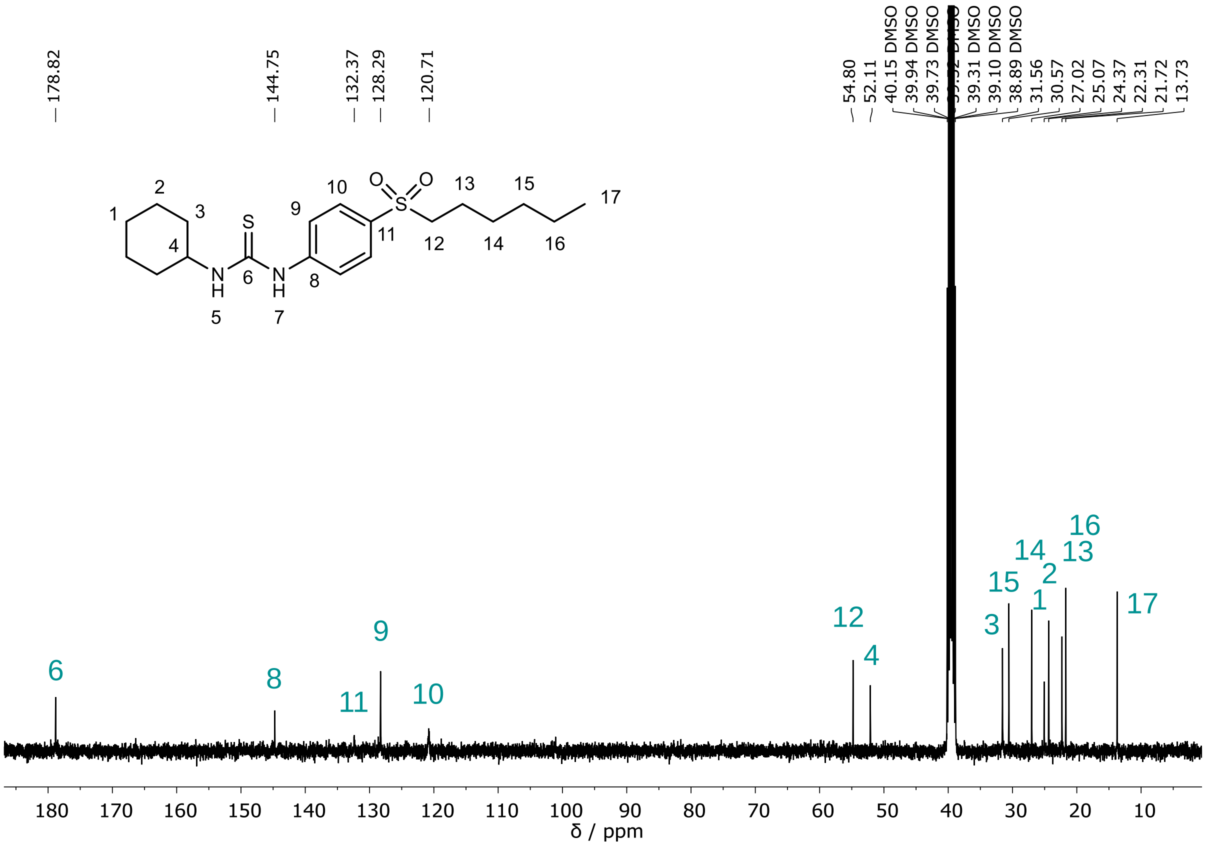

Supplementary Figure 73. ^{13}C NMR spectrum of 10, measured in DMSO-*d*$_6$.

N-(5-(Dimethylisophtalate))-*N´*-cyclohexyl thiourea 11

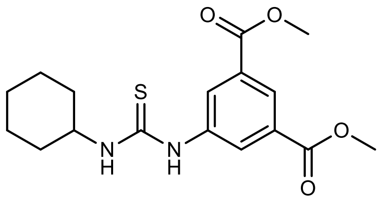

$C_{17}H_{22}N_2O_4S$
350.43 g/mol

5-Isocyano dimethylisophtalate was synthesized according to previous reports. The product was prepared according to a literature-known procedure.[3]

To a dispersion of 164 mg (0.639 mmol, 0.140 equiv.) elemental sulfur in 5.06 mL methanol (0.90 M corresponding to n(isocyanide)), 575 µL (498 mg, 5.02 mmol, 1.10 equiv.) cyclohexyl amine were added. 1.00 g (4.56 mmol, 1.00 equiv.) 5-isocyano dimethylisophtalate were added and the reaction mixture was stirred at room temperature for 4 days. Afterwards, 13 mL methanol were added and the mixture was left to crystallize for 3 days at room temperature. Filtration of the product, washing with a minimal amount of methanol and removal of the solvent yielded the 1.33 g (3.80 mmol, 83%) of the product as a slightly yellow powder.

R_f (cyclohexane/ethyl acetate 3:1) = 0.17, visualized by staining with vanillin solution.

^{1}H (400 MHz, DMSO-*d*$_6$) δ / ppm = 9.72 (bs, 1H, **H_7**), 8.43-8.34 (m, 2H, **H_9**), 8.16 (s, 1H, **H_{11}**), 7.92 (bs, 1H, **H_5**), 4.14-4.04 (m, 1H, **H_4**), 3.89 (s, 6H, **H_{13}**), 1.96-1.87 (m, 2H, **H_3**), 1.74-1.66 (m, 2H, **H_2**), 1.60-1.54 (m, 1H, **H_1**), 1.36-1.13 (m, 5H, **H_{1-3}**).

^{13}C (100 MHz, DMSO-*d*$_6$) δ / ppm = 179.4 (**C_6**), 165.3 (**C_{12}**), 141.1 (**C_8**), 130.1 (**C_{10}**), 126.7 (**C_9**), 124.1 (**C_{11}**), 52.5 (**C_{13}**), 52.2 (**C_4**), 31.7 (**C_3**), 25.1 (**C_1**), 24.4 (**C_2**).

IR (ATR platinum diamond): ṽ / cm^{-1} = 3329, 3162, 3020, 2927, 2857, 2847, 1727, 1711, 1598, 1530, 1435, 1383, 1344, 1312, 1250, 1197, 1184, 1144, 1110, 997, 981, 908, 892, 852, 821, 793, 756, 723, 690, 666, 633, 606, 548, 510, 485, 454, 433.

ESI-MS: $[M+H]^+$ calc. 351.1373, detected 351.1368.

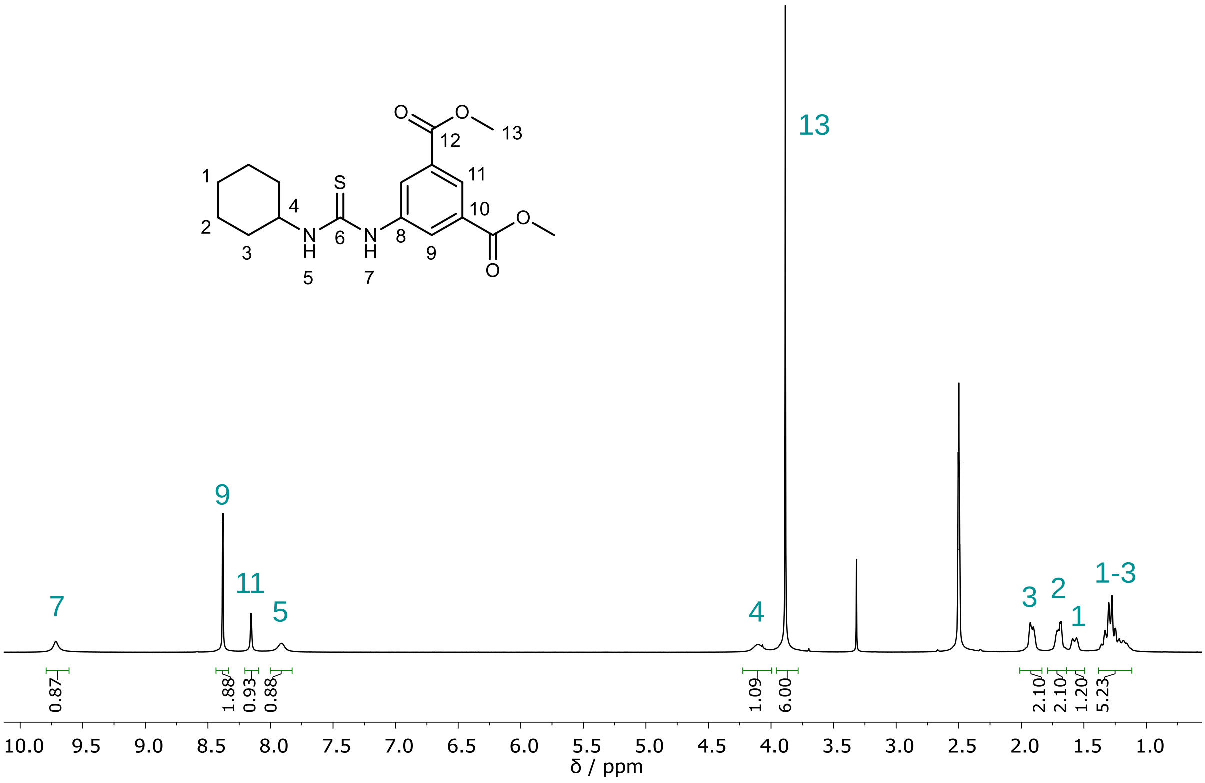

Supplementary Figure 74. ^{1}H NMR spectrum of 11, measured in DMSO-*d*$_6$.

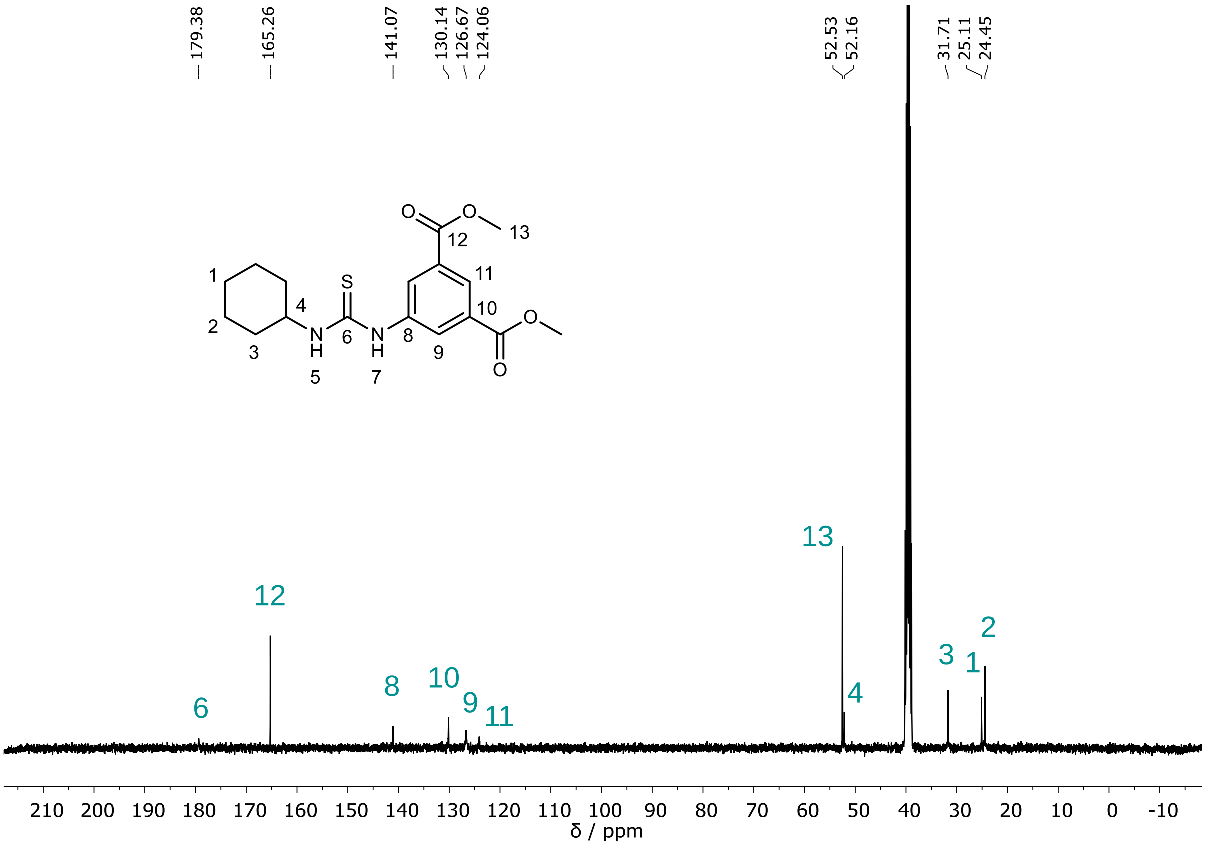

Supplementary Figure 75. ^{13}C NMR spectrum of 11, measured in DMSO-*d*$_6$.

5. Supplementary References

1. Hauenstein, O., Reiter, M., Agarwal, S., Rieger, B. & Greiner, A. Bio-based polycarbonate from limonene oxide and CO_2 with high molecular weight, excellent thermal resistance, hardness and transparency. *Green Chem.* **18,** 760–770; 10.1039/C5GC01694K (2016).
2. Löser, P. S., Rauthe, P., Meier, M. A. R. & Llevot, A. Sustainable catalytic rearrangement of terpene-derived epoxides: towards bio-based biscarbonyl monomers. *Phil. Trans. R. Soc. A* **378,** 20190267; 10.1098/rsta.2019.0267 (2020).
3. Nickisch, R., Gabrielsen, S. M. & Meier, M. A. R. Novel Access to Known and Unknown Thiourea Catalyst via a Multicomponent-Reaction Approach. *ChemistrySelect* **5,** 11915–11920; 10.1002/slct.202003336 (2020).
